# Synthesis of Cyclotetrapeptides Analogues to Natural Products as Herbicides

**DOI:** 10.3390/molecules27217350

**Published:** 2022-10-29

**Authors:** Camila Irabuena, Laura Posada, Luciana Rey, Laura Scarone, Danilo Davyt, Juana Villalba, Gloria Serra

**Affiliations:** 1 Laboratorio de Química Farmacéutica, Departamento de Química Orgánica, Facultad de Química, Universidad de la República, General Flores 2124, Montevideo 11800, Uruguay; 2 Estación Experimental Dr. Mario A. Cassinoni, Facultad de Agronomía, Universidad de la República, Ruta 3 Km 363, Paysandú 60000, Uruguay

**Keywords:** cyclopeptides, herbicides, synthesis, peptides

## Abstract

The synthesis of cyclotetrapeptides analogues of the natural products tentoxin and versicotide D was achieved in good yield by solid phase peptide synthesis (SPPS) of their linear precursors and solution phase cyclization. All the cyclopeptides and several open precursors were evaluated as herbicides. Five cyclopeptides and five lineal peptides showed a significant inhibition (>70%) of Ryegrass seed’s radicle growth at 67 μg/mL. The evaluation at lower concentrations (4–11 μM) indicates two cyclopeptides analogs of tentoxin, which present one (*N*-Methyl-d-Phe), and two *N-*MeAA (*N*-Methyl-Ala and *N*-Methyl-Phe), respectively, as the most active of them, showing remarkable phytotoxic activity. In two cases, the open precursors are as active as their corresponding cyclopeptide. However, many linear peptides are inactive and their cyclization derivatives showed herbicidal activity. In addition, two cyclopeptide analogues of versicotide D showed more improved activity than the natural product. The results indicate that the peptide sequence, the amino acid stereochemistry and the presence of *N*-methyl group have important influence on the phytotoxic activity. Moreover, several compounds could be considered as lead candidates in the development of bioherbicides.

## 1. Introduction

Agrochemicals play a key role in agriculture as their use has dramatically increased productivity. Weeds represent one of the most important pests that need to be controlled. In fact, about 50% of the commercial pesticides used worldwide are herbicides [1]. However, many factors are causing an urgent need for the development of novel herbicides. In the first place, the toxicity and long-term impact on human health and the environment of extensively used agrochemicals, such as glyphosate, have been deeply studied during the last decades [2,3,4]. As a result, many agrochemicals have been banned by governmental agencies in many countries [5]. Furthermore, agrochemicals have caused the eutrophication of water bodies, with environmental consequences, such as the increase in cyanobacteria blooms [6], which produce harmful toxins [7], affecting animal and human health. On the other hand, many herbicides have become ineffective by the development of weed resistance [8,9,10], and the discovery of new ones during the recent decades have been scarce [11,12,13].

Natural products have long been a source of inspiration in the discovery of bioactive molecules. Furthermore, natural products could provide an environmentally friendlier approach to weed management [14,15,16]. In the search for herbicides with novel modes of action and safer for both human health and the environment, plants, fungi extracts and metabolites have been investigated as bioherbicides [17].

Naturally occurring cyclic peptides and synthetic cyclic peptides inspired in natural products have found application in a large variety of fields, such as drug discovery [18], imaging [19] and materials chemistry [20]. Relevant features that justify this fact are their great binding affinity, low toxicity, and the capability of targeting traditionally “undruggable” protein surfaces [21]. In addition, cyclopeptides exhibit increased metabolic stability in comparison with their linear counterparts.

In particular, a relatively large number of cyclic tetrapeptides reported in the literature have shown interesting bioactivities. However, due to their size, their synthesis can be challenging. As the ring size decreases, peptide cyclization becomes more difficult due to energy constraints [22]. The presence of turn-inducing motifs, such as D-amino acids or *N*-methyl amino acids (*N-*MeAA), are relevant in promoting a preorganized conformation for cyclization. In addition, cyclopeptides containing *N-*MeAA could produce an impact on bioactivity as cell permeability and chemical stability are increased [23].

**Figure 1:**
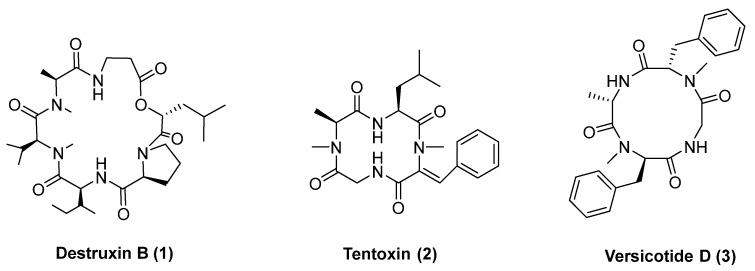
Cyclopeptide natural products with herbicidal activity: destruxin, tentoxin and versicotide D.

Relevant examples of bioactive cyclopeptides with phytotoxic activity are destruxin B (**1**), Figure 1, and tentoxin (**2**). Tentoxin, a cyclic tetrapeptide produced by the fungus *Alternaria tenuis*, causes seedling chlorosis [24]. Although tentoxin has an interesting herbicide activity, studies suggest it can be hepatotoxic [25], and its synthesis is very challenging. In fact, several synthetic routes for obtaining tentoxin were described [26,27,28,29,30], and many of them show low yields.

Our group has recently reported the phytotoxic evaluation of natural products versicotides, among which versicotide D (**3**, Figure 1) shows low cytotoxicity on HepG2 cells [31], high phytotoxicity (74% radicle growth inhibition at 67 μg/mL) and the ability to inhibit cyanobacteria population with a substantial depletion of the concentration of microcystins in the media [32]. Here, we report the synthesis of a library of cyclic tetrapeptides inspired on tentoxin and versicotide D and the evaluation of their phytotoxicity against Ryegrass (*Lolium multiflorum*) seeds. The design of the compounds was focused on small modifications of the peptide backbone, exploring the influence on the bioactivity of L- or D-amino acids and *N-*MeAA.

## 2. Results and Discussion

### 2.1. Synthesis

**Scheme 1:**
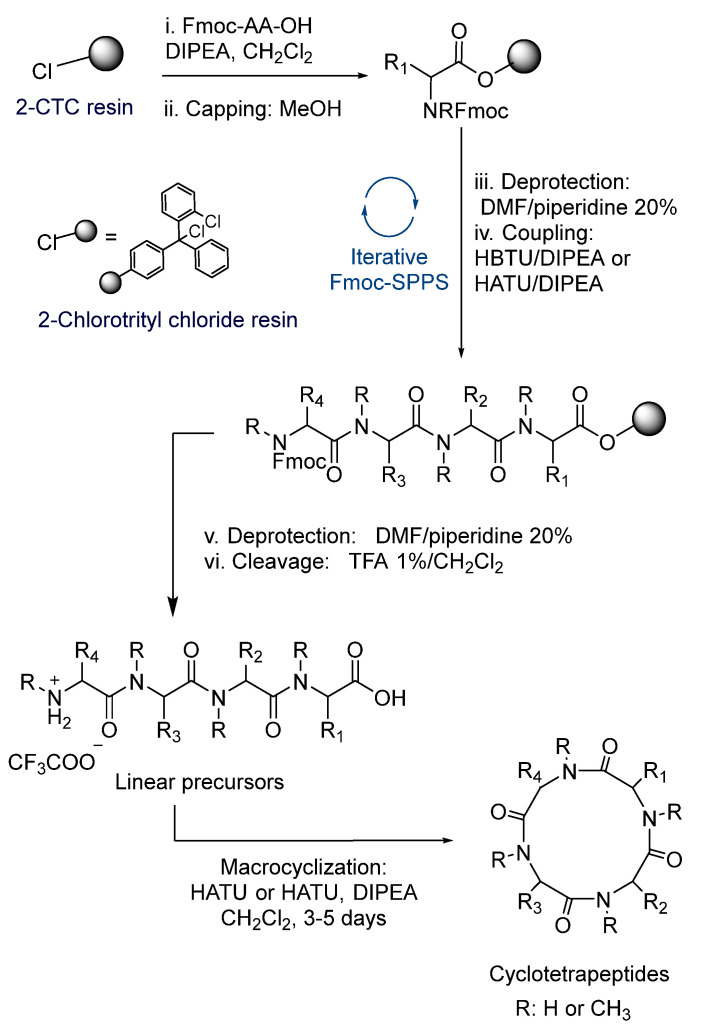
General procedure of the SPPS Fmoc-strategy and solution macrocyclization.

**Figure 2:**
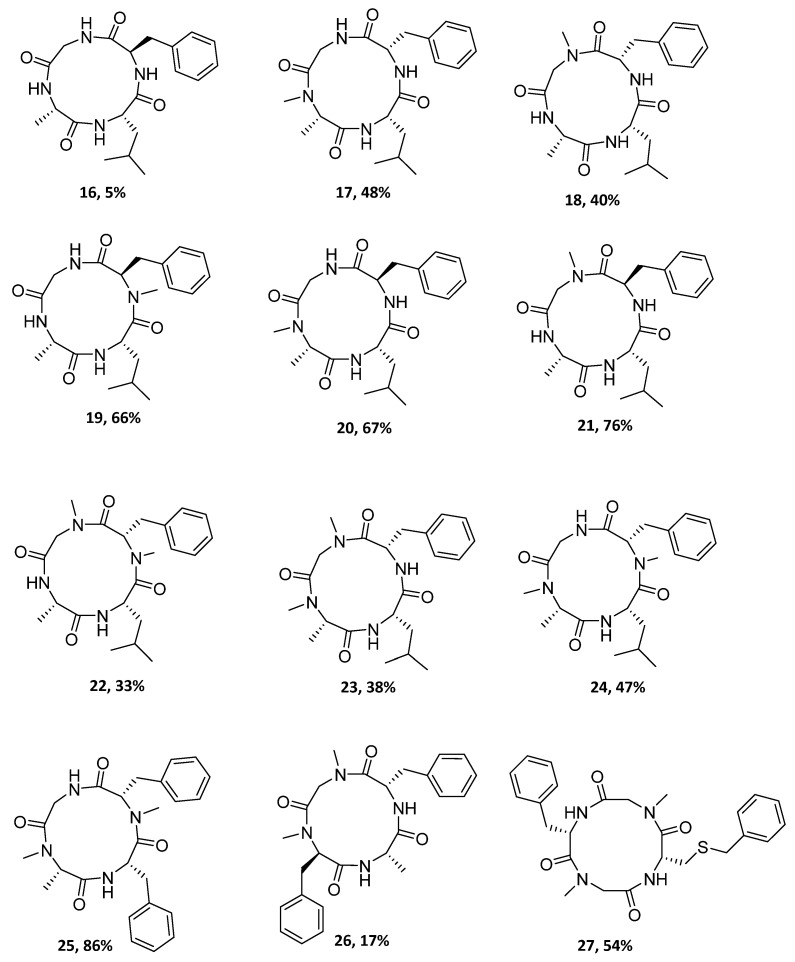
Structures and yields of the synthesized cyclopeptides.

The synthesis of the linear tetrapeptides, **4**–**15**, as shown in Table 1, and the corresponding cyclopeptides **16**–**27**, as shown in Figure 2, were carried out following the procedure shown in Scheme 1. The methodology was based on the Fmoc-strategy of SPPS (solid phase peptide synthesis), employing the 2-chlorotrityl chloride resin (2-CTC resin). To avoid racemization, and thereby the formation of diastereomers during ring closure, we started most peptide sequences with Glycine at the C-terminus, which also minimizes steric hindrance during the macrocyclization process. The yields and purities of the obtained tetrapeptides are shown in Table 1. HBTU was used for coupling to primary amine and HATUwhen coupling to an *N-*MeAA. (Characterization Data of Products Can be founded in Supplementary Material)

In order to maximize the expected outcome of the cyclization reaction, the linear sequences of the peptide precursors were carefully chosen. Gly (compounds **4**–**8**, **11**–**13**) or *N*-MeGly (compounds **9**–**11** and **14**–**15**) were selected as C-terminals to avoid epimerization of this residue and minimize steric hindrance during the cyclization process.

The anchorage of Fmoc-Gly-OH or Fmoc-*N*-MeGly-OH to the resin was achieved with excess of DIPEA in CH_2_Cl_2_. For the elongation of the peptide chain, we carried out successive steps of deprotection employing piperidine and amide bond formation, using the corresponding Fmoc-AA-OH and HBTU (*N*,*N*,*N′*,*N′*-Tetramethyl-*O*-(1*H*-benzotriazol-1-yl)uronium hexafluorophosphate) for coupling to primary amines and HATU for secondary amines ((1-[bis(dimethylamino)methylene]-1*H*-1,2,3-triazolo[4,5-b]pyridinium 3-oxid hexafluorophosphate). The elongation of the peptide chain was monitored by HPLC; in some cases, additional steps of deprotection or coupling were needed. Once the desired linear tetrapeptides were reached, cleavage with a solution of TFA 1% in CH_2_Cl_2_ rendered them as trifluoroacetate salts in very good yields and excellent purities.

Diketopiperazine formation is an important side reaction that can hinder peptide synthesis on solid phase. This usually occurs during the coupling between the second and third amino acid in the sequence. It is well known that the use of 2-CTC resin decreases the formation of diketopiperazine due to the presence of the bulky trityl group [33]. Nevertheless, when the second amino acid is an *N-*MeAA, the formation of diketopiperazine can be promoted. In order to avoid this, short times during Fmoc deprotection of *N-*MeAA were used. This procedure allowed us to obtain compounds 7, 12 and 13 in very good yields. However, low yields were obtained for compounds **6**, **9** and **11**, possibly due to their particular peptide sequences.

Once the linear precursors were obtained as trifluoroacetate salts, we proceeded with the head-to-tail lactamization in solution phase, obtaining the corresponding cyclotetrapetides. It is well known that macrocyclization reaction is a low yielding process. The *E*-geometry of the amide bond prevents the peptides from adopting a ring-like conformation conducive to cyclization [34,35]. In addition, the cyclization yield is very dependent on the peptide sequence and on the ring size. In general, the cyclization of peptides with less than seven amino acids is a difficult process, even though small cyclic peptides containing a β-turn such as a d-amino acid, an *N-*MeAA or proline, a thiazole or oxazole ring, etc., can be prepared in higher yields.

To obtain the desired cyclotetrapeptides, high dilution conditions were used, and cyclization reactions were performed for 1–5 days (Scheme 1). Figure 2 and Table 2 show the twelve cyclotetrapeptides analogues to tentoxin (cyclopeptides **16**–**24**) and versicotide D (**25**–**27**) that were prepared using HBTU/DIPEA in the cases where the N-terminal residue is a primary amine and HATU/DIPEA or Oxyma/DIC when the N-terminal residue was a secondary amine. To obtain compound **17**, we also tried the combination of Oxyma/DIC, instead of HATU or HBTU, but the best results were obtained with HATU. For the cyclization of compound **15**, Oxyma/DIC was used and **27** was obtained in 54% yield.

It is important to highlight that in the case of **17** vs. **20**, and **18** vs. **21**, the change of Phe by D-Phe, respectively, leads to a higher cyclization yield. However, **16** containing a D-Phe, was obtained in the lowest yield (5%). Nevertheless, this yield was not optimized and could be improved by the use of others coupling reagents. Taking into account the number of *N-*MeAA in the peptide precursor, most of the higher yields were for compounds with one *N-*MeAA (**17**–**21**). An interesting result is the case of the compound **25**, which contains two *N-*MeAA and was obtained in very good yield. In fact, this was the highest yield for the cyclopeptides synthesized in this work. In contrast, when Phe was substituted by Leu (**25** vs. **24**, respectively), the yield decreased.

### 2.2. Herbicidal Activity

**Figure 3:**
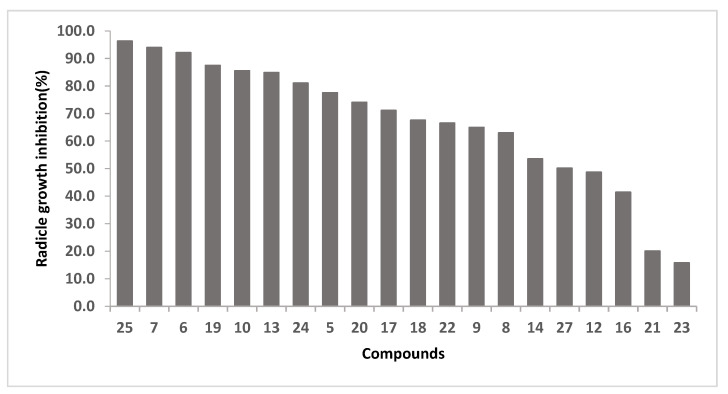
Radicle growth inhibition (%) of compounds at 67 µg/mL, positive control: *S*-metolachlor, 100% inhibition radicle growth at 2.1 µg/mL.

The herbicidal activity of cyclotetrapeptides **16**–**25**, **27** and the linear precursors **4**–**8**, **10**, **12**–**15** were evaluated for their influence on germination, leaf development and radicle length growth of ryegrass seeds at 67 μg/mL using germination in agar methodology [32]. DMSO was used as negative control and the herbicide *S*-metolachlor as positive control. In this trial, the results showed that root length was the only variable for which significant differences were found after a statistical analysis. The results were expressed as % of radicle growth inhibition, calculated as the percentage between the treatment with each compound and radicle length growth for the negative control (DMSO), as shown in Figure 3. Cyclopeptide **26** was tested at 45 μg/mL and showed 67% inhibition.

The most active compounds are the cyclotetrapeptide **25**, and the tetrapeptides **7** and **6**, for which an inhibition of 96%, 94% and 92% at 67 µg/mL was observed, respectively. At this concentration, the cyclization of **13** to obtain **25** leads to an increase in activity. On the contrary, the cyclization of **6** and **10** to render **18** and **22**, respectively, leads to a decrease in herbicidal activity.

By comparison of the herbicidal activity of **16** and **19**, we concluded that the presence of *N-*methyl group is relevant for the bioactivity. Moreover, cyclopeptides **20** and **19,** which differ in the position of *N*-methyl group, present root growth inhibition of 74% and 87%, respectively. On the other hand, **17,** which presents a Phe instead of a D-Phe, seems to be as active as **20**. However, the substitution of Phe in **6** (NH_2_-Ala-Leu-Phe-*N*-MeGly-OH) by D-Phe (**9**) leads to a decrease on the herbicidal activity.

The activity results for cyclotetrapeptides analogues of tentoxin, which contain two *N-*MeAA (**22**–**24**), allowed us to conclude that the position of this type of amino acid is relevant for the bioactivity, whereas **23** is inactive (inhibition 16%), **22** and **24** present 66% and 81% of inhibition, respectively. It is important to note that unlike **24**, **22** and **23** present two contiguous *N-*MeAA**.**

In the case of cyclotetrapeptide **24**, the change of Leu for Phe (compound **25**) leads to an increase on the activity, and for their corresponding linear precursors (**12** and **13**), this fact becomes more noticeable.

The most active compounds, which exhibited inhibition greater than 85% at 67 μg/mL (compounds **6**, **7**, **10**, **13**, **19**, **25,** Figure 3) and cyclopeptides **17**, **20**, **24** and **26**, were selected to assay them at a lower concentration in order to obtain more experimental results for a deep discussion about chemical structure requirements, as shown in Table 3. The linear peptide **6**, did not show inhibition at 23 µM and **10** presented only 15% of root grow inhibition at 11 µM. All the other compounds (**7**, **13**, **17**, **19**, **20**, **24**, **25** and **26**) showed considerable inhibition at concentrations between 4 and 11 µM. The open precursors of the cyclopeptides **19** and **25**, the peptides **7** and **13**, presented similar activity, suggesting that cyclization does not have influence on the bioactivity. However, **26** is a very active compound and its open precursor (**14**) showed lower activity at 67 µM (53%).

At lower concentration (6 μM), cyclopeptides **20** and **17**, which differ on the stereochemistry of Phe, present different bioactivity, 32% and 10% inhibition, respectively.

Compound **24** containing two *N*Me-AA in the (1, 3) relative position in the cycle, presents substantial activity, 62% inhibition at 10 μM. In addition, the open peptide precursor, **12**, is one of the less active of this series (Figure 3).

## 3. Materials and Methods

### 3.1. Synthesis

All reactions were carried out under nitrogen atmosphere with dry, freshly distilled solvents, under anhydrous conditions, unless otherwise stated. All solvents were purified following procedures described in the literature [36]. The cyclopeptides were analyzed using HPLC-DAD-ESI-MS/MS Shimadzu LCMS 8040 equipped with LC-20 AD pump, a DGU solvent degasser solvent, a SPD-M20AD detector, a CTO.20A oven, a Sil-20A injector. The mass spectrometer is connecting by a split 4:1 of flow. The data were processed by the Labsolutions LCMS software. Chromatographic analysis were developed using a Kinetex EVO C18 (100 mm × 4.6 mm, 5 μm solid core particle), using a flow of 1.25 mL/min and 40 °C. Analysis were developed by a gradient solvent system using 0.1% formic acid (mobile phase A) and acetonitrile (mobile phase B). The gradient program was: t = 0’8% B, t = 18’90% B.

Analysis of LC-DAD-MS was recorded by UV absorbance in a 220–360 nm range, and by full scan ESI + ions with a range of 200–1000 uma.

^1^H and ^13^C-NMR spectra were recorded at 25 °C on a Bruker Neo 400 using a BBO z-gradient probe, operating at 400.13 and 100.62 MHz for ^1^H and ^13^C, respectively.

Solid Phase Peptide Synthesis of linear peptides (**4**–**15**):

(I) Resin loading: 500 mg of 2-Chlorotrityl chloride resin (2-CTC) were added to a plastic syringe. The resin was swelled in CH_2_Cl_2_ (3 × 5 min). A solution of first protected amino acid Fmoc-AA-OH (1 eq. for 0.8 mmol/g loading) and DIPEA (3 eq.) in CH_2_Cl_2_ was added and shaken for 10 min. Then, an extra 7.0 eq. of DIPEA were added, and shaking was continued for 50 min. MeOH (0.8 mL/g of resin) was added to the previous mixture and shaken for 10 min. After filtering, the resin was washed with CH_2_Cl_2_ (×3), MeOH (×3), CH_2_Cl_2_ (×3), DMF (×3).
(II) Removal of NHFmoc group: The resin was shaken with piperidine-DMF solution (1:4) (1 × 1 min and 2 × 5 min). In exceptional cases, deprotection step was accomplished by a single treatment with piperidine-DMF solution for 5 min in order to prevent side reactions.
(III) Coupling of subsequent N-Fmoc protected amino acids to primary or secondary amines: After removal of NHFmoc-protecting group, as previously described, the resin was washed with DMF (×3), CH_2_Cl_2_ (×3) and DMF (×3). Then, a solution of Fmoc-AA-OH (3 eq.) and DIPEA (6 eq.) in DMF was added to the resin, followed by a solution of HBTU, for coupling to primary amines, or HATU (2.9 eq.) in DMF, in case of coupling to an *N*-methylamino acid. The mixture was stirred for 60 min. After the coupling was completed, the resin was washed with DMF (×3) and CH_2_Cl_2_ (×3). Successive deprotection and coupling cycles were made with the appropriate amino acids to obtain the desired compound. The coupling was monitored by colorimetric assays; Kaiser test in case of primary amines and Chloranil test for secondary amines. Coupling procedure was repeated in case of positive results.
(IV) Cleavage: The peptide was cleaved from the resin by treatment with 1% TFA in CH_2_Cl_2_ for 2–3 min at room temperature, followed by filtration and collection of the filtrate in MeOH. The treatment was repeated three times and then the resin was washed with CH_2_Cl_2_ (×5) and MeOH (×3). Solvents were removed under vacuum to obtain the crude peptide. LC-MS was used to identify the desired product
(V) General procedure for macrocyclization in solution phase to obtain (**16**–**27**):
  Method I: Macrocyclization reaction was performed in diluted conditions (1–5 mM) using HBTU or HATU (1.5 eq.), DIPEA (3 eq.), 4-DMAP (catalytic) in dried CH_2_Cl_2_ at room temperature during 1–5 days. The reaction mixture was washed with HCl 5%, later with saturated aqueous NaHCO_3_, dried over MgSO_4_, filtered and concentrated in vacuo. The crude was purified by flash chromatography to obtain the pure macrocycle.
  Method II: The trifluoroacetate salt of the corresponding linear peptide was dissolved in dried CH_2_Cl_2_ and diluted to a concentration of 1–5 mM. DIPEA (1 eq.) was added to enable dissolution. EDCI (1.2 eq) and oxyma (1.2 eq.) were added at 0 °C and the reaction mixture was stirred for 10 min. Then, the reaction mixture is allowed to reach room temperature and stirred for 48 h. The reaction mixture was washed with HCl 5%, later with saturated aqueous NaHCO_3_, dried over MgSO_4_, filtered and concentrated in vacuo. The crude was purified by flash chromatography to obtain the pure macrocycle.

*Cyclo-*[Ala-Leu-d-Phe-Gly] (**16**): Solution phase macrocyclization reaction was carried out following general procedure Method I (dilution 5 mM, 5 days), starting from the trifluoroacetate salt of 4: NH_2_-Ala-Leu-d-Phe-Gly-OH (300 mg, 0.58 mmol); HBTU were used as coupling reagent. Purification by flash chromatography, AcOEt:MeOH (1:0.1), rendered the desired macrocycle 16. White solid (5%). Rf = 0.3 (AcOEt). ^1^H NMR (400 MHz, 0.1% MeOD-*d_4_* in CDCl_3_) δ (ppm): 8.01 (d, *J* = 7.2 Hz, 1H), 7.77 (d, *J* = 5.6 Hz, 1H), 7.39–6.99 (m, 5H), 4.48–4.34 (m, 1H), 4.22–3.82 (m, 4H), 3.32 (dd, *J* = 13.9, 3.7 Hz, 1H), 2.96 (dd, *J* = 14.0, 11.2 Hz, 1H), 1.74–1.49 (m, 2H), 1.46 (d, *J* = 7.1 Hz, 3H), 0.86 (d, *J* = 6.5 Hz, 3H), 0.82 (d, *J* = 6.5 Hz, 3H). ^13^C NMR (100 MHz, 0.1% MeOD-*d_4_* in CDCl_3_) multiple conformers were observed, δ (ppm): 175.2, 172.9, 172.4, 172.3, 171.6, 171.5, 137.0, 129.2, 129.0, 128.7, 128.6, 127.0, 56.8, 56.7, 51.8, 50.9, 42.5, 37.0, 36.7, 24.8, 23.0, 21.7, 16.8. ESI-MS m/z calc. for C_40_H_56_N_8_NaO_8_^+^: 799.41 ([2M + Na^+^]^+^), found 799.50. The purity (90.1%) was determined by HPLC, linear gradient, t = 0’ 30% B, t = 15’ 90% B, 25 °C, 220 nm, (rt = 6.79 min). A: H_2_O, 0.1% formic acid, B: MeCN, 0.1% formic acid.

*Cyclo-*[*N*-MeAla-Leu-Phe-Gly] (**17**): Solution phase macrocyclization reaction was carried out following general procedure Method I (dilution 5 mM, 84 h), starting from the trifluoroacetate salt of 5: NH-MeAla-Leu-Phe-Gly-OH (200 mg, 0.37 mmol). HATU was used as coupling reagent. Purification by flash chromatography, AcOEt:MeOH (3:0.2), rendered the desired macrocycle **17**. White solid (48%). Rf = 0.5 (AcOEt:MeOH, 3:0.2). ^1^H NMR (400 MHz, DMSO-*d_6_*), multiple conformers were observed, δ (ppm): 8.16–7.71 (m, 2H), 7.65–7.54 (m, 1H), 7.30–7.13 (m, 5H), 5.00–4.92 (m, 0.2H), 4.84–4.76 (m, 0.1H), 4.67 (q, *J* = 7.4 Hz, 0.5H), 4.62–4.38 (m, 0.8H), 4.41–4.20 (m, 1.4H), 4.20–4.06 (m, 0.8H), 4.05–3.90 (m, 0.6H), 3.92–3.82 (m, 0.3H), 3.74 (dd, *J* = 16.8, 3.6 Hz, 0.5H), 3.14–2.97 (m, 1.4H), 2.93 (s, 1.4H), 2.88–2.70 (m, 1.6H), 2.7–2.6 (m, 0.6H), 1.59–1.32 (m, 3H), 1.33–1.12 (m, 3H), 0.80 (d, *J* = 6.1 Hz, 3H), 0.76 (d, *J* = 5.7 Hz, 3H). ^13^C NMR (100 MHz, DMSO*-d_6_*), multiple conformers were observed, δ (ppm): 172.2, 171.6, 170.7, 169.9, 138.6, 129.7, 129.6, 129.5, 128.6, 128.5, 127.7, 126.7, 55.8, 55.5, 52.7, 41.7, 37.1, 32.5, 25.4, 25.0, 24.7, 24.6, 23.5, 23.2, 22.0, 21.9, 14.1. ESI-MS m/z calc. for C_42_H_60_N_8_NaO_8_: 827.44 (2M + Na^+^]^+^), found 827.55. The purity (90.7%) was determined by HPLC, linear gradient 1.25 mL/min, t = 0′ 30% B, t = 15′ 90% B, 25 °C, 220 nm, (rt = 7.41 min). A: H_2_O, 0.1% formic acid, B: MeCN, 0.1% formic acid.

*Cyclo*-[Ala-Leu-Phe-*N*-MeGly] (**18**): Solution phase macrocyclization reaction was carried out following general procedure Method I (dilution 5 mM, 5 days), starting from the trifluoroacetate salt of 6: NH_2_-Ala-Leu-Phe-NMeGly-OH (207 mg, 0.39 mmol); HBTU was used as coupling reagent. Purification by flash chromatography, AcOEt:MeOH (1:0.1), rendered the desired macrocycle **18**. White solid (40%). Rf = 0.3 (AcOEt). ^1^H NMR (400 MHz, MeOD-*d_4_*), multiple conformers were observed, δ (ppm): 7.43–7.03 (m, 5H), 5.29–5.05 (m, 0.2H), 5.01–4.81 (m, 0.5H), 4.66–4.56 (m, 0.5H), 4.54–4.27 (m, 1.4H), 4.18–4.05 (m, 0.5H), 4.04–3.94 (m, 0.6 H), 3.77–3.66 (m, 0.3 H), 3.35–2.93 (m, 3H), 2.72 (s, 3H), 1.82–1.19 (m, 3H), 1.50 (d, *J* = 7.3 Hz, 3H), 1.03–0.67 (m, 6H). ^13^C NMR (100 MHz, CDCl3), δ (ppm)), multiple conformers were observed: 175.1, 174.2, 173.2, 168.5, 135.3, 129.4, 129.1, 128.9, 128.7, 128.5, 127.5, 127.2, 54.0, 52.0, 50.5, 39.2, 37.5, 37.0, 36.7, 29.7, 24.7, 24.6, 23.0, 22.9, 22.7, 21.9, 21.4, 16.8, 14.1. ESI-MS m/z calc. for C_21_H_31_N_4_O_4_^+^: 403.23 ([M + H]^+^), found 403.05. The purity (97.6%) was determined by HPLC, linear gradient, t = 0′ 30% B, t = 10′ 90% B, 25 °C, 220 nm, (rt = 7.53 min). A: H_2_O, 0.1% formic acid, B: MeCN, 0.1% formic acid.

*Cyclo*-[Ala-Leu-*N*-Me-d-Phe-Gly] (**19**): Solution phase macrocyclization reaction was carried out following general procedure Method I (dilution 5 mM, 5 days), starting from the trifluoroacetate salt of 7: NH_2_-Ala-Leu-*N*-Me-d-Phe-Gly-OH (300 mg, 0.56 mmol); HBTU was used as coupling reagent. Purification by flash chromatography, AcOEt:MeOH (1:0.1) to AcOEt:MeOH (1:0.5), rendered the desired macrocycle **19**. White solid (66%). Rf = 0.4 (AcOEt). ^1^H NMR (400 MHz, DMSO-*d_6_*), multiple conformers were observed, δ (ppm): 8.10–8.01 (m, 1H), 8.01–7.84 (m, 2H), 7.44 (d, *J* = 7.51 Hz., 1H), 7.27–7.09 (m, 5H), 5.51–5.28 (m, 1H), 4.62–4.40 (m, 1H), 4.37–4.18 (m, 1H), 3.98–3.55 (m, 2H), 3.29–3.18 (m, 1H), 2.96–2.80 (m, 4H), 1.25–1.09 (m, 3H), 1.11–0.76 (m, 3H), 0.76–0.57 (m, 6H). ^13^C NMR (100 MHz, DMSO_-_*d*_6_), multiple conformers were observed, δ (ppm): 173.4, 173.1, 173.0, 172.3, 172.2, 172.1, 170.7, 170.4, 170.2, 168.8, 168.6, 168.6, 138.2, 138.2, 138.1, 129.7, 129.3, 129.3, 128.5, 126.7, 58.0, 57.7, 52.2, 48.3, 48.1, 47.7, 47.7, 42.8, 42.6, 41.0, 33.6, 31.9, 31.2, 24.1, 24.1, 23.3, 23.2, 22.5, 22.2, 22.2, 19.1, 18.8, 18.8. ESI-MS m/z calc. for C_42_H_60_N_8_NaO_8_^+^: 827.44 ([2M + Na]^+^) found 827.60. The purity (98.7%) was determined by HPLC, isocratic flow 1 mL/min, MeOH: H_2_O (70:30), 30 °C, 220 nm, (rt = 9.97 min).

*Cyclo*-[*N*-MeAla-Leu-d-Phe-Gly] (**20**): solution phase macrocyclization reaction was carried out following general procedure Method I (dilution 5 mM, 5 days), starting from the trifluoroacetate salt of 8: NH-MeAla-Leu-d-Phe-Gly-OH (300 mg, 0.56 mmol); HATU was used as coupling reagent. Purification by flash chromatography, AcOEt:MeOH (1:0.1) to AcOEt:MeOH (1:0.5), rendered the desired macrocycle **20**. White solid (67%). Rf = 0.4 (EtOAc). ^1^H NMR (400 MHz, CDCl_3,_ 0.1% MeOD-*d_4_*), multiple conformers were present, δ (ppm): 8.91–8.46 (m, 1H), 8.16–7.83 (m, 1H), 7.60–7.34 (m, 1H), 7.35–7.05 (m, 5H), 7.03–6.71 (m, 1H), 5.28–4.05 (m, 3H), 3.80–3.60 (m, 1H), 3.60–3.36 (m, 1H), 3.29–2.65 (m, 3H), 2.34–2.62 (m, 1H), 1.81–1.11 (m, 6H), 1.08–0.56 (m, 6H). ^13^C NMR (100 MHz, 0.1% MeOD-*d_4_* in CDCl_3_), multiple conformers were present, δ (ppm): 172.7, 172.3, 172.2, 170.6, 170.3, 137.5, 129.7, 129.2, 129.1, 128.5, 128.3, 128.2, 126.8, 126.7, 72.0, 71.2, 61.6, 55.2, 52.6, 52.4, 52.3, 41.2, 39.5, 36.2, 31.7, 29.7, 29.4, 24.7, 22.7, 22.3, 22.2, 22.1, 19.3, 18.5, 14.7, 14.6, 14.1, 13.9, 13.6. ESI-MS m/z calc. for C_42_H_60_N_8_NaO_8_^+^: 827.44 ([2M + Na]^+^), found 827.50. The purity (91.7%) was determined by HPLC, linear gradient flow 1.25 mL/min, t = 0′ 30% B, t = 15′ 90% B, 25 °C, 220 nm, (rt = 7.41 min). A: H_2_O, 0.1% formic acid, B: MeCN, 0.1% formic acid.

*Cyclo* [Ala-Leu-d-Phe-*N*-MeGly] (**21**): Solution phase macrocyclization reaction was carried out following general procedure Method I (dilution 5 mM, 5 days), starting from the trifluoroacetate salt of 9: NH_2_-Ala-Leu-d-Phe-*N*-MeGly-OH (200 mg, 0.37 mmol); HBTU was used as coupling reagent. Purification by flash chromatography, AcOEt, rendered the desired macrocycle **21**. White solid (76%). Rf = 0.3 (AcOEt:MeOH 1:0.1). ^1^H NMR (400 MHz, CDCl_3_), two conformers were observed a:b (1:0.3), δ (ppm): 8.08 (d, *J* = 7.7 Hz, 1H_a_), 7.96 (bs, 1H_b_), 7.78–7.69 (m, 1H_a,b_), 7.37–7.13 (m, 5H_a,b_), 5.48 (d, *J* = 17.2 Hz, 1H_a_), 5.04 (d, *J* = 17.2 Hz, 1H_b_), 4.85 (td, *J* = 9.9, 3.5 Hz, 1H_a_), 4.75 (d, *J* = 10.1, 2.5 Hz, H_b_), 4.60–4.52 (m, 1H_a_), 4.52–4.45 (m, 1H_b_), 4.17–4.09 (m, 1H_a,b_), 3.39–3.31 (m, 1H_b_), 3.21 (d, *J* = 17.2 Hz, 1H_a_), 3.17–3.04 (m, 2H_a,b_), 2.97–2.82 (m, 1H_a,b_), 2.57 (s, 3H_a_), 2.46 (s, 3H_b_), 1.91–1.74 (m, 1H_a_), 1.55 (d, *J* = 7.3 Hz, 3H_a,b_), 1.45–1.19 (m, 2H_a,b_), 0.93–0.80 (m, 6H_a,b_). ^13^C NMR (100 MHz, CDCl_3_), two conformers were observed a:b (1:0.3), δ (ppm): 174.0 (C_a,b_), 173.7 (C_a,b_), 172.0 (C_a,b_), 169.8 (C_a,b_), 135.0 (C_a_), 134.6 (C_b_), 129.0 (C_b_), 128.2 (C_a_), 127.8 (C_a_), 127.4 (C_b_), 126.5 (C_b_), 126.4 (C_a_), 52.4 (C_a_), 52.1 (C_b_), 51.7 (C_b_), 50.8 (C_a_), 48.8 (C_b_), 47.9 (C_a_), 38.8 (C_b_), 38.4 (C_a_), 36.1 (C_b_), 35.6 (C_a_), 35.1 (C_b_), 34.6 (C_a_), 28.7 (C_b_), 23.9 (C_a_), 22.5 (C_b_), 22.3 (C_a_), 20.5 (C_a_), 20.2 (C_b_), 16.1 (C_b_), 16.0 (C_a_). ESI-MS m/z calc. for C_21_H_31_N_4_O_4_^+^: 403.23 ([M + H^+^]^+^), found 403.00. The purity (95.2%) was determined by HPLC, linear gradient, t = 0′ 30% B, t = 9′ 90% B, 25 °C, 220 nm, (rt = 7.26 min). A: H_2_O, 0.1% formic acid, B: MeCN, 0.1% formic acid.

*Cyclo*-[Ala-Leu-*N*-MePhe-*N*-MeGly] (**22**): Solution phase macrocyclization reaction was carried out following general procedure Method I (dilution 5 mM, 3 days), starting from the trifluoroacetate salt of 10: NH_2_-Ala-Leu-*N*-MePhe-*N*-MeGly-OH (200 mg, 0.36 mmol); HBTU was used as coupling reagent. Purification by flash chromatography, AcOEt:MeOH (3:0.1), rendered the desired macrocycle **22.** White solid (33%). Rf = 0.6 (AcOEt:MeOH 3:0.1). ^1^H NMR (400 MHz, CDCl_3_), δ (ppm): 8.51 (bs, 1H), 7.92 (d, *J* = 8.5 Hz, 1H), 7.35–7.14 (m, 5H), 4.66 (dq, *J* = 13.4, 6.7 Hz, 1H), 3.92–3.88 (m, 1H), 3.84 (d, *J* = 18.5 Hz, 1H), 3.70 (d, *J* = 18.5 Hz, 1H), 3.64–3.53 (m, 1H), 3.48–3.34 (m, 2H), 3.06 (s, 3H), 2.40 (s, 3H), 2.00–1.87 (m, 1H), 1.78–1.60 (m, 2H), 1.25 (d, *J* = 6.5 Hz, 3H), 1.05 (d, *J* = 6.7 Hz, 3H), 0.86 (d, *J* = 6.6 Hz, 3H). ^13^C NMR (100 MHz, CDCl_3_), δ (ppm): 174.0, 172.2, 170.1, 167.2, 138.5, 129.7, 128.7, 127.0, 66.71, 54.7, 51.7, 45.7, 40.3, 38.6, 37.8, 37.6, 35.1, 24.5, 23.5, 21.0, 16.5. ESI-MS m/z calc. for C_22_H_33_N_4_O_4_^+^: 417.24 ([M + H^+^]^+^) found 417.65. The purity (92.8%) was determined by HPLC, isocratic flow 1mL/min, MeOH:H_2_O (70:30), 30 °C, 220 nm, (rt = 3.01 min).

*Cyclo*-[*N*-MeAla-Leu-Phe-*N*-MeGly] (**23**): Solution phase macrocyclization reaction was carried out following general procedure Method I (dilution 5 mM, 3 days), starting from the trifluoroacetate salt of 11: *N*-MeAla-Leu-Phe-*N*-MeGly-OH (168 mg, 0.31 mmol); HBTU was used as coupling reagent. Purification by flash chromatography, AcOEt, rendered the desired macrocycle **23**. White solid (38%). Rf = 0.5 (AcOEt). ^1^H NMR (400 MHz, CDCl_3_), multiple conformers were observed, δ (ppm): 7.94 (d, *J* = 9.9 Hz, 0.1 H), 7.66 (d, *J* = 7.7 Hz, 0.1 H), 7.49 (d, *J* = 8.9 Hz, 0.2 H), 7.36–7.12 (m, 5H), 7.04–6.96 (m, 0.5H), 6.89–6.81 (m, 0.5H), 6.29 (d, *J* = 9.1 Hz, 0.1 H), 6.04–5.92 (m, 0.2 H), 5.23–5.13 (m, 0.5 H), 5.12–5.06 (m, 0.2 H), 5.05–4.98 (m, 0.4H), 4.87 (q, *J* = 6.4 Hz, 0.2 H), 4.66–4.41 (m, 1H), 4.40–4.28 (m, 0.5 H), 3.36–2.72 (m, 9H), 2.07–1.97 (m, 0.25H), 1.87–1.80 (m, 0.5H), 1.74–1.67 (m, 0.5H), 1.55–1.47 (m, 1H), 1.46 (d, *J* = 7.2Hz, 1.2 H), 1.37 (d, *J* = 7.2 Hz, 1.8H), 1.32–1.2 (m, 1H), 1.03–0.76 (m, 6H). ^13^C NMR (100 MHz, CDCl_3_), multiple conformers were observed, δ (ppm): 172.7, 172.6, 172.3, 171.8, 171.8, 171.3, 171.3, 170.7, 170.2, 170.0, 168.9, 168.3, 137.5, 137.3, 136.7, 129.6, 129.5, 129.5, 129.1, 128.5, 128.4, 128.3, 126.8, 126.7, 126.5, 56.4, 54.4, 53.8, 53.0, 51.6, 51.3, 51.1, 50.4, 50.4, 49.6, 41.2, 40.1, 39.0, 38.8, 38.7, 38.6, 29.7, 28.9, 25.3, 25.0, 23.3, 23.0, 21.1, 14.8, 14.1, 13.2. ESI-MS m/z calc. for C_22_H_33_N_4_O_4_^+^: 417.24 ([M + H^+^]^+^), found 417.55. The purity (95.5%) was determined by HPLC, linear gradient, t = 0′ 30% B, t = 15′90% B, 220nm, (rt = 10.98 min). A: H_2_O, 0.1% formic acid, B: MeCN, 0.1% formic acid.

*Cyclo*-[*N*-MeAla-Leu-*N*-MePhe-Gly] (**24**): Solution phase macrocyclization reaction was carried out following general procedure Method I (dilution 5 mM, 84 h), starting from the trifluoroacetate salt of 12: *N*-MeAla-Leu-*N*-MePhe-Gly-OH (300 mg, 0.51 mmol); HATU was used as coupling reagent. Purification by flash chromatography, AcOEt, rendered the desired macrocycle **24.** Yellow oil (47%). Rf = 0.5 (EtOAc). ^1^H NMR (400 MHz, CDCl_3_) δ (ppm): 7.81 (d, *J* = 9.3 Hz, 1H), 7.40–7.17 (m, 5H), 4.96 (dd, *J* = 15.2, 10.0 Hz, 1H), 4.62 (dd, *J* = 11.3, 3.5 Hz, 2H), 4.27 (q, *J* = 7.0 Hz, 1H), 3.70 (dd, *J* = 13.7, 1.5 Hz, 1H), 3.51 (d, *J* = 15.1 Hz, 1H), 2.96–2.85 (m, 1H), 2.83 (s, 3H), 2.77 (s, 3H), 1.73–1.63 (m, 1H), 1.56 (d, *J* = 7.1 Hz, 3H), 1.45–1.33 (m, 1H), 1.32–1.19 (m, 1H), 0.83 (d, *J* = 6.5 Hz, 3H), 0.77 (d, *J* = 6.6 Hz, 3H). ^13^C NMR (100 MHz, CDCl_3_) δ (ppm): 172.3, 171.8, 170.6, 170.1, 137.1, 129.0, 128.2, 127.1, 63.0, 57.2, 48.4, 44.5, 41.0, 34.3, 30.6, 30.1, 24.6, 22.6, 22.4, 15.6. ESI-MS m/z calc. for C_22_H_33_N_4_O_4_: 417.25 ([M + H]^+^), found 417.30. The purity (95.8%) was determined by HPLC, linear gradient 1.25 mL/min, t = 0′ 30% B, t = 15′ 90% B, 30 °C, 220 nm, (rt = 5.34 min). A: H_2_O, 0.1% formic acid, B: MeCN, 0.1% formic acid.

*Cyclo*-[*N-*MeAla-Phe-*N-*MePhe-Gly] (**25**): Solution phase macrocyclization reaction was carried out following general procedure Method I (dilution 5 mM, 72 h), starting from the trifluoroacetate salt of 13: *N-*MeAla-Phe-*N-*MePhe-Gly-OH (220 mg, 0.38 mmol); HATU was used as coupling reagent. Purification by flash chromatography rendered the desired macrocycle **25**. White solid (86%). Rf = 0.35 (AcOEt). ^1^H NMR (400 MHz, CDCl_3_) δ 1.20 (d, *J* = 7.0 Hz, 3H), 2,50 (s, 3H), 2.77 (s, 3H), 3.12 (d, *J* = 7.6 Hz, 2H), 3.34–3.42 (m, 1H), 3.46 (dd, *J* = 16.5, 1.9 Hz, 1H), 3.56 (dd, *J* = 14.0, 4.5 Hz, 1H), 3.77 (dd, *J* = 10.6, 4.1 Hz, 1H), 4.47 (dd, *J* = 16.6, 6.8 Hz, 1H), 5.13 (q, *J* = 7.68 Hz, 1H), 5.46 (q, *J* = 6.9 Hz, 1H), 6.88 (d, *J* = 6.8 Hz, 1H), 7.00- 7.11 (m, 2H), 7.12–7.24 (m, 3H), 7.28–7.32 (m, 2H), 7.32–7.43 (m, 3H), 7.58 (d, *J* = 9.4 Hz, 1H). ^13^C NMR (100 MHz, CDCl_3_) δ 13.6, 29.1, 33.8, 37.9, 39.2, 41.0, 50.6, 51.4, 68.6, 126.7, 126.8, 128.6, 128.7, 129.2, 129.9, 136.8, 138.7, 169.6, 169.6, 170.2, 171.4. ESI-MS *m/z* calc. for C_25_H_31_N_4_O_4_ 450.23 ([M + H]^+^), found 450.85. The purity (95.6%) was determined by HPLC, linear gradient 1.25 mL/min, t = 0′30% B, t = 15′ 90% B, 30 °C, 220 nm, (rt = 11.25 min). A: H_2_O, 0.1% formic acid, B: MeCN, 0.1% formic acid.

*Cyclo*-[*N*-Me-d-Phe-Ala-Phe-*N-*MeGly] (**26**): Solution phase macrocyclization reaction was carried out following general procedure Method I (dilution 5 mM, 24 h), starting from the trifluoroacetate salt of 14: *N*-Me-d-Phe-Ala-Phe-*N*-MeGly-OH (40 mg, 0.07 mmol); HATU was used as coupling reagent. Purification by flash chromatography rendered the desired macrocycle **26**. White solid (17%). Rf = 0.4 (AcOEt:EP, 3:2). ^1^H NMR (400 MHz, CDCl_3_) δ 1.14 (d, *J* = 5.8 Hz, 3H), 2,98 (s, 3H), 2.95–3.08 (m, 3H), 3.10 (s, 3H), 3.23–3.37 (m, 2H), 4.39–4.60 (m, 3H), 5.41 (d, *J* = 14.5 Hz, 1H), 7.16–7.25 (m, 5H), 7.27–7.31 (m, 1H), 7.36–7.42 (m, 4H), 8.12 (s, 1H), 9.21 (s, 1H). ^13^C NMR (100 MHz, CDCl_3_) δ 16.6, 29.7, 30.8, 34.3, 36.1, 38.5, 45.3, 51.2, 54.7, 62.0, 127.0, 127.6, 128.32, 128.8, 128.9, 129.0, 129.1, 129.8, 136.1, 137.2, 169.3, 169.5, 172.0, 173.9. ESI-MS *m*/*z* calc. for 451.23 C_25_H_31_N_4_O_4_ ([M + H]^+^) found 451.24. The purity (91.9%) was determined by HPLC, linear gradient 1.25 mL/min, t = 0′ 30% B, t = 15′ 90% B, 30 °C, 220 nm, (rt = 9.03 min). A: H_2_O, 0.1% formic acid, B: MeCN, 0.1% formic acid.

*Cyclo*-[Phe-*N*-MeGly-Cys(Bn)-*N*-MeGly] (**27**): Solution phase macrocyclization reaction was carried out following general procedure Method II (dilution 5 mM, 48 horas), starting from the trifluoroacetate salt of **15**: NH_2_-Phe-*N*-MeGly-Cys(Bn)-*N*-MeGly-OH (196 mg, 0.31mmol). Oxyma/EDCI were used as coupling reagents. Purification by flash chromatography rendered the desired macrocycle **27**. White solid (54%). Rf = 0.35 (AcOEt). ^1^H NMR (400 MHz, CDCl_3_) δ 2.64 (dd, *J* = 13.6, 6.5 Hz, 1H), 2.72–3.28 (m, 9H), 3.46 (d, *J* = 15.2 Hz, 3H), 3.64–3.82 (m, 3H), 4.20 (d, *J* = 14.9 Hz, 1H), 4.46 (d, *J* = 15.1 Hz, 1H), 4.87–5.00 (m, 1H), 5.02–5.19 (m, 1H), 6.97 (d, *J* = 8.2 Hz, 1H), 7.09 (d, *J* = 8.4 Hz, 1H), 7.14–7.24 (m, 4H), 7.27–7.40 (m, 6H). ^13^C NMR (100 MHz, CDCl_3_) δ 32.8, 35.8, 36.1, 36.2, 37.9, 48.0, 50.0, 51.4, 52.1, 126.7, 126.8, 128.1, 128.2, 128.4, 128.5, 128.6, 129.0, 131.6, 131.8, 135.7, 137.5, 167.3, 167.4, 170.6, 171.6. ESI-MS *m/z* calc. for C_50_H_60_N_8_O_8_S_2_Na 987.4 ([2M + Na]^+^), found 987.5. The purity (94.3%) was determined by HPLC, linear gradient 1.25 mL/min, t0′—30% B, t15′—90% B, 30 °C, 220 nm, (rt = 10.18 min). A: H_2_O, 0.1% formic acid, B: MeCN, 0.1% formic acid.

### 3.2. Herbicidal Activity

The experiments to determine the herbicidal activity of the cyclopeptide compounds were carried out on *Lolium multiflorum* (Raygrass) plants. Germination, root length and leaf development were evaluated, compared to a control without herbicide, a negative control (100 μL DMSO in 15 mL agar, used as solvent) and an herbicide control (1/8 of the commercial dose of the herbicide *S*-metolachlor).

Serial experiments were conducted using the Agar germination methodology, where the tested compounds and the respective controls were placed in glass Petri dishes (6 cm diameter) in three replicates per treatment. Ten ryegrass seeds were germinated in a growth chamber (20 °C, day/night temperature). The seeds were previously sterilized by immersing them in 70% alcohol for 10 s. When distributed in the Petri dish on the agar, the seeds were placed in such a way as to ensure that they remained submerged in the solution.

Agar–water solution was prepared at 0.3%, 3 g of agar was placed in 1 liter of deionized water, and the solution was autoclaved at 100 °C for 45 min. Once the agar medium had cooled to approximately 60 °C, the solutions were prepared.

The negative control–DMSO control was prepared by adding 100 μL of DMSO per plate in 15 mL of agar and then the seeds were distributed, as mentioned above. A control without DMSO was also carried out to check that the product was not altering the correct development of the ryegrass seeds. For this test, 15 ml of agar were placed in each Petri dish and, before it solidified, the seeds of the species evaluated were placed on top. The herbicide treatment, positive control–Control *S*-Metolachlor (960 g/L), was carried out for a conversion of 1/8 of the dose of 1 L/ha of commercial product. For this purpose, a stock solution of *S*-Metolachlor was prepared by placing 0.28 mL of the herbicide in a volumetric flask and topping up to 1000 mL. A total of 25 mL of this stock solution were taken, placed in a volumetric flask and brought to 200 mL, thus, generating the 1/8x solution of *S*-Metolachlor. A volume of 3 mL of 1/8x herbicide solution was mixed with 45 mL of the agar solution to bring 16 mL into each Petri dish. This ensured that there was 1 mL of 1/8x *S*-Metolachlor solution per plate: 0.28 μL of herbicide solution. Seeds were arranged in the same way. Using the same method, the agar media corresponding to each plate (15 mL) was mixed with the cyclopeptides dissolved in 100 μL of DMSO.

Germination, root length and leaf development were evaluated 12 days after preparation. The variables of germinated plants and plants with developed leaves of the total number of plants placed to germinate were analyzed by fitting a generalized linear model, since they presented a binomial distribution.

Glinmix procedure of the SAS statistical package. Based on the model and for the comparison of the treatments with the different controls, the contrasts of interest were carried out. The effect of the treatments on the root length variable was studied by comparing means using a Tukey test (*p*-value < 0.05) in INFOSTAT.

## 4. Conclusions

In summary, cyclopeptides and their open precursors, analogues of tentoxin and versicotide D were successfully synthesized by SPPS of their linear precursor and solution-phase macrolactamization. After evaluating the herbicidal activity of cyclopeptides analogues of tentoxin, we can conclude that **19** and **24** are the most active of them showing remarkable phytotoxic activity. Cyclotetrapeptides **19** and **24** present one (*N-*Methyl- D-Phe) and two *N-*MeAA (*N-*Methyl-Ala and *N-*Methyl-Phe), respectively. The cyclopeptide without *N*-MeAA (**16**) is inactive. In two cases, the open precursors (**7** and **13**) are as active as their corresponding cyclopeptide (**19** and **25**). However, many linear peptides are inactive and their cyclization derivatives showed herbicidal activity. An example of these observed results is **24** and its open precursor **12**, which are the most active and one of the less active compounds, respectively. Moreover, the cyclopeptide analogues of versicotide D, **25** and **26**, showed improved activity over the natural product.

All these findings allowed us to conclude that the conformation adopted by the peptides and cyclopeptides would have great influence on the herbicidal activity. As a consequence, the peptide sequence, the amino acid stereochemistry and the presence of *N*-methyl group would play an important role on the phytotoxic activity of this type of compound.

## Figures and Tables

**Table 1:** Linear peptides obtained by Fmoc- SPPS.

Peptide	Compound	Yield (%)	Purity (%) *
Ala-Leu-d-Phe-Gly (**4**)	4	100	ND
*N*-MeAla-Leu-Phe-Gly (**5**)	5	100	93
Ala-Leu-Phe-*N*-MeGly (**6**)	6	60	94
Ala-Leu-*N-*Me-d-Phe-Gly (**7**)	7	100	99
*N*-MeAla-Leu-d-Phe-Gly (**8**)	8	100	96
Ala-Leu-d-Phe-*N-*MeGly (**9**)	9	62	90
Ala-Leu-*N*-MePhe-*N*-MeGly (**10**)	10	100	97
*N*-MeAla-Leu-Phe-*N*-MeGly (**11**)	11	44	94
*N*-MeAla-Leu-*N*-MePhe-Gly (**12**)	12	100	91
*N*-MeAla-Phe-*N*-MePhe-Gly (**13**)	13	97	98
*N*-Me-d-Phe-Ala-Phe-*N*-MeGly (**14**)	14	99	99
Phe-*N*-MeGly-Cys(Bn)-*N*-MeGly (**15**)	15	75	79

* determined by HPLC analysis.

**Table 2:** Peptide precursors, cyclotetrapeptides and macrocyclization yield.

Peptide Precursor	Cyclopeptide	Macrocyclization Yield (%)
Ala-Leu-d-Phe-Gly (**4**)	**16**	5
*N*-MeAla-Leu-Phe-Gly (**5**)	**17**	48
Ala-Leu-Phe-*N*-MeGly (**6**)	**18**	40
Ala-Leu-NMe-d-Phe-Gly (**7**)	**19**	66
*N*-MeAla-Leu-d-Phe-Gly (**8**)	**20**	67
Ala-Leu-d-Phe-*N-*MeGly (**9**)	**21**	76
Ala-Leu-*N*-MePhe-*N*-MeGly (**10**)	**22**	33
*N*-MeAla-Leu-Phe-*N*-MeGly (**11**)	**23**	38
*N*-MeAla-Leu-*N*-MePhe-Gly (**12**)	**24**	47
*N*-MeAla-Phe-*N*-MePhe-Gly (**13**)	**25**	86
*N*-Me-d-Phe-Ala-Phe-*N*-MeGly (**14**)	**26**	17
Phe-*N*-MeGly-Cys(Bn)-*N*-MeGly (**15**)	**27**	54

**Table 3:** Root growth inhibition (%) of the most active compounds at lowest concentration.

Compound	Concentration (µM)	Radicle Growth Inhibition (%)
**6**	23	No inhibition
**7**	11	48
**10**	11	15
**13**	5	38
**17**	6	10
**19**	9	53
**20**	6	32
**24**	10	62
**25**	4	41
**26**	7	45

## Data Availability

Not applicable.

## Supplementary Materials

The following supporting information can be downloaded at: https://www.mdpi.com/article/10.3390/molecules27217350/s1, 1. General Experimental information; 2. Solid Phase Peptide Synthesis and Solution Phase Macrocyclization; 3. Characterization Data of Products; 4. NMR Spectra, ESI-MS and chromatographic data; 5. Procedure for evaluation of phytotoxic activity.

## References

[1] Sharma A., Kumar V., Shahzad B., Tanveer M., Sidhu G.P.S., Handa N., Kohli S.K., Yadav P., Bali A.S., Parihar R.D. (2019). Worldwide Pesticide Usage and Its Impacts on Ecosystem. SN Appl. Sci..

[2] Lopes F.M., Sandrini J.Z., Souza M.M. (2018). Toxicity Induced by Glyphosate and Glyphosate-Based Herbicides in the Zebrafish Hepatocyte Cell Line (ZF-L). Ecotoxicol. Environ. Saf..

[3] Peillex C., Pelletier M. (2020). The Impact and Toxicity of Glyphosate and Glyphosate-Based Herbicides on Health and Immunity. J. Immunotoxicol..

[4] Meftaul I.M., Venkateswarlu K., Dharmarajan R., Annamalai P., Asaduzzaman M., Parven A., Megharaj M. (2020). Controversies over Human Health and Ecological Impacts of Glyphosate: Is It to Be Banned in Modern Agriculture?. Environ. Pollut..

[5] Wheeler W.B. (2002). Role of Research and Regulation in 50 Years of Pest Management in Agriculture Prepared for the 50th Anniversary of the Journal of Agricultural and Food Chemistry. J. Agric. Food Chem..

[6] Huisman J., Codd G.A., Paer H.W., Ibelings B.W., Verspagen J.M.H., Visser P.M. (2018). Cyanobacterial blooms. Nat. Rev. Microbiol..

[7] Carmichael W.W. (2001). Health Effects of Toxin-Producing Cyanobacteria: “The CyanoHABs”. Hum. Ecol. Risk Assess. Int. J..

[8] Heap I. (2014). Global Perspective of Herbicide-Resistant Weeds. Pest Manag. Sci..

[9] Peterson M.A., Collavo A., Ovejero R., Shivrain V., Walsh M.J. (2018). The Challenge of Herbicide Resistance around the World: A Current Summary. Pest Manag. Sci..

[10] Green J.M., Owen M.D.K. (2011). Herbicide-Resistant Crops: Utilities and Limitations for Herbicide-Resistant Weed Management. J. Agric. Food Chem..

[11] Davis A.S., Frisvold G.B. (2017). Are Herbicides a Once in a Century Method of Weed Control?. Pest Manag. Sci..

[12] Duke S.O. (2012). Why Have No New Herbicide Modes of Action Appeared in Recent Years?. Pest Manag. Sci..

[13] Peters B., Strek H.J. (2018). Herbicide Discovery in Light of Rapidly Spreading Resistance and Ever-Increasing Regulatory Hurdles. Pest Manag. Sci..

[14] Seiber J.N., Coats J., Duke S.O., Gross A.D. (2014). Biopesticides: State of the Art and Future Opportunities. J. Agric. Food Chem..

[15] Vurro M., Boari A. (2006). Natural Compounds for Novel Strategies of Parasitic Plant Management. Natural Products for Pest Management.

[16] Evidente A. (2006). Chemical and Biological Characterization of Toxins Produced by Weed Pathogenic Fungi as Potential Natural Herbicides. Natural Products for Pest Management.

[17] Hasan M., Ahmad-Hamdani M.S., Rosli A.M., Hamdan H. (2021). Bioherbicides: An Eco-Friendly Tool for Sustainable Weed Management. Plants.

[18] Vinogradov A.A., Yin Y., Suga H. (2019). Macrocyclic Peptides as Drug Candidates: Recent Progress and Remaining Challenges. J. Am. Chem. Soc..

[19] Staderini M., Megia-Fernandez A., Dhaliwal K., Bradley M. (2018). Peptides for Optical Medical Imaging and Steps towards Therapy. Bioorg. Med. Chem..

[20] Brea R.J., Reiriz C., Granja J.R. (2010). Towards Functional Bionanomaterials Based on Self-Assembling Cyclic Peptide Nanotubes. Chem. Soc. Rev..

[21] Dougherty P.G., Sahni A., Pei D. (2019). Understanding Cell Penetration of Cyclic Peptides. Chem. Rev..

[22] Sarojini V., Cameron A.J., Varnava K.G., Denny W.A., Sanjayan G. (2019). Cyclic Tetrapeptides from Nature and Design: A Review of Synthetic Methodologies, Structure, and Function. Chem. Rev..

[23] Martì-Centelles V., Pandey M.D., Burguete M.I., Luis S.V. (2015). Macrocyclization Reactions: The Importance of Conformational, Configurational, and Template-Induced Preorganization. Chem. Rev..

[24] Lax A.R., Shepherd H.S., Edwards J.V. (1988). Tentoxin, A Chlorosis-Inducing Toxin from Alternaria as a Potential Herbicide. Weed Technol..

[25] Hessel-Pras S., Kieshauer J., Roenn G., Luckert C., Braeuning A., Lampen A. (2019). In Vitro Characterization of Hepatic Toxicity of Alternaria Toxins. Mycotoxin Res..

[26] Rich D.H., Mathiaparanam P. (1974). Synthesis of the Cyclic Tetrapeptide Tentoxin. Effect of an N-Methyldehydrophenylalanyl Residue on Conformation of Linear Tetrapeptides. Tetrahedron Lett.

[27] Jiménez J.C., Chavarría B., López-Macià À., Royo M., Giralt E., Albericio F. (2003). Tentoxin as a Scaffold for Drug Discovery. Total Solid-Phase Synthesis of Tentoxin and a Library of Analogues. Org. Lett..

[28] Loiseau N., Cavelier F., Noel J.-P., Gomis J.-M. (2002). High Yield Synthesis of Tentoxin, a Cyclic Tetrapeptide. J. Pept. Sci..

[29] Cavelier F., Verducci J. (1995). New Synthesis of the Cyclic Tetrapeptide Tentoxin Employing an Azlactone as Key Intermediate. Tetrahedron Lett..

[30] Edwards J.V., Lax A.R., Lillehoj E.B., Boudreaux G.J. (1986). New Synthesis and Biological Activity of the Cyclic Tetrapeptides Tentoxin and [Pro 1] Tentoxin. Int. J. Pept. Protein Res.

[31] Posada L., Serra G. (2019). First Total Synthesis of Versicotide D and Analogs. Tetrahedron Lett..

[32] Posada L., Rey L., Villalba J., Colombo S., Aubriot L., Badagian N., Brena B., Serra G. (2022). Cyclopeptides Natural Products as Herbicides and Inhibitors of Cyanobacteria: Synthesis of Versicotides E and F. Chem. Sel..

[33] Barlos K., Gatos D., Schäfer W. (1991). Synthesis of prothymosin a (ProTα)—A protein consisting of 109 amino acid residues. Angew. Chem. Int. Ed. Engl..

[34] Schmidt U., Langner J. (1997). Cyclotetrapeptides and cyclopentapeptides: Occurrence and synthesis. J. Pep. Res..

[35] White C., Yudin A. (2011). Contemporary strategies for peptide macrocyclization. Nat. Chem..

[36] Perrin D.D., Armarego W.L.F. (1988). Purification of Laboratory Chemicals.

