# Supplementary material for: Synthesis of Cyclotetrapeptides Analogues to Natural Products as Herbicides
Source: Molecules. 2022 Oct 29;27(21):7350. doi: 10.3390/molecules27217350 (PMC9656801; doi:10.3390/molecules27217350)
Supplement: Supplementary file 1 [file molecules-27-07350-s001.zip › molecules-1979526-supplementary.pdf]

Supporting information

Table of contents

1. General Experimental information……………………………………………………………..…….…....S2
2. Solid Phase Peptide Synthesis and Solution Phase Macrocyclization …………………………………………S3
    2.1. Resin loading……………………………………………………………………………………………S3
    2.2. Removal of NHFmoc-protecting group…………………………………………………….…..…S3
    2.3. Coupling of subsequent N-Fmoc-protected amino acids to free primary or secondary amines...….…..…S3
    2.4. Cleavage………………………………………………………………………………….……..…S3
    2.5 Solution phase macrocyclization..………………………………………………………………...…S3
3. Characterization Data of Products………………………………………………….........…..S4-8
4. NMR Spectra, ESI-MS and chromatographic data……………………………………………..……S9-45
5. Procedure for evaluation of phytotoxic activity…………………………………………………….…S46

1. General experimental information

All reactions were carried out under nitrogen atmosphere with dry, freshly distilled solvents under anhydrous conditions unless otherwise stated. All solvents were purified following procedures described in the literature. [1]

The cyclopeptides were analyzed using HPLC-DAD-ESI-MS/MS Shimadzu LCMS 8040 equipped with an LC-20 AD pump, a DGU solvent degasser solvent, an SPD-M20AD detector, CTO.20A oven, and a Sil-20A injector. The mass spectrometer is connecting by a split 4:1 of flow. The data were processed by the Labsolutions LCMS software. Chromatographic analyses were developed using a Kinetex EVO C18 (100 x4.6 mm, 5μm solid core particle), using a flow of 1.25 mL/min and 40°C. Analyses were developed by a gradient solvent system using 0.1% formic acid (mobile phase A) and acetonitrile (mobile phase B).

Analyses of LC-DAD-MS were recorded by UV absorbance in a 220-360 nm range, and by full scan ESI + ions with a range of 200-1000 uma.

1H and 13C-NMR spectra were recorded at 25°C on a Bruker Neo 400 using a BBO z-gradient probe operating at 400.13 and 100.62 MHz for 1H and 13C, respectively. All chemical shifts were related to TMS as internal reference.

All yields refer to chromatographically and spectroscopically (^{1}H-NMR and ^{13}C-NMR) pure products.

All solid phase reactions were monitored by colorimetric tests (Kaiser or Chloranil). 2-Chlorotrityl chloride resin (2-CTC, 100-200 mesh, 1.1 mEq/g) was acquired from CHEM-IMPEX INT'L INC.

[1] Perrin, D. D. ; Armarego, W. L. F. "Purification of Laboratory Chemicals", 3th Ed. Pergamon Press, Oxford, 1988.

2. Solid Phase Peptide Synthesis and Solution Phase Macrocyclization

2.1. Resin loading

2-Chlorotrityl chloride (2-CTC) resin measuring 500 mg was added to a syringe peptide-synthesis vessel. The resin swelled in CH_2Cl_2 (3 x 5 min).

A solution of first protected amino acid Fmoc-AA-OH (1 eq. for 0.8 mmol/g loading) and DIPEA (3 eq.) in CH_2Cl_2 was added and the resin was shaken 10 minutes. Then, an extra 7.0 eq. of DIPEA was added and shaking continued for 50 min. MeOH (0.8 mL/ g of resin) was added to the previous mixture in order to capture unreacted functional groups on the resin, and shaken for 10 min. After filtering, the resin was washed with CH_2Cl_2 (x3), MeOH (x3), CH_2Cl_2 (x3), and DMF (x3).

2.2. Removal of NHFmoc group

The resin was washed with DMF (x3), and the Fmoc-protecting group was removed by treating the resin with piperidine-DMF solution (1:4) for 1, 5, and 5 minutes successively. In exceptional cases, the deprotection step was accomplished by a single treatment with piperidine-DMF solution for 5 minutes in order to prevent side reactions.

2.3. Coupling of subsequent N-Fmoc protected amino acids to primary or secondary amines

After the removal of the NHFmoc protecting group as previously described, the resin was washed with DMF (x3), CH_2Cl_2 (x3), and DMF (x3). A solution of Fmoc-AA-OH (3 eq.) and DIPEA (6 eq.) in DMF was added to the resin, followed by a solution of HBTU, for coupling to primary amines or HATU (2.9 eq.) in DMF in the case of coupling to an N-methylated amino acid. The mixture was stirred for 60 min. After the coupling was completed, the resin was washed with DMF (×3) and CH_2Cl_2 (x3). Deprotection and coupling cycles were repeated with the appropriate amino acids to provide the desired compound. The completion of the coupling was monitored by colorimetric assays; the Kaiser test was used in the case of primary amines and the Chloranil test was used for secondary amines. The coupling procedure was repeated in the case of positive results.

2.4. Cleavage

Unless otherwise stated, the peptide was cleaved from the resin by treatment with 1% TFA in CH_2Cl_2 for 2-3 minutes at room temperature followed by filtration and collection of the filtrate in MeOH. The treatment was repeated three times and then the resin washed with CH_2Cl_2 (x5) and MeOH (x3). Solvents were removed in vacuo to obtain the crude peptide. LC-MS was used to identify the desired product.

2.5. General procedure for macrocyclization in solution phase.

2.5.1. Method I

A macrocyclization reaction was performed in diluted conditions (1-5 mM) using HBTU or HATU (1.5 eq.), DIPEA (3 eq.), and 4-DMAP (catalytic) in dried CH_2Cl_2 at room temperature during 1-5 days. The reaction mixture was washed with HCl 5% and then with saturated aqueous $NaHCO_3$, dried over $MgSO_4$, filtered, and concentrated in vacuo. The crude was purified by flash chromatography to obtain the pure macrocycle.

2.5.2. Method II

The trifluoroacetate salt of the corresponding linear peptide was dissolved in dried CH_2Cl_2 and diluted to a concentration of 1-5 mM. DIPEA (1eq.) was added to enable dissolution. EDCI (1.2 eq) and oxyma (1.2 eq.) were added at 0ºC and the reaction mixture was stirred for 10 minutes. Then, the reaction mixture is allowed to reach room temperature and stirred for 48 hours. The reaction mixture was washed with HCl 5% and then with saturated aqueous $NaHCO_3$, dried over $MgSO_4$, filtered, and concentrated in vacuo. The crude was purified by flash chromatography to obtain the pure macrocycle.

3. Characterization Data of Products

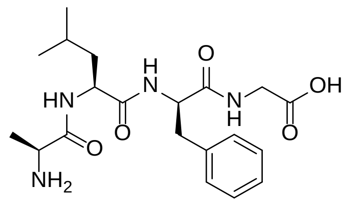

NH_2-Ala-Leu-D-Phe-Gly-OH (4)

The trifluoroacetate salt of NH_2-Ala-Leu-D-Phe-Gly-OH **(4)** was obtained following the general SPPS procedure. Trifluoroacetate salt measuring 590 mg (1.13 mmol) was obtained as a white solid (100%). **^{1}H NMR (400 MHz, D_2O)** δ (ppm): 7.41 - 7.35 (m,2H), 7.34 - 7.28 (m, 3H), 4 83 - 4.80 (m, 1H), 4.21 (dd, *J* = 8.5, 6.2 Hz, 1H), 4.10 – 3.97 (m, 3H), 3.40 (dd, *J* = 14.2, 4.7 Hz, 1H), 2.91 (dd, *J* = 14.2, 11.1 Hz, 1H), 1.46 (d, *J* = 7.1 Hz, 3H), 1.27 (dt, *J* = 16.4, 8.2 Hz, 1H), 1.14 – 1.05 (m, 2H), 0.79 – 0.69 (m, 6H). **^{13}C NMR (100 MHz, D_2O)** δ (ppm): 174.5, 173.5, 173.2, 170.7, 136.5, 129.0, 128.8, 127.2, 54.4, 52.7, 48.7, 41.3, 39.4, 36.8, 23.8, 21.6, 21.1, 16.5. **ESI-MS** m/z calc. for $C_{20}H_{31}N_4O_5$ 407.22([M+H]$^+$), found 407.25.

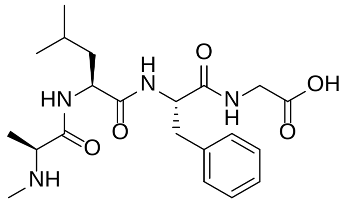

NH-MeAla-Leu-Phe-Gly-OH (5)

The trifluoroacetate salt of NHMe-Ala-Leu-Phe-Gly-OH **(5)** was obtained following the general SPPS procedure. Trifluoroacetate salt measuring 310 mg (0.58 mmol) was obtained as a white solid (100%). **^{1}H NMR (400 MHz, D_2O)** δ (ppm): 7.32 – 7.17 (m, 5H), 4.61 (dd, *J*= 8.7, 6.2 Hz, 1H), 4.30 - 4.24 (m, 1H), 3.89 – 3.80 (m, 3H), 3.10 (dd, J= 13.8, 6.2 Hz, 1H), 2.99 - 2.90 (m, 1H), 2.59 (s, 3H), 1.51 – 1.40 (m, 3H), 1.38 (d, *J* = 7.3 Hz, 3H), 0.82 (d, *J* = 5.8 Hz, 3H), 0.77 (d, *J* = 6.1 Hz, 3H). **^{13}C NMR (100 MHz, D_2O)** δ (ppm): 173.6, 173.2, 173.0, 169.5, 136.3, 129.2, 128.7, 127.1, 57.0, 54.5, 52.5, 41.3, 39.7, 37.1, 31.0, 24.2, 21.9, 20.7, 15.4. **ESI-MS** m/z calc. for $C_{21}H_{33}N_4O_5$: 421.24 ([M+H]$^+$), found 421.35.

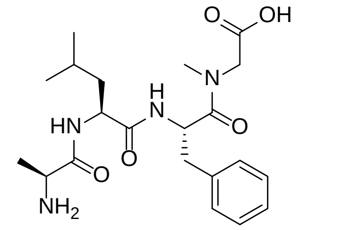

NH_2-Ala-Leu-Phe-NMeGly-OH (6)

The trifluoroacetate salt of NH_2-Ala-Leu-Phe-NMeGly-OH **(6)** was obtained following the general SPPS procedure. Trifluoroacetate salt measuring 235 mg (0.44 mmol) was obtained as a white solid (60%). **^{1}H NMR (400 MHz, D_2O), two conformers were present, a:b (1:0.4)** δ (ppm): 7.41 – 7.22 (m, $5H_{a,b}$), 5.15 (dd, *J* = 8.0, 6.4 Hz, $1H_a$), 4.96 (dd, *J* = 13.9, 7.4 Hz, $1H_b$), 4.28 – 4.20 (m, $1H_{a,b}$), 4.19 - 4.01 (m, 3H), 3.14 (dd, *J* = 13.7, 6.3 Hz, 0.9H), 3.07 (s, 2.2H), 3.09 - 3.03 (m, 0.1H), 3.02 – 2.90 (m, 1H), 2.93 (s, 0.8H), 1.60 – 1.44 (m, 3H), 1.47 (d, *J* = 7.1 Hz, 3H), 0.91 (d, *J* = 5.9 Hz, 3H), 0.86 (d, *J* = 5.9 Hz, 3H). **^{13}C NMR (100 MHz, D_2O), two conformers were present, a:b (1:0.4)** δ (ppm): 173.4 (C_a), 173.2 (C_b), 172.9 (C_b), 172.8 (C_b), 172.8 (C_a), 172.7 (C_a), 170.5 (C_a), 170.4 (C_b), 136.1 (C_b), 136.0 (C_a), 129.4 (C_a), 129.3 (C_b), 128.7 (C_b), 128.6 (C_a), 127.1 ($C_{a,b}$), 52.5 (C_a), 52.4 (C_b), 50.6 (C_a), 50.5 (C_b), 50.3 (C_b), 48.8 (C_a), 39.9 ($C_{a,b}$), 36.9 (C_b), 36.5 (C_a), 35.2 (C_b), 24.2 ($C_{a,b}$), 21.8 ($C_{a,b}$), 20.8 ($C_{a,b}$), 16.6 ($C_{a,b}$). **ESI-MS** m/z calc. for $C_{21}H_{33}N_4O_5$: 421.24([M+H]$^+$), found 421.30.

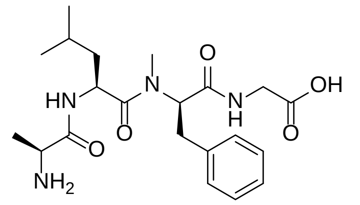

NH_2-Ala-Leu-NMe-D-Phe-Gly-OH (7)

The trifluoroacetate salt of NH_2-Ala-Leu-NMe-D-Phe-Gly-OH **(7)** was obtained following the general SPPS procedure. Trifluoroacetate salt measuring 470 mg (0.88 mmol) was obtained as a white solid (100%). **^{1}H NMR (400 MHz, D_2O)** δ (ppm): 7.39 – 7.33 (m, 2H), 7.33 - 7.27 (m, 3H), 5.60 (dd, *J* = 12.2, 4.8 Hz, 1H), 4.58 (dd, *J* = 10.3, 4.0 Hz, 1H), 4.13 – 3.97 (m, 3H), 3.43 (dd, *J* = 14.5, 4.7 Hz, 1H), 3.10 – 2.97 (m, 4H), 1.49 (d, *J* = 7.1 Hz, 3 H), 1.24 - 1.12 (m, 1H), 0.99 - 0.87 (m, 1H), 0.73 (d, *J* = 6.4 Hz, 6H), 0.56 - 0.45 (m, 1H). **^{13}C NMR (100 MHz, D_2O)** δ (ppm): 175.3, 173.2, 172.4, 170.6, 136.3, 129.0, 128.8, 127.3, 58.4, 49.0, 48.7, 41.2, 38.5, 33.5, 31.5, 24.0, 22.2, 20.4, 16.5. **ESI-MS** m/z calc. for $C_{21}H_{33}N_4O_5$: 421.24 ($[M+H]^+$), found 421.35.

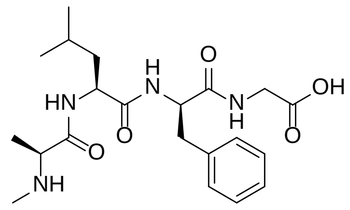

NH-MeAla-Leu-D-Phe-Gly-OH (8)

The trifluoroacetate salt of NH-MeAla-Leu-D-Phe-Gly-OH **(8)** was obtained following the general SPPS procedure. Trifluoroacetate salt measuring 567 mg (1.06 mmol) was obtained as a white solid (100%). **^{1}H NMR (400 MHz, D_2O)** δ (ppm): 7.43-7.23 (m, 5H), 4.85 - 4.76 (m, 1H), 4.28 - 4.2 (m, 1H), 4.10 - 3.97 (m, 2H), 3.90 (q, *J* = 7.0 Hz, 1H), 3.39 (dd, *J* = 14.2, 4.8 Hz, 1H), 2.90 (dd, *J* = 14.2, 11.1 Hz, 1H), 2.66 (s, 3H), 1.46 (d, *J* = 7.0 Hz, 3H), 1.34 – 1.21 (m, 1H), 1.15 - 1.03 (m, 2H), 0.78-0.72 (m, 6H). **^{13}C NMR (100 MHz, D_2O)** δ (ppm): 174.3, 173.5, 173.0, 169.8, 136.5, 129.0, 128.8, 127.2, 56.9, 54.4, 52.7, 41.1, 39.4, 36.8, 31.0, 23.9, 21.7, 20.9, 15.3. **ESI-MS** m/z calc. for $C_{21}H_{33}N_4O_5$: 421.24 ($[M+H]^+$), found 421.35.

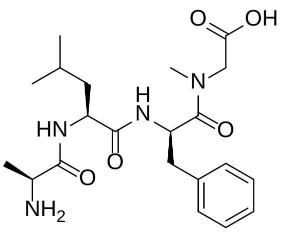

NH_2-Ala-Leu-D-Phe-NMeGly-OH (9)

The trifluoroacetate salt of NH_2-Ala-Leu-D-Phe-NMeGly-OH **(9)** was obtained following the general SPPS procedure. Trifluoroacetate salt measuring 240 mg (0.45 mmol) was obtained as a white solid (62%). **^{1}H NMR (400 MHz, D_2O), two conformers were present, a:b (1:0,3),** δ (ppm): 7.38 – 7.10 (m, $5H_{a,b}$), 5.11 (dd, *J* = 9.6, 5.3 Hz, 1 H_a), 4.91 (dd, *J* = 9.4, 5.3 Hz, $1H_b$), 4.30 (d, *J* = 18.9 Hz, $1H_b$), 4.23 - 4.14 (m, $1H_{a,b}$), 4.11 (d, *J*=17.6 Hz, $1H_a$), 4.01 (d, *J* = 17.6 Hz, $1H_a$), 3.95 (q, *J* = 7.1 Hz, $1H_{a,b}$), 3.16 - 3.01 (m, $4H_a$), 3.01 (dd, *J* = 14.1, 5.3 Hz, $1H_b$), 2.88 (s, $3H_b$), 2.87 – 2.76 (m, $1H_{a,b}$), 1.36 (d, *J* = 7.1 Hz, $3H_{a,b}$), 1.30 – 1.18 (m, $2H_{a,b}$), 1.16 – 1.05 (m, $1H_{a,b}$), 0.74 (d, *J* = 6.2 Hz, $3H_{a,b}$), 0.71 (d, *J* = 6.3 Hz, $3H_{a,b}$). **^{13}C NMR (100 MHz, D_2O), conformers were present, a:b (1:0,3)** δ (ppm): 173.5 (C_a), 173.3 (C_b), 173.2 (C_b), 173.0 (C_a), 173.0 (C_b), 172.9(C_a), 170.5 (C_a), 170.4 (C_b), 136.1, 129.3, 128.7, 127.2, 52.2 (C_a), 52.1 (C_b), 51.8 (C_b), 50.7 (C_a), 50.6 (C_b), 50.5 (C_a), 48.7 (C_a), 39.7 (C_a), 37.1

(C_b), 36.6 (C_a), 35.4 (C_b), 24.1 ($C_{a,b}$), 21.9 ($C_{a,b}$), 20.7 ($C_{a,b}$), 16.5 ($C_{a,b}$). **ESI-MS** m/z calc. for $C_{21}H_{33}N_4O_5$: 421.24 ($[M+H]^+$), found 421.30.

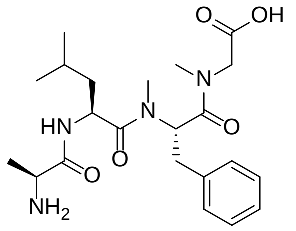

NH_2-Ala-Leu-NMePhe-NMeGly-OH (10)

The trifluoroacetate salt of NH_2-Ala-Leu-*N*-MePhe-*N*-MeGly-OH **(10)** was obtained following the general SPPS procedure. Trifluoroacetate salt measuring 275 mg (0.50 mmol) was obtained as a white solid (100%). **^{1}H NMR (400 MHz, D_2O), two conformers are present a:b (1:0.4)** δ (ppm): 7.28 – 7.11 (m, 5$H_{a,b}$), 5.58 (dd, *J* = 9.7, 5.5 Hz, 1H_a), 5.50 (dd, *J* = 9.3, 5.8 Hz, 1H_b), 4.73 - 4.67 (m, 1H_a), 4.62 (dd, *J* = 11.4, 2.8 Hz, 1H_b), 4.21 (d, *J* = 18.8 Hz, 1H_b), 4.19 (d, *J* = 17.5 Hz, 1H_a), 4.00 – 3.85 (m, 2$H_{a,b}$), 3.01 (s, 3H_a), 3.07 – 2.91 (m, 2$H_{a,b}$), 2.96 (s, 3H_b), 2.89 (s, 3H_a), 2.88 (s, 3H_b), 1.58 – 1.40 (m, 2$H_{a.b}$), 1.40 – 1.2 (m, 1$H_{a,b}$), 1.30 (d, *J* = 7.2 Hz, 3H_a), 1.29 - 1,26 (m, 3H_b), 0.86 (d, *J*=6.4 Hz, 6$H_{a.b}$). **^{13}C NMR (100 MHz, D_2O), two conformers are present a:b (1:0.4)** δ (ppm): 173.0 (C_a), 172.8 (C_b), 172.7 (C_a), 172.4 (C_b), 171.8 (C_a), 171.5 (C_b), 169.7 (C_a), 169.4 (C_b), 136.8 (C_a), 136.6 (C_b), 129.2 (C_a), 129.1 (C_b), 128.5 ($C_{a,b}$), 126.8 (C_b), 126.8 (C_a), 55.4 (C_a), 54.9 (C_b), 50.9 (C_b), 50.4 (C_a), 48.7 ($C_{a,b}$), 48.6 (C_a), 48.4 (C_b), 39.4 (C_a), 39.2 (C_b), 36.2 (C_a), 35.1 (C_b), 34.0 (C_b), 33.6 (C_a), 30.7 (C_a), 30.3 (C_b), 24.3 (C_a), 24.3 (C_b), 22.4 (C_b), 22.3 (C_a), 20.2 (C_a), 20.1 (C_b), 16.7 ($C_{a,b}$). **ESI-MS** m/z calc. for $C_{22}H_{35}N_4O_5^+$: 435.26 ($[M+H]^+$), found 435.30.

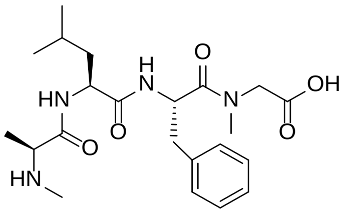

The trifluoroacetate salt of NH-MeAla-Leu-Phe-*N*-MeGly-OH **(11)** was obtained following the general SPPS procedure. Trifluoroacetate salt measuring 192 mg (0.35 mmol) as obtained as a white solid (44%). **^{1}H NMR (400 MHz, D_2O), two conformers are present a:b (1:0.3)** δ (ppm): 7.36 – 7.08 (m, 5$H_{a,b}$), 5.06 (dd, *J*=7.6, 6.8 Hz, 1H_a), 4.90 - 4.84 (m, 1H_b), 4.32 - 4.24 (m, 1$H_{a,b}$), 4.09 (s, 2H_a), 4.00 (s, 2H_b), 3.82 (q, *J* = 7.0 Hz, 1$H_{a,b}$), 3.11 - 2.95 (m, 1$H_{a,b}$), 2.99 (s, 3H_a), 2.96 – 2.84 (m, 1$H_{a,b}$), 2.84 (s, 3H_b), 2.59 (s, 3$H_{a,b}$), 1.52 – 1.40 (m, 3$H_{a,b}$), 1.38 (d, *J* = 7.1 Hz, 3$H_{a,b}$), 0.82 (d, *J* = 5.6 Hz, 3$H_{a,b}$), 0.78 (d, *J* = 5.6 Hz, 3$H_{a,b}$). **^{13}C NMR (100 MHz, D_2O)**, **two conformers are present a:b (1:0.3)** δ (ppm): 173.1 (C_a), 173.0 (C_b), 172.9 (C_b), 172.8 (C_a), 172.6 (C_a), 172.6 (C_b), 169.5 (C_a), 169.4 (C_b), 136.1 (C_b), 136.0 (C_a), 129.4 (C_a), 129.3 (C_b), 128.7 (C_b), 128.6 (C_a), 127.1 ($C_{a,b}$), 57.0 ($C_{a,b}$), 52.4 (C_a), 52.3 (C_b), 51.3 (C_b), 50.6 (C_a), 50.5 (C_b), 50.2 (C_a), 39.8 (C_a), 37.4 (C_b), 36.9 (C_a), 36.5 (C_a), 35.2 (C_b), 31.0 ($C_{a,b}$), 24.3 ($C_{a,b}$), 21.9 ($C_{a,b}$), 20.7 ($C_{a,b}$), 15.4 ($C_{a,b}$). **ESI-MS** m/z calc. for $C_{22}H_{35}N_4O_5^+$: 435.26 ($[M+H]^+$), found 435.35.

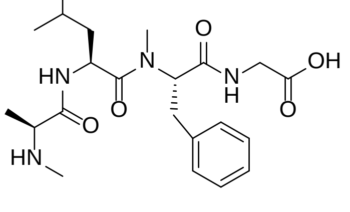

NH-MeAla-Leu-NMePhe-Gly-OH (12)

The trifluoroacetate salt of NH-MeAla-Leu-*N*-MePhe-Gly-OH **(12)** was obtained following the previously described SPPS protocol. Trifluoroacetate salt measuring 486 mg (0.89mmol) was obtained as a white solid (100%). **^{1}H NMR (400 MHz, D_2O), two conformers were observed a:b (1:0.4)** δ (ppm): 7.48 – 7.22 (m, 5H$_{a,b}$), 5.20 (dd, *J* = 10.9, 5.3 Hz, 1H$_a$), 4.98 (dd, *J* = 11.3, 3.5 Hz, 1H$_b$), 4.73 (dd, *J* = 10.8, 3.4 Hz, 1H$_a$), 4.53 (dd, *J* = 11.7, 2.9 Hz, 1H$_b$), 4.12 (d, *J* = 17.8 Hz, 1H$_b$), 4.05 (d, *J*= 17.8 Hz, 1 H$_b$), 4.02 (d, *J* = 17.8 Hz, 1H$_a$), 3.97 (d, *J* = 17.8 Hz, 1H$_a$), 3.93 – 3.83 (m, 1H$_{a,b}$), 3.37 (dd, *J* = 14.2, 3.1 Hz, 1H$_b$), 3.31 (dd, *J* = 14.2, 5.4 Hz, 1H$_a$), 3.19 – 3.09 (m, 1H$_{a,b}$), 3.04 (s, 3H$_a$), 2.98 (s, 3H$_b$), 2.66 (s, 3H$_a$), 2.65 (s, 3H$_b$), 1.63 – 1.49 (m, 1H$_{a,b}$), 1.46 (d, *J* = 7.0 Hz, 3H$_b$), 1.41 (d, *J* = 7.0 Hz, 3H$_a$), 1.39 – 1.29 (m, 1H$_a$), 1.24 – 1.14 (m, 1H$_b$), 0.91 (d, *J* = 6.5 Hz, 3H$_a$), 0.88 (d, *J* = 6.5 Hz, 3H$_a$), 0.69 (d, *J* = 4.9 Hz, 3H$_b$), 0.67 (d, *J* = 4.9 Hz, 3H$_b$). **^{13}C NMR (101 MHz, D_2O), two conformers were observed a:b (1:0.4)** δ (ppm): 174.7 (C$_b$), 173.7 (C$_a$), 173.2 (C$_b$), 173.2 (C$_a$), 172.3 (C$_a$), 171.51 (C$_b$), 169.5 (C$_b$), 168.9 (C$_a$), 163.15, 162.8, 136.9 (C$_b$), 136.6 (C$_a$), 129.5 (C$_b$), 129.4 (C$_b$), 129.0 (C$_{ayb}$), 128.7 (C$_{ayb}$), 127.6 (C$_b$), 126.9 (C$_a$), 62.9 (C$_b$), 60.0 (C$_a$), 57.0 (C$_a$), 56.8 (C$_b$), 48.7 (C$_a$), 47.9 (C$_b$), 41.4 (C$_b$), 41.2 (C$_a$), 39.1 (C$_a$), 37.3 (C$_b$), 33.4 (C$_b$), 33.1 (C$_a$), 33.0 (C$_a$), 31.0(C$_a$), 31.0 (C$_b$), 30.4 (C$_b$), 24.4 (C$_a$), 23.8(C$_b$), 22.3 (C$_a$), 22.2 (C$_b$), 20.2 (C$_a$), 19.5 (C$_b$), 15.5 (C$_a$), 15.2 (C$_b$). **ESI-MS** m/z calc. for $C_{22}H_{35}N_4O_5$: 435.26 ([M+H]$^+$), found 435.40.

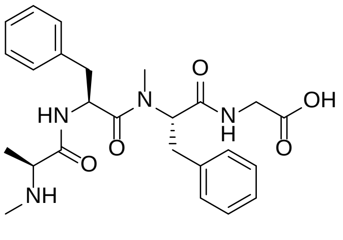

NH-MeAla-Phe-NMePhe-Gly-OH (13)

The trifluoroacetate salt of NHMe-Ala-Phe-*N*-MePhe-Gly-OH (**13**) was obtained following the previously described SPPS protocol. Trifluoroacetate salt measuring 410 mg (0,87mmol) was obtained as a white solid (97%). **^{1}H NMR (400 MHz, D_2O) two conformers were observed a:b (1: 0.46)** δ (ppm): 7.44 (m, 2Hb), 7.23 -7.35 (m, 10H$_a$+6H$_b$), 7.03 (m, 2Hb), 5.35 (dd, *J* = 11.0, 5.3 Hz, 1H$_a$), 5.06 (dd, *J* = 9.3, 6.1 Hz, 1H$_a$), 5.00 (dd, *J* = 10.9, 3.9 Hz, 1H$_b$), 4.64 (dd, *J* = 11.0, 4.3 Hz, 1H$_b$), 3.98 (d, *J* = 4.8 Hz, 2H$_a$), 3.94 (d, *J* = 8.8 Hz, 2H$_b$), 3.62 – 3.73 (m, 1H$_a$ + 1H$_b$), 3.41 (dd, *J* = 14.0, 3.8 Hz, 1H$_b$), 3.29 (dd, *J* = 14.4, 5.2 Hz, 1H$_a$), 3.00 – 3.10 (m, 2Ha +1Hb), 2.99 (s, 3H$_b$), 2.98 (s, 3H$_a$), 2.78 – 2.89 (m, 2H$_a$), 2.56 (dd, *J* = 14.2, 11.1 Hz, 1H$_b$), 2.30 (s, 3H$_a$), 2.13 (s, 3H$_b$), 1.83 (dd, *J* = 14.2, 4.1 Hz, 1H$_b$), 1.35 (d, *J* = 7.0 Hz, 3H$_b$), 1.30 (d, *J* = 7.0 Hz, 3H$_a$). **^{13}C NMR (100 MHz, D_2O)** δ (ppm): 173.1 (C$_b$), 173.0 (C$_a$), 172.8 (C$_b$), 172.5 (C$_a$), 172.0 (C$_a$), 171.3 (C$_b$), 168.4 (C$_b$), 168.3 (C$_a$), 136.6, 136.0, 129.3, 128.9, 128.8, 128.6, 127.3, 126.9, 15.6 (C$_b$), 62.8 (C$_b$), 58.8 (C$_a$), 57.0 (C$_a$), 51.0 (C$_a$), 49.8 (C$_b$), 41.3 (C$_b$), 41.1 (C$_a$), 37.1 (C$_a$), 33.6 (C$_b$), 33.1 (C$_a$), 32.1 (C$_a$), 31.0 (C$_a$), 30.9 (C$_b$), 30.7 (C$_b$), 15.8 (C$_a$). **ESI-MS** *m/z* calc. for $C_{25}H_{33}N_4O_5$ ([M+H]$^+$) 469.24, found 469.20.

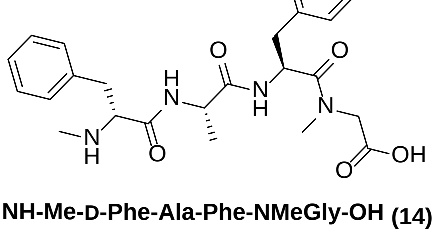

NH-Me-D-Phe-Ala-Phe-NMeGly-OH (14)

The trifluoroacetate salt of NHMe-D-Phe-Ala-Phe-*N*-MeGly-OH **(14)** was obtained following the previously described SPPS protocol. Trifluoroacetate salt measuring 90 mg (0,19mmol) was obtained as a white solid (99%). **^{1}H NMR (400 MHz, $CDCl_3$)** δ (ppm): 0.93 (d, *J*=7.2 Hz, 3H), 2.67 (s, 3H), 2.83 – 2.92 (m, 1H), 2.94 (s, 3H), 2.96 – 3.08 (m, 2H), 3.31 (dd, *J*= 13.1, 5.1 Hz, 1H), 3.39 (d, *J*= 17.2, 1H), 4.05 – 4.17 (m, 1H), 4.24 – 4.31 (m, 1H),

4.50 (d, *J*=17.2 Hz, 1H), 5.13 (q, *J*=7.8 Hz, 1H), 7.09 – 7.25 (m, 7H), 7.28 – 7.35 (m, 3H), 7.89 (d, *J*= 8.6 Hz, 1H), 8.48 (s, 1H).

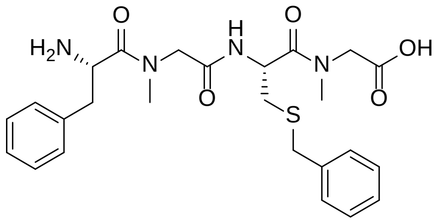

NH_2-Phe-NMeGly-Cys(Bn)-NMeGly-OH (15)

The trifluoroacetate salt of NH_2-Phe-*N*-MeGly-Cys(Bzl)-*N*-MeGly-OH **(15)** was obtained following the previously described SPPS protocol. Trifluoroacetate salt measuring 307 mg (0,61mmol) was obtained as a white solid (75%). **^{1}H NMR (400 MHz, D_2O) multiple conformers were observed**, δ (ppm): 7.11 – 7.39 (m, 10H), 4.72 – 4.77 (m, 1Hb), 4.63 – 4.69 (m, 1Hb), 4.47-4.52 (m, 1Ha), 4.40 – 4.55 (m, 1Ha), 3.53 – 4.19 (m, 6H), 2.98 – 3.18 (m, 2H), 2.73 – 2.92 (m, 7H), 2.52 – 2.66 (m, 1H). **^{13}C NMR (100 MHz, D_2O) multiple conformers were observed**, δ (ppm): 172.2 – 172.3 (1C), 171.9 – 172.0 (1C), 169.6 – 169.8 (1C), 168.8 – 169.3 (1C), 138.2, 133.3, 129.6, 129.4, 129.2, 129.1, 128.9, 128.8, 128.1, 127.5, 120.7, 53.8, (2C), 51.7, 50.2 - 50.7 (1C), 49.1, 35.7 – 36.8 (3C), 35.0 – 35.2 (1C), 31.4-32.6 (1C). **ESI-MS** *m*/*z* calc. for $C_{25}H_{33}N_4O_4S$ ($[M+H]^+$) 501.24, found 501.30.

4. NMR Spectra and ESI-MS data of Compounds

Cyclo-[Ala-Leu-D-Phe-Gly] (16)

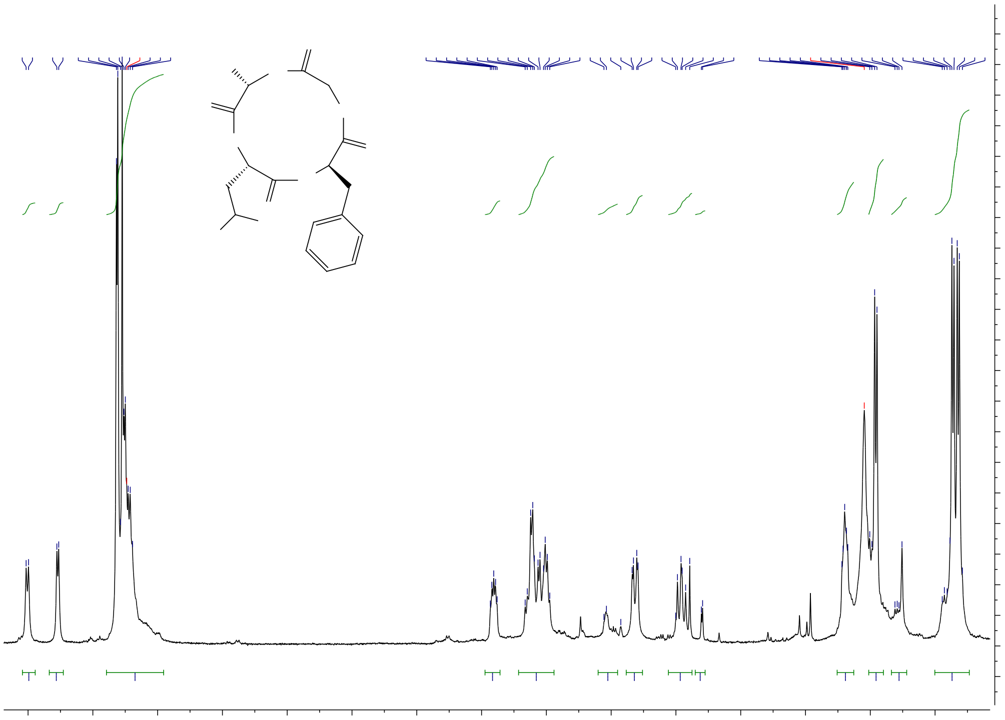

*^{1}H NMR (400 MHz, $CDCl_3$, 0.1% $MeOD_{d\text{-}4}$) spectrum of **16**.*

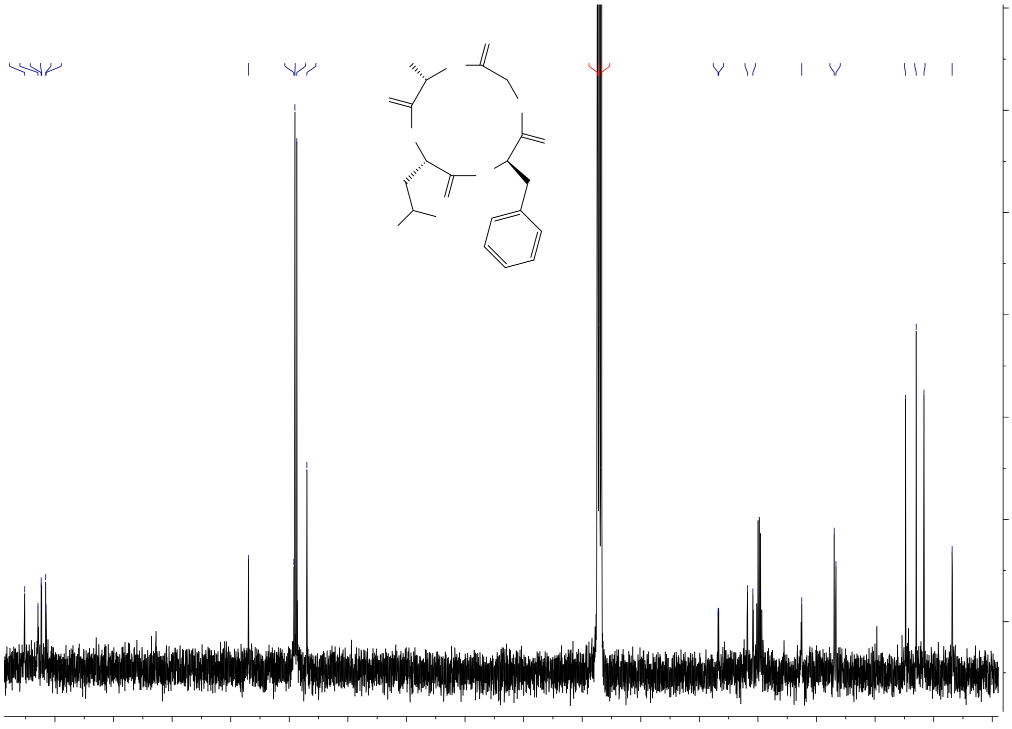

*^{13}C NMR (100 MHz, $CDCl_3$, 0.1%$MeOD_{d\text{-}4}$ (50.3 – 49.1 ppm)) spectrum of **16**.*

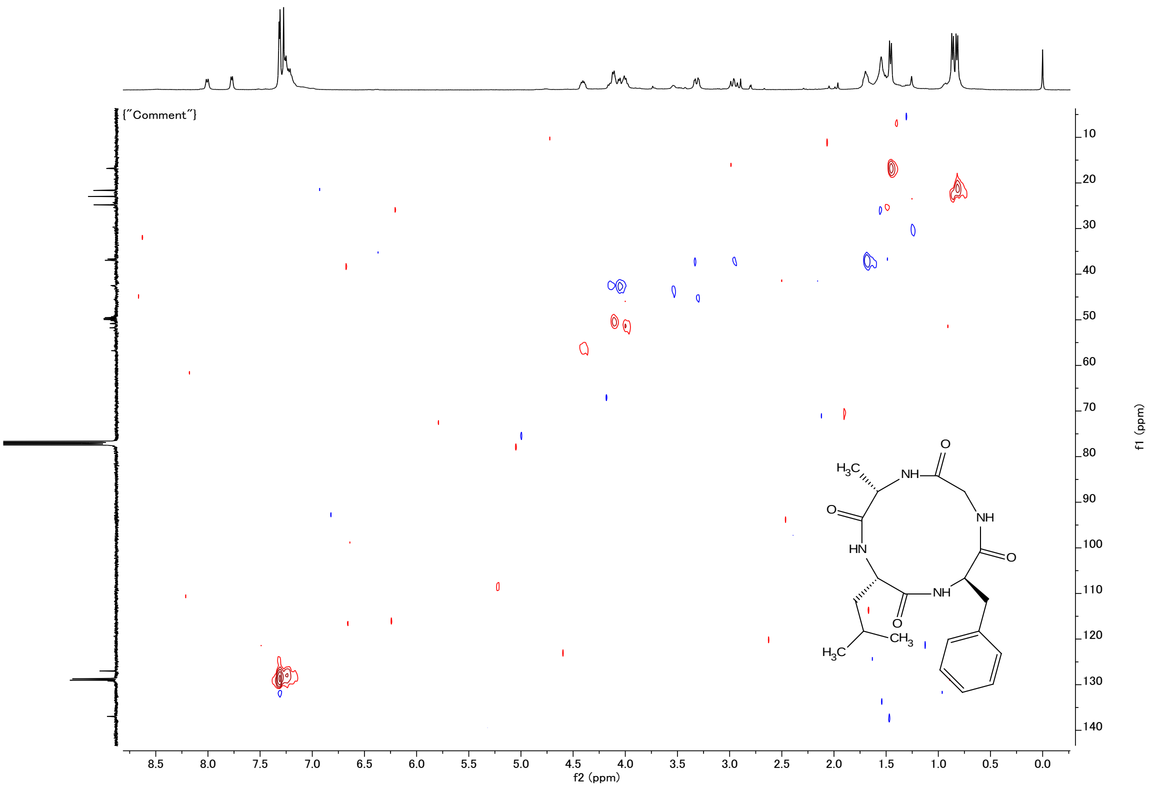

*HSQC (CDCl$_3$, 0.1%MeOD$_{d\text{-}4}$) spectrum of **16**.*

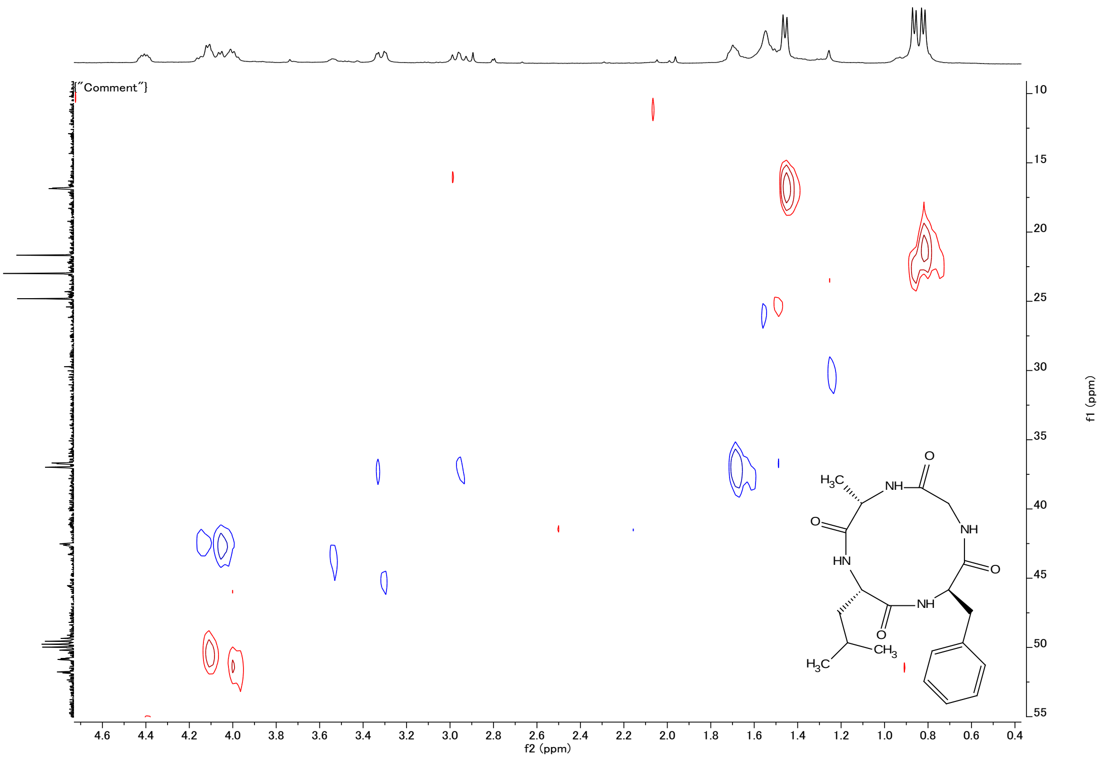

*HSQC (CDCl$_3$, 0.1%MeOD$_{d\text{-}4}$; 10 to 55 ppm C vs 0.4 to 4.6 ppm H) spectrum of **16**.*

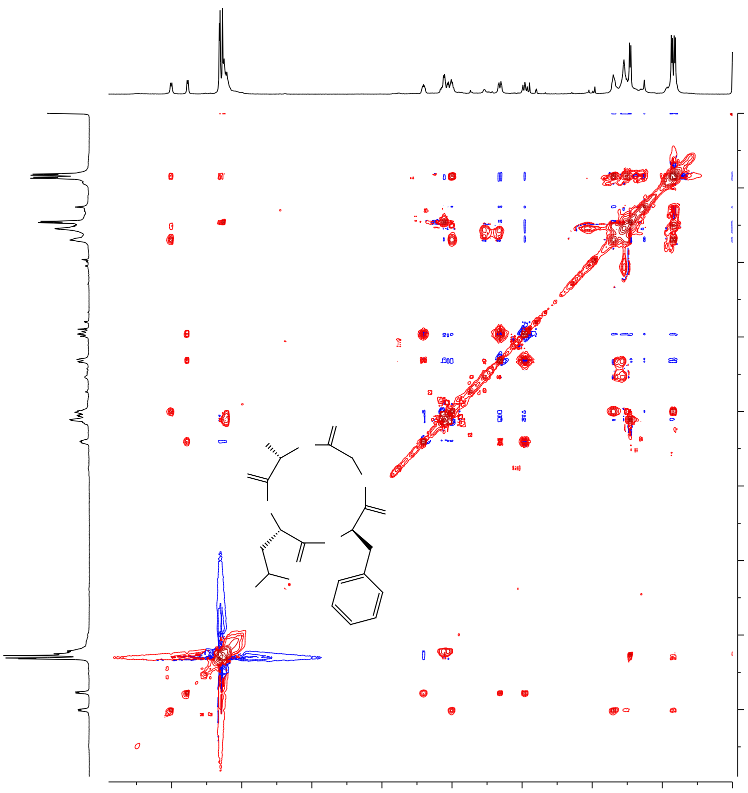

TOCSY ($CDCl_3$, 0.1%MeOD$_{d\text{-}4}$) spectrum of ***16***

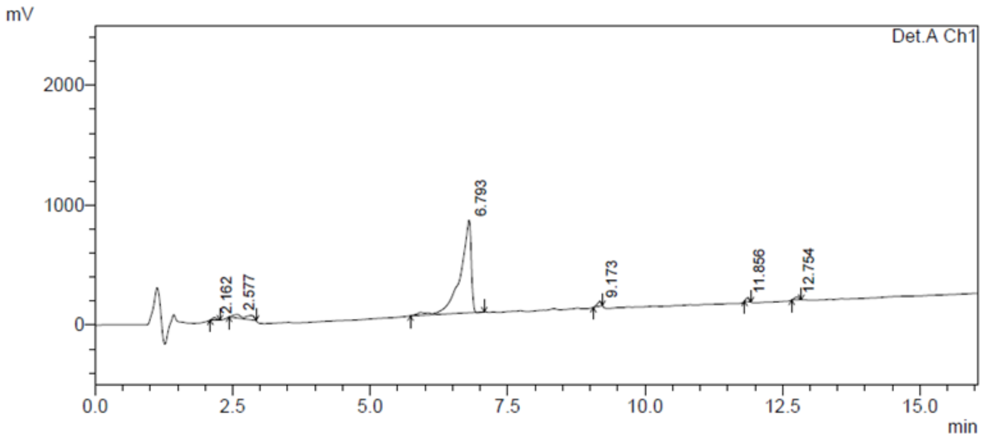

PeakTable

Detector A Ch1 220nm

Peak#	Ret. Time	Area	Height	Area %	Height %
1	2.162	110235	19747	0.934	2.158
2	2.577	608773	28209	5.158	3.083
3	6.793	10632089	774032	90.082	84.603
4	9.173	194273	38795	1.646	4.240
5	11.856	119972	30348	1.016	3.317
6	12.754	137402	23770	1.164	2.598
Total		11802744	914901	100.000	100.000

HPLC Chromatogram of ***16****. Linear gradient,* t0´- 30% B, t15´- 90% B, *25ºC, 220 nm. A: H_2O, 0.1% formic acid, B: MeCN, 0.1% formic acid.*

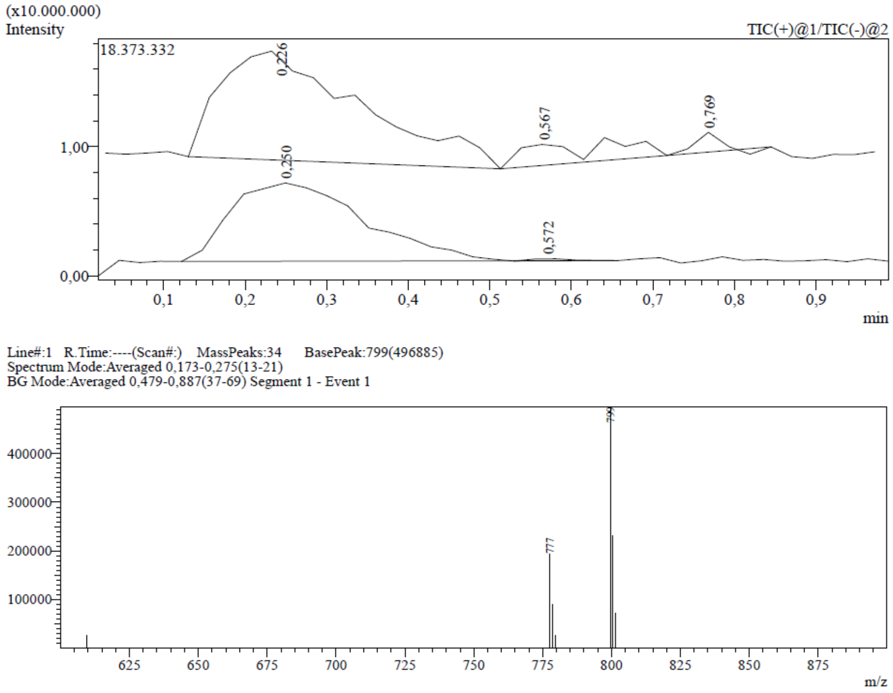

*ESI-MS spectrum of **16**.*

Trifluoroacetate salt of NHMe-Ala-Leu-Phe-Gly-OH (5)

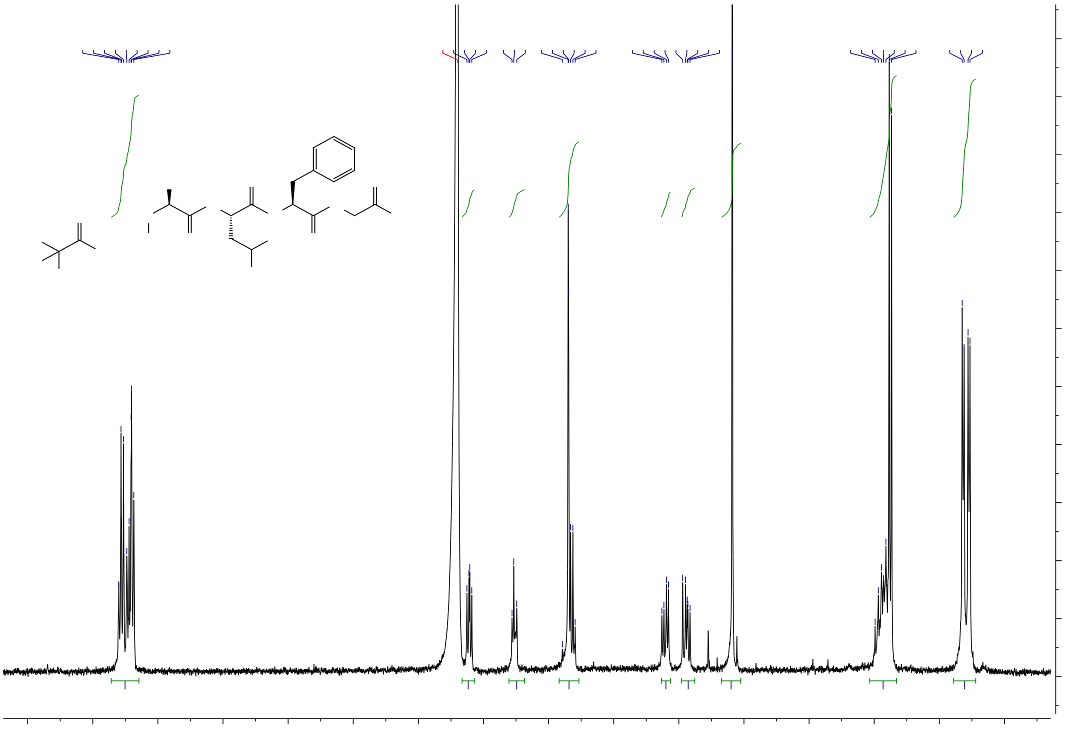

*^{1}H NMR (400 MHz, D_2O) spectrum of **5**.*

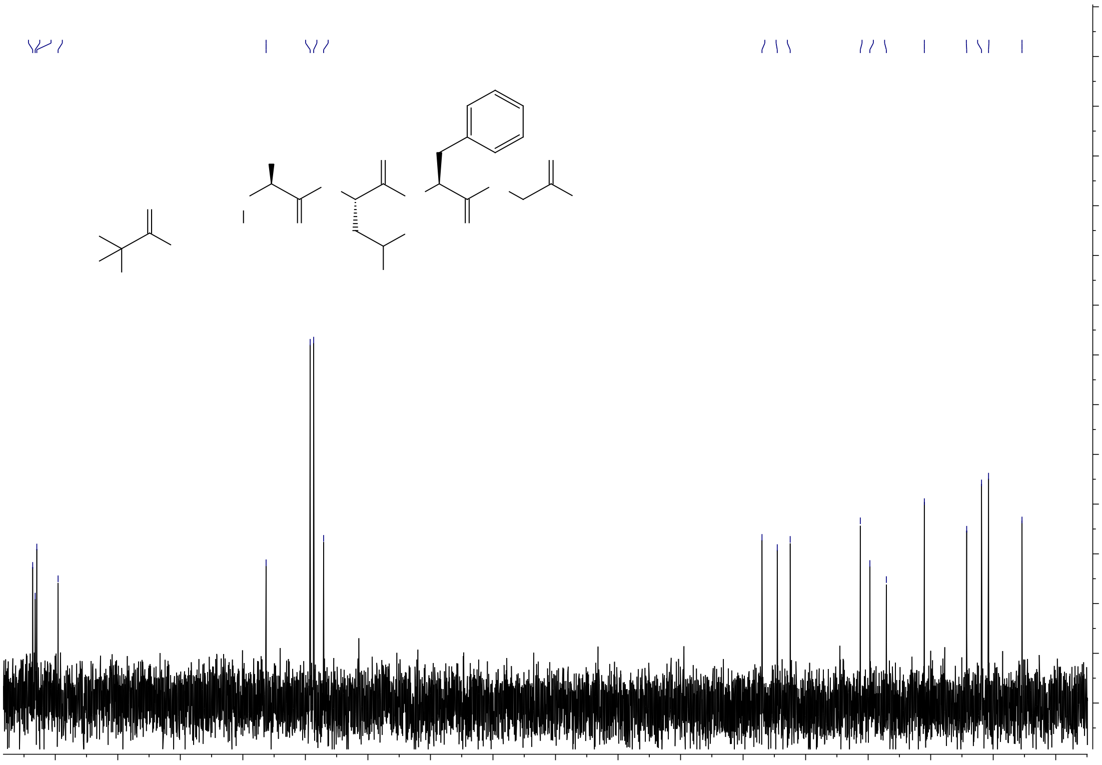

^{13}C *NMR (100 MHz,* D_2O*) spectrum of* ***5****.*

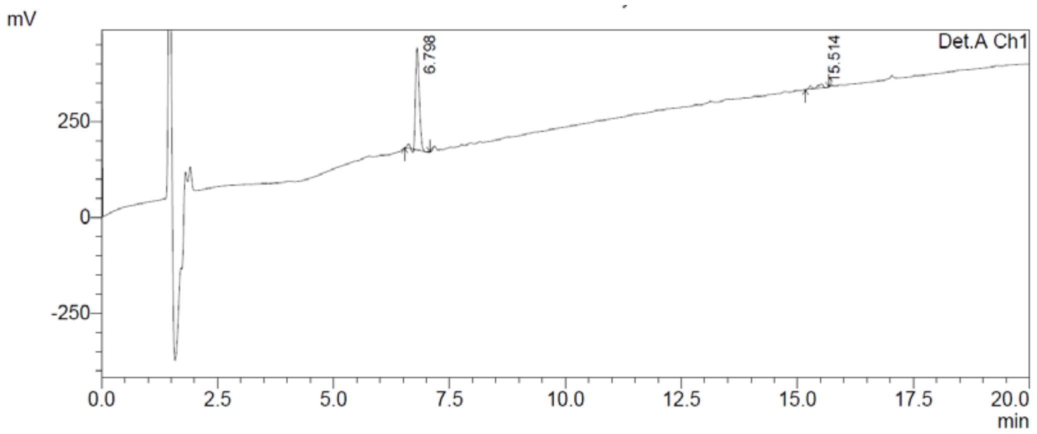

PeakTable

Detector A Ch1 220nm

Peak#	Ret. Time	Area	Height	Area %	Height %
1	6.798	1508659	266746	93.122	96.345
2	15.514	111429	10119	6.878	3.655
Total		1620088	276864	100.000	100.000

HPLC Chromatogram of ***5****. Conditions: Linear gradient,* t0′ - 8% B, t18′ - 90% B, *25°C, 220 nm. A:* H_2O*, 0.1% formic acid, B: MeCN, 0.1% formic acid.*

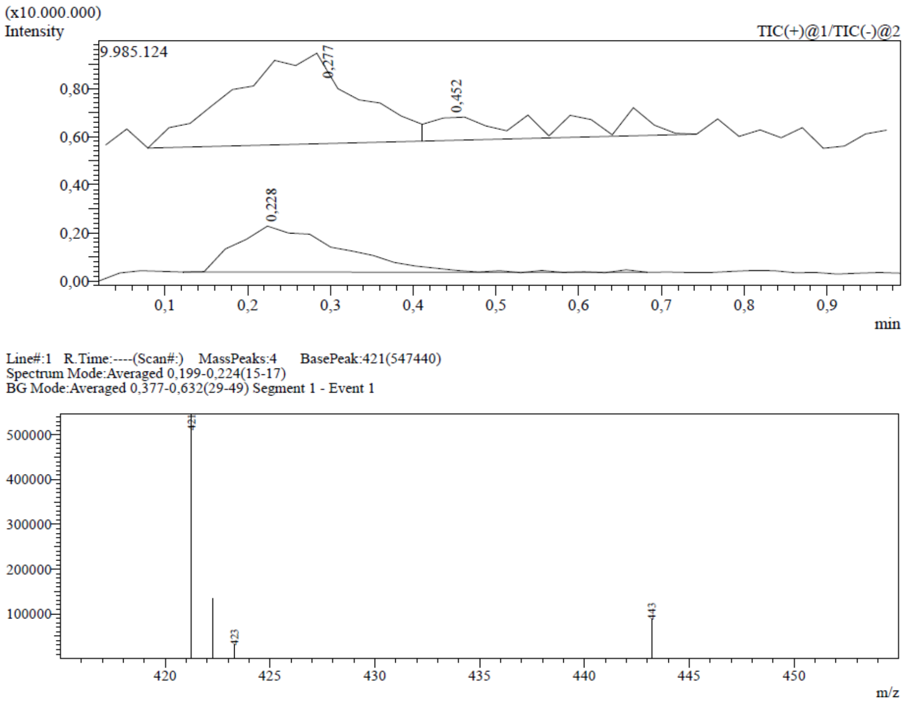

ESI-MS spectrum of ***5****.*

Cyclo-[N-MeAla-Leu-Phe-Gly] (17)

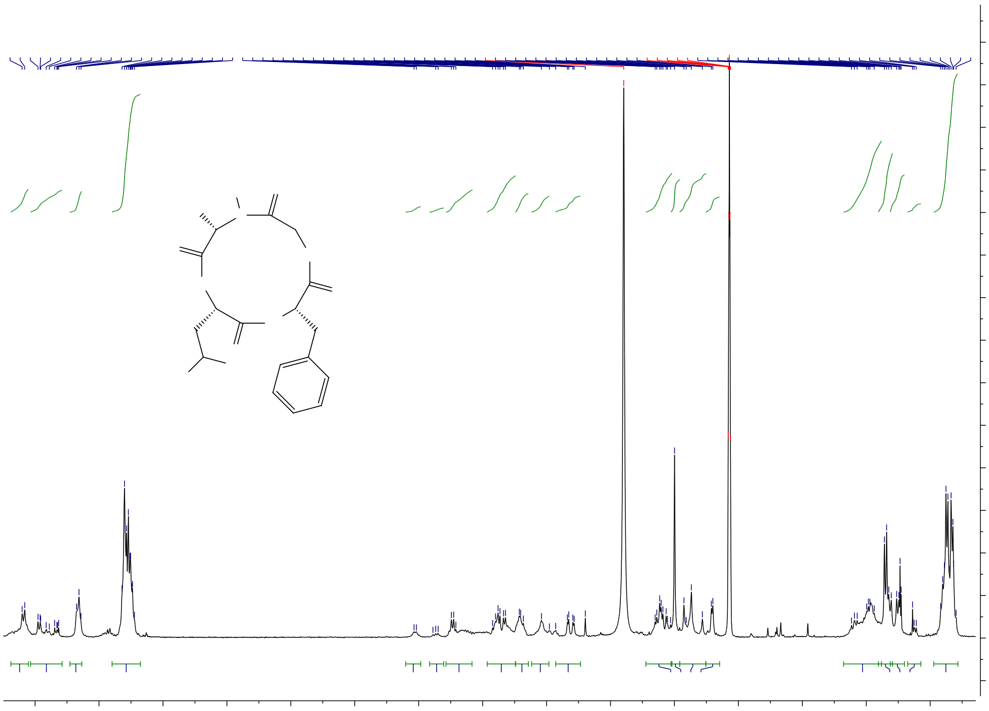

^{1}*H NMR (400 MHz, DMSO$_{d-6}$) spectrum of* ***17****.*

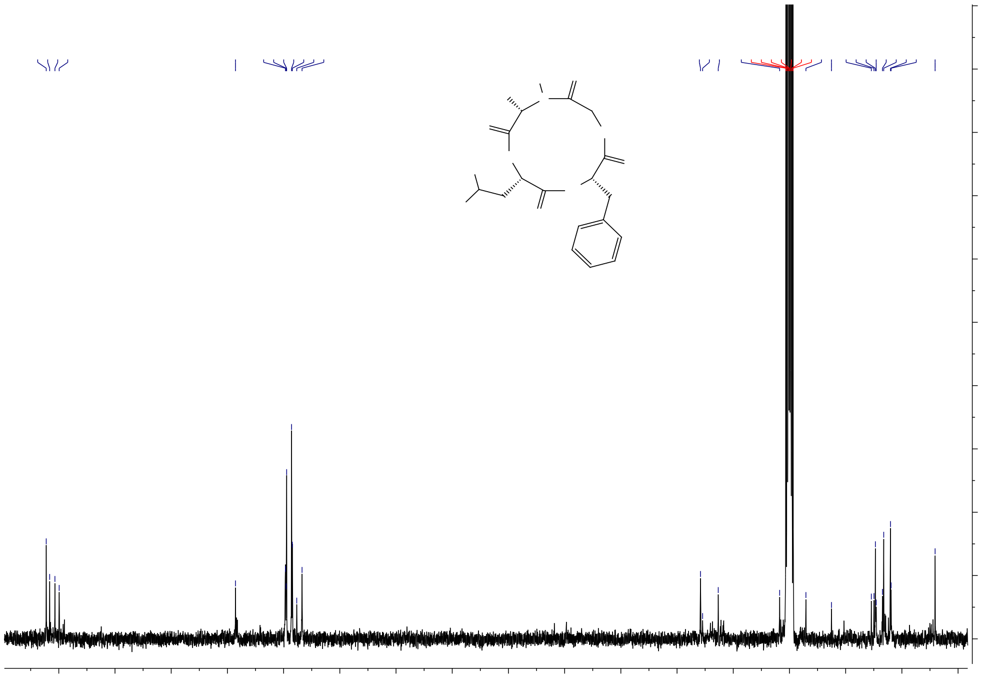

*^{13}C NMR (100 MHz, DMSO$_{d\text{-}6}$) spectrum of **17**.*

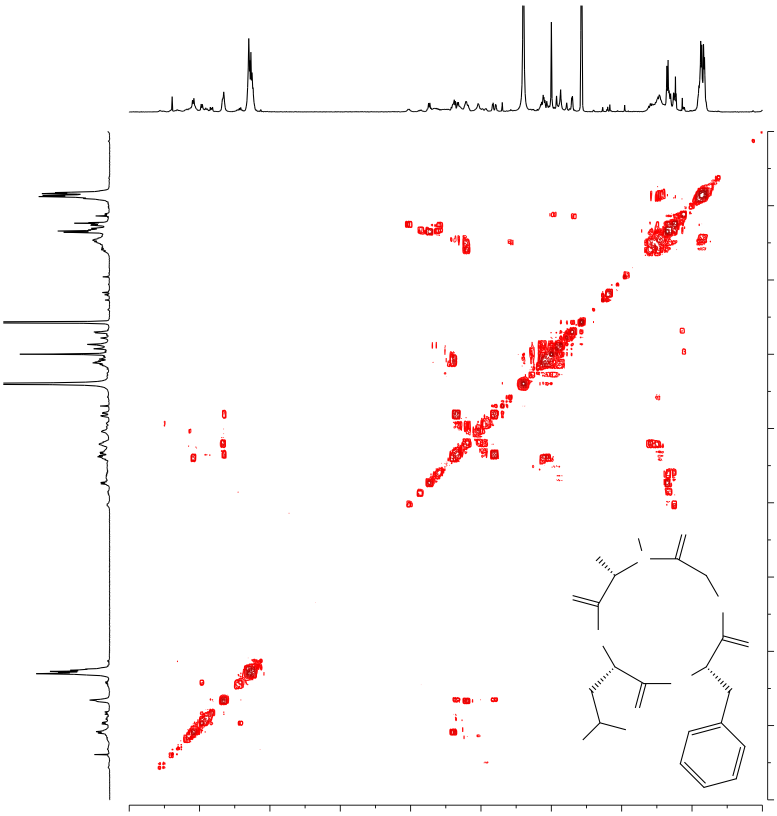

*COSY (400 MHz, DMSO$_{d\text{-}6}$) spectrum of **17**.*

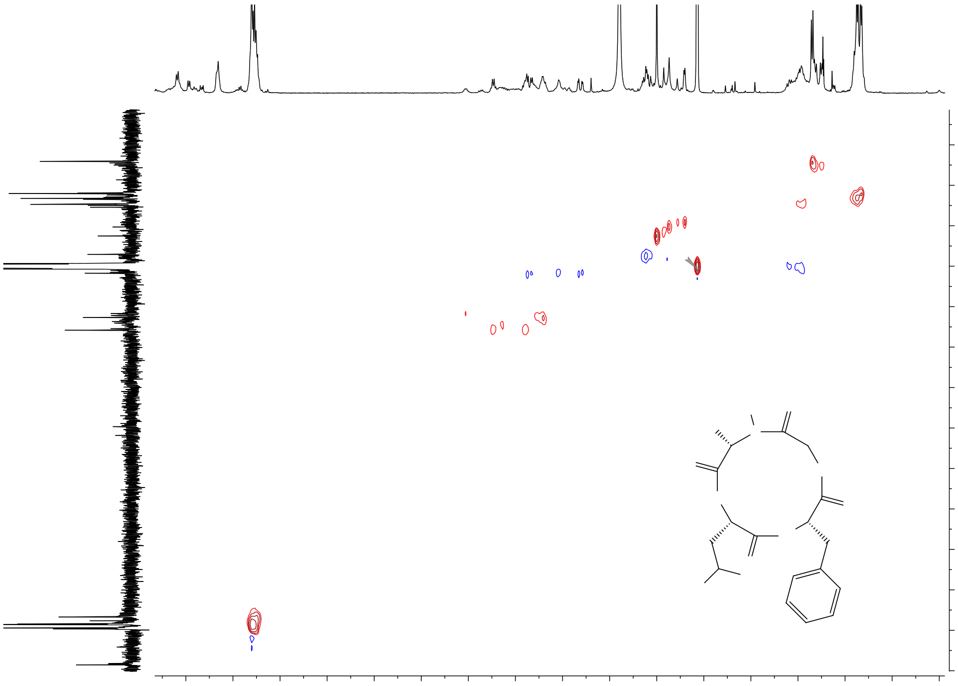

HSQC (DMSO$_{d\text{-}6}$) spectrum of ***17***

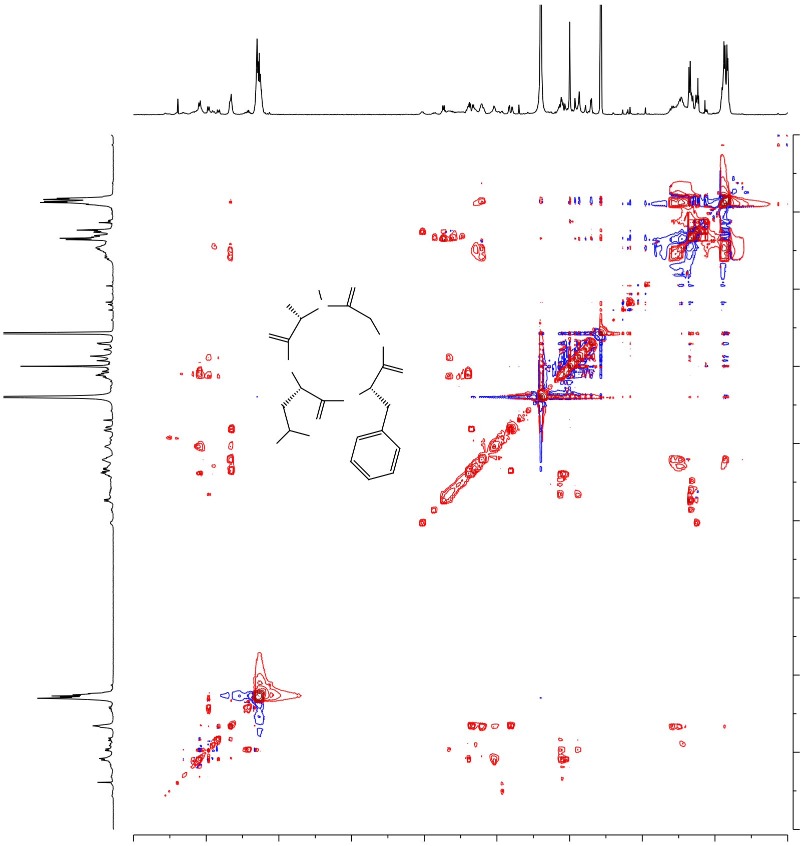

TOCSY (DMSO$_{d\text{-}6}$) spectrum of ***17****.*

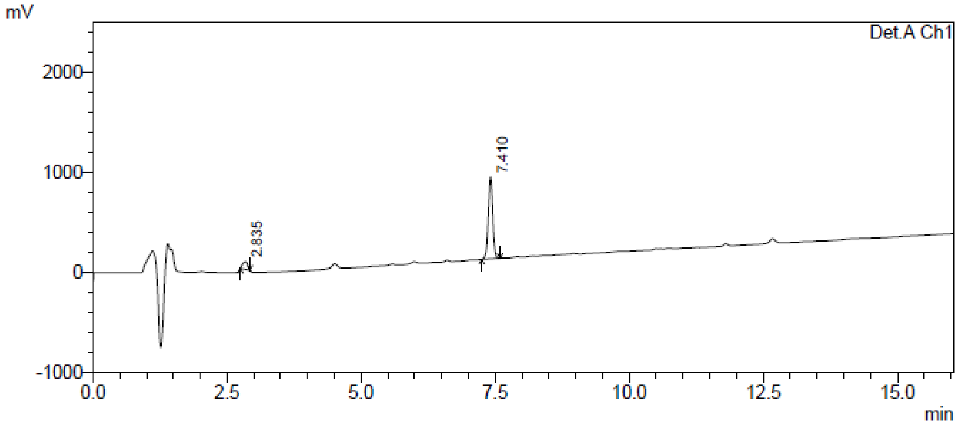

PeakTable

Detector A Ch1 220nm

Peak#	Ret. Time	Area	Height	Area %	Height %
1	2.835	487704	76662	9.310	8.564
2	7.410	4750709	818491	90.690	91.436
Total		5238412	895152	100.000	100.000

HPLC Chromatogram of ***17****. Conditions: Linear gradient,* t0′- 30% B, t15′- 90% B, *25°C, 220 nm. A:* H_2O*, 0.1% formic acid, B: MeCN, 0.1% formic acid.*

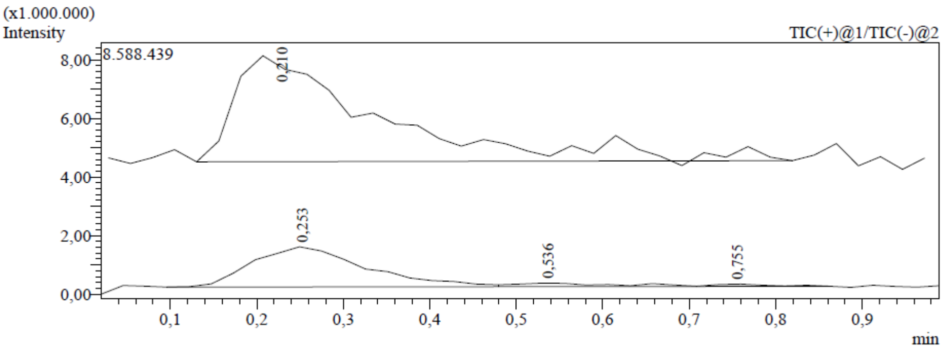

Line#:1 R.Time:----(Scan#:) MassPeaks:11 BasePeak:827(71650)
Spectrum Mode:Averaged 0,199-0,250(15-19)
BG Mode:Averaged 0,326-0,709(25-55) Segment 1 - Event 1

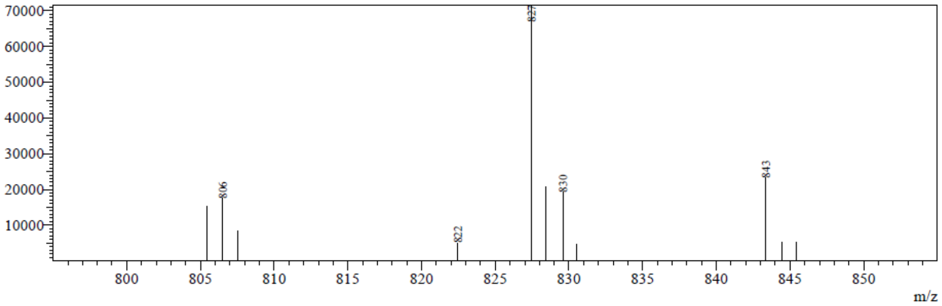

ESI-MS spectrum of ***17****.*

Cyclo-[Ala-Leu-Phe-_N_-MeGly] (18)

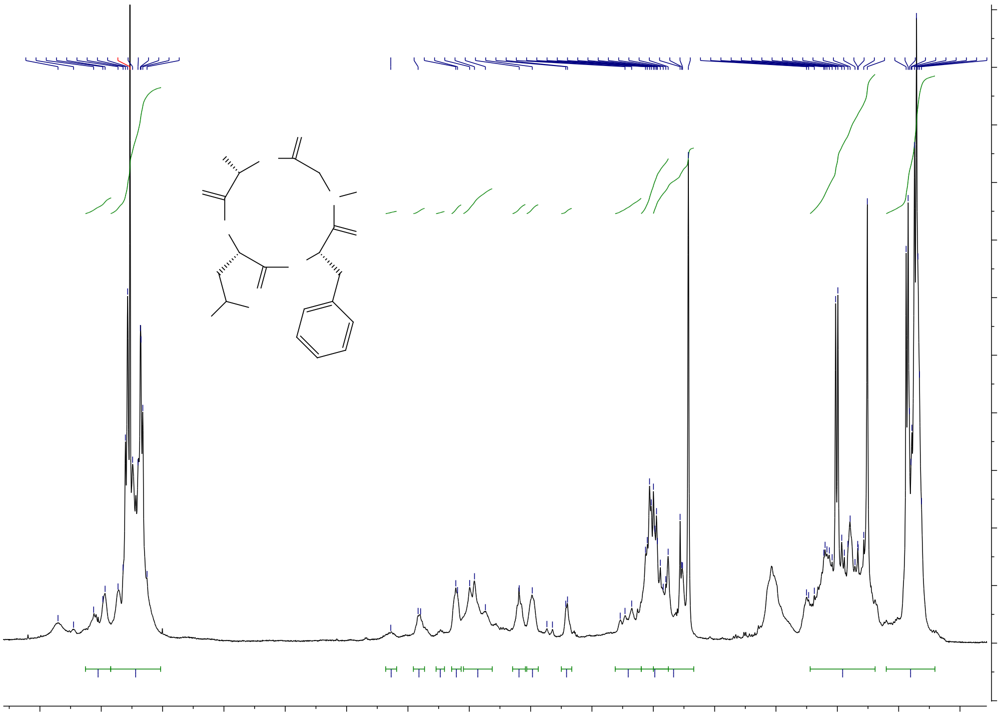

*^{1}H NMR (400 MHz, CDCl3) spectrum of **18**.*

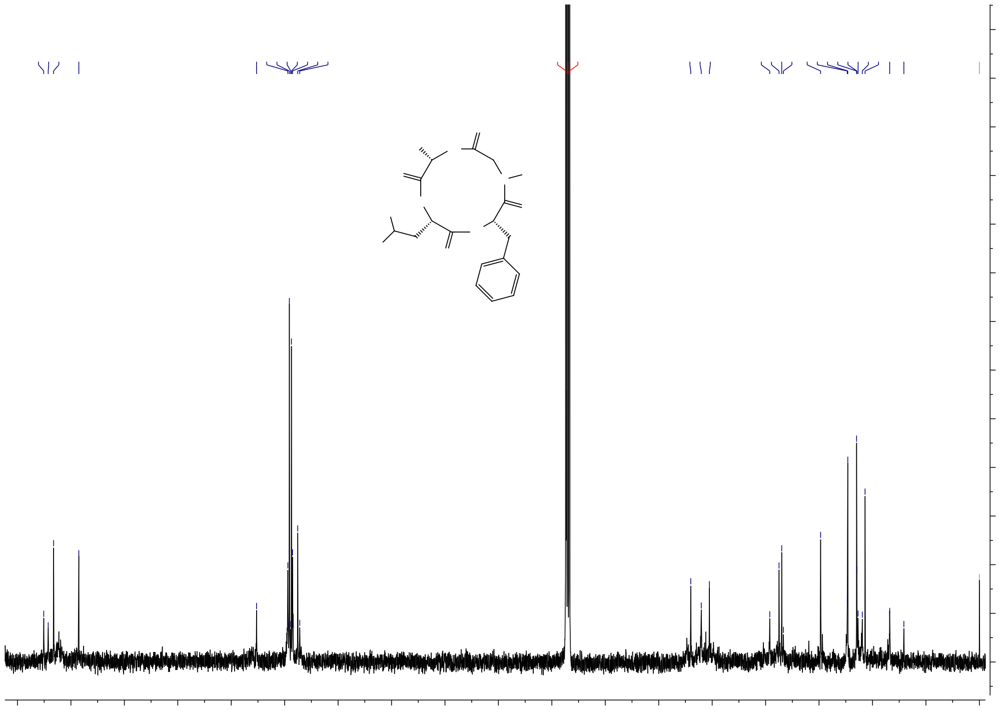

*^{13}C NMR (100 MHz, CDCl3) spectrum of **18**.*

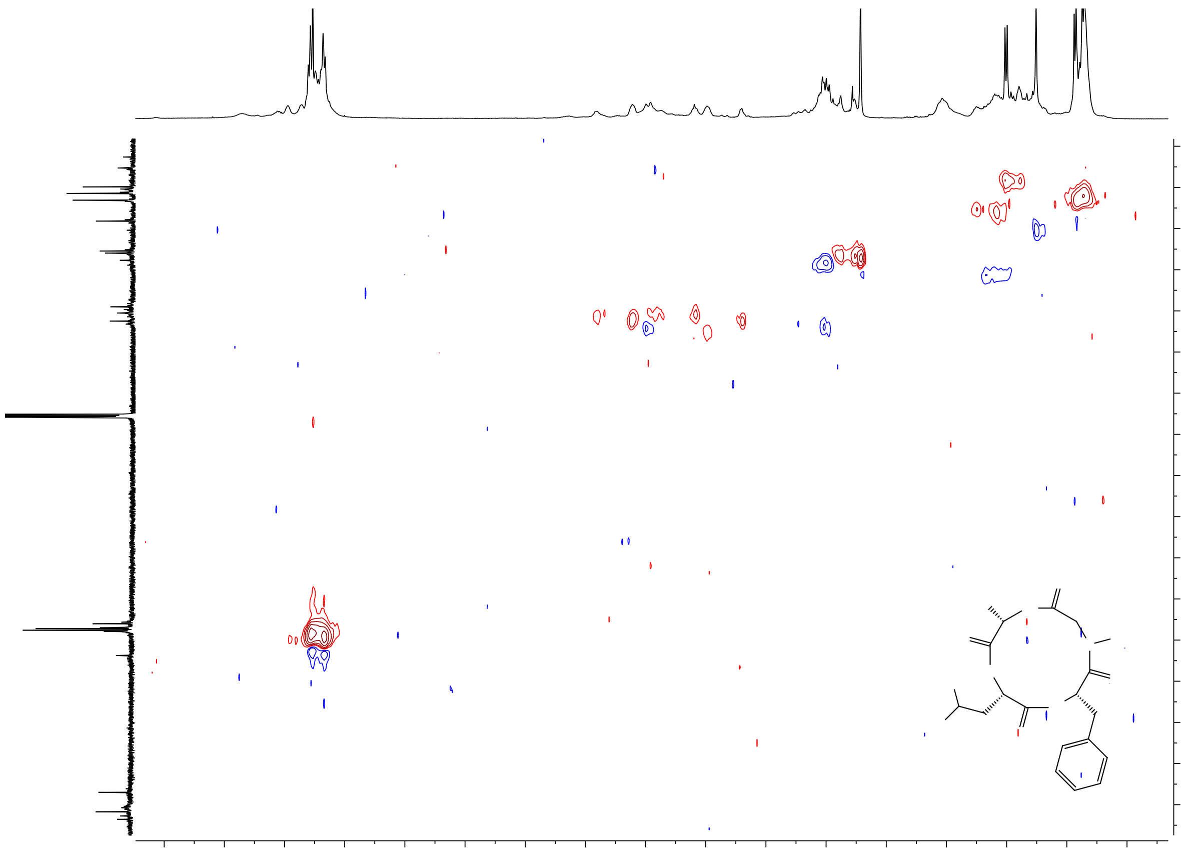

*HSQC (CDCl$_3$) spectrum of **18***

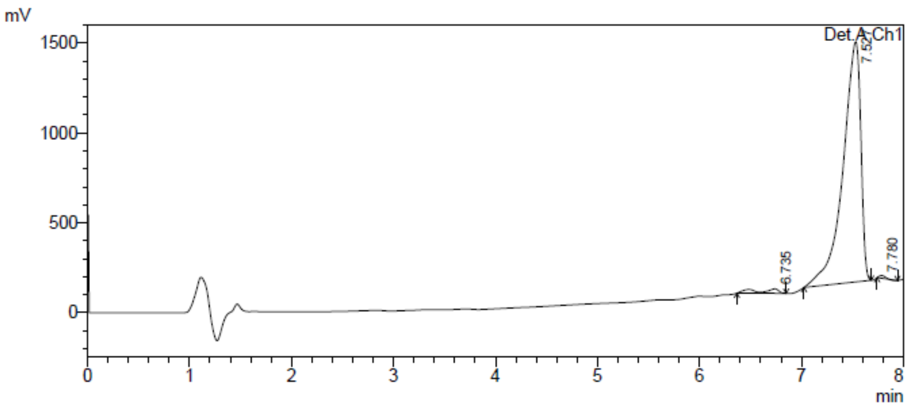

PeakTable

Detector A Ch1 220nm

Peak#	Ret. Time	Area	Height	Area %	Height %
1	6.735	348150	24792	2.096	1.800
2	7.527	16215925	1337289	97.610	97.087
3	7.780	48915	15333	0.294	1.113
Total		16612991	1377414	100.000	100.000

*HPLC Chromatogram of **18**. Conditions: Linear gradient,* t0´- 30% B, t10´- 90% B, *25ºC, 220 nm. A: H_2O, 0.1% formic acid, B: MeCN, 0.1% formic acid.*

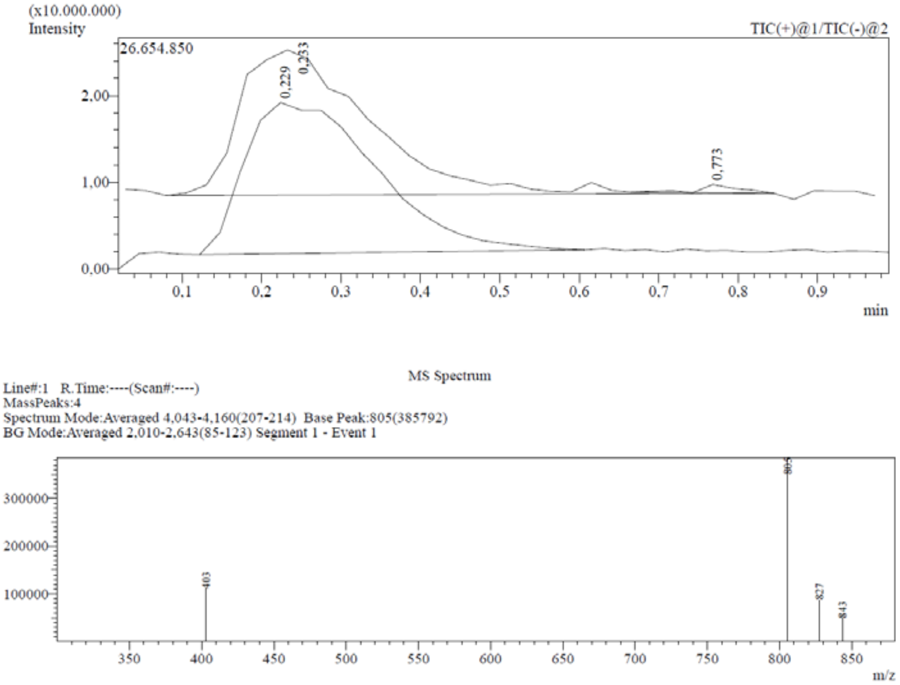

*ESI-MS spectrum of **18**.*

***Cyclo*-[Ala-Leu-*N*-Me-D-Phe-Gly] (19)**

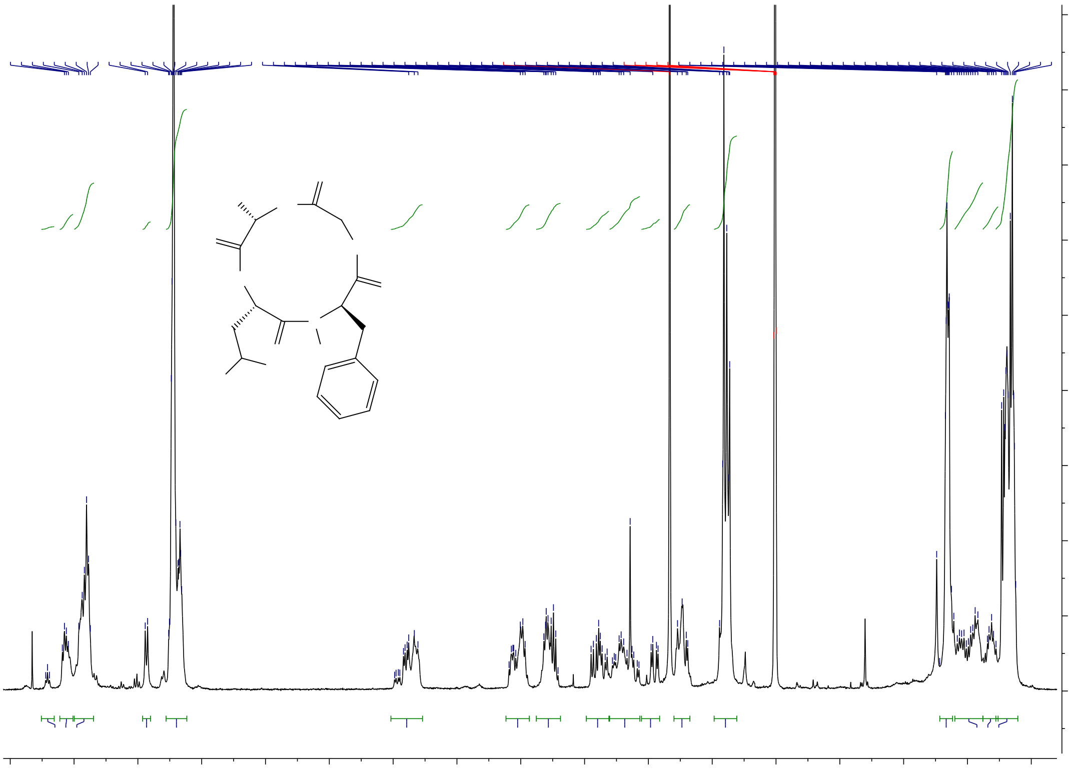

*^{1}H NMR (400 MHz, DMSO$_{d\text{-}6}$) spectrum of **19**.*

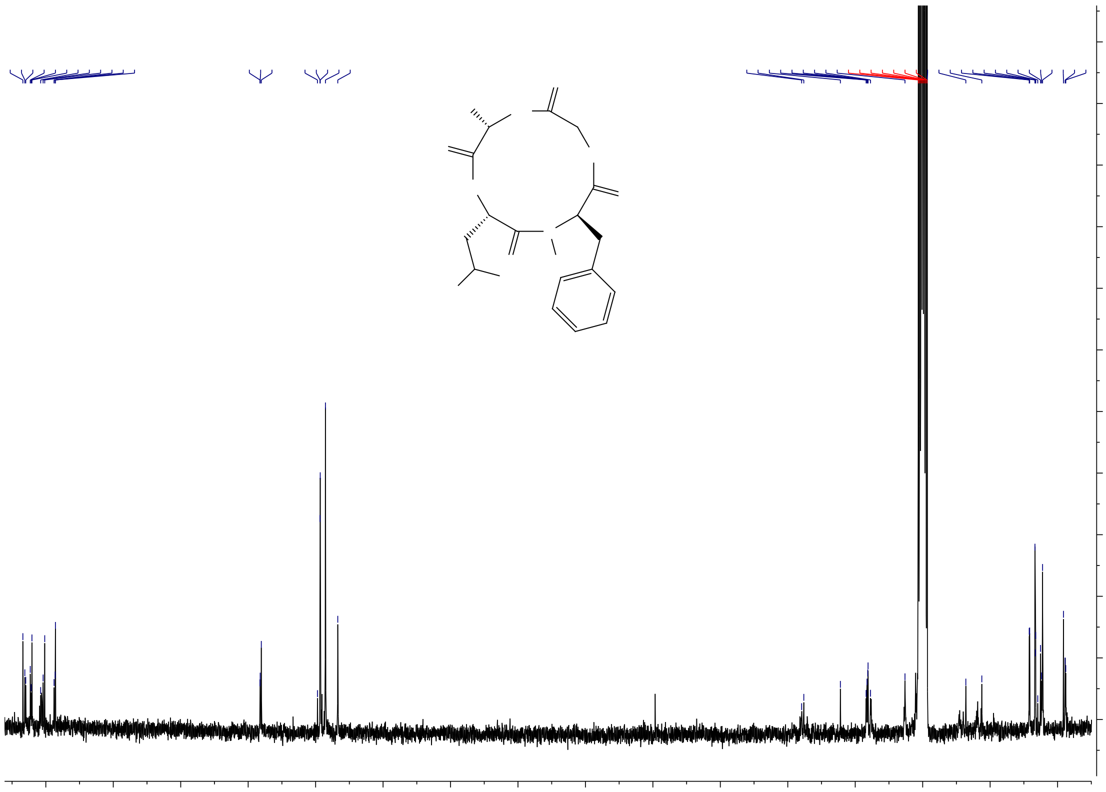

^{13}C NMR (100 MHz, DMSO$_{d-6}$) spectrum of **19**.

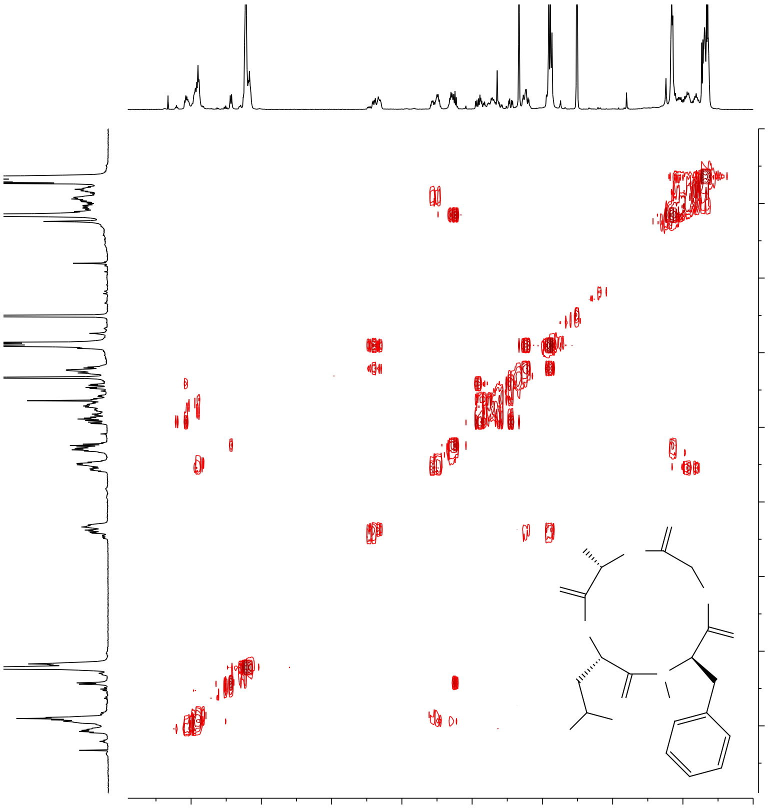

COSY (DMSO$_{d-6}$) spectrum of **19**.

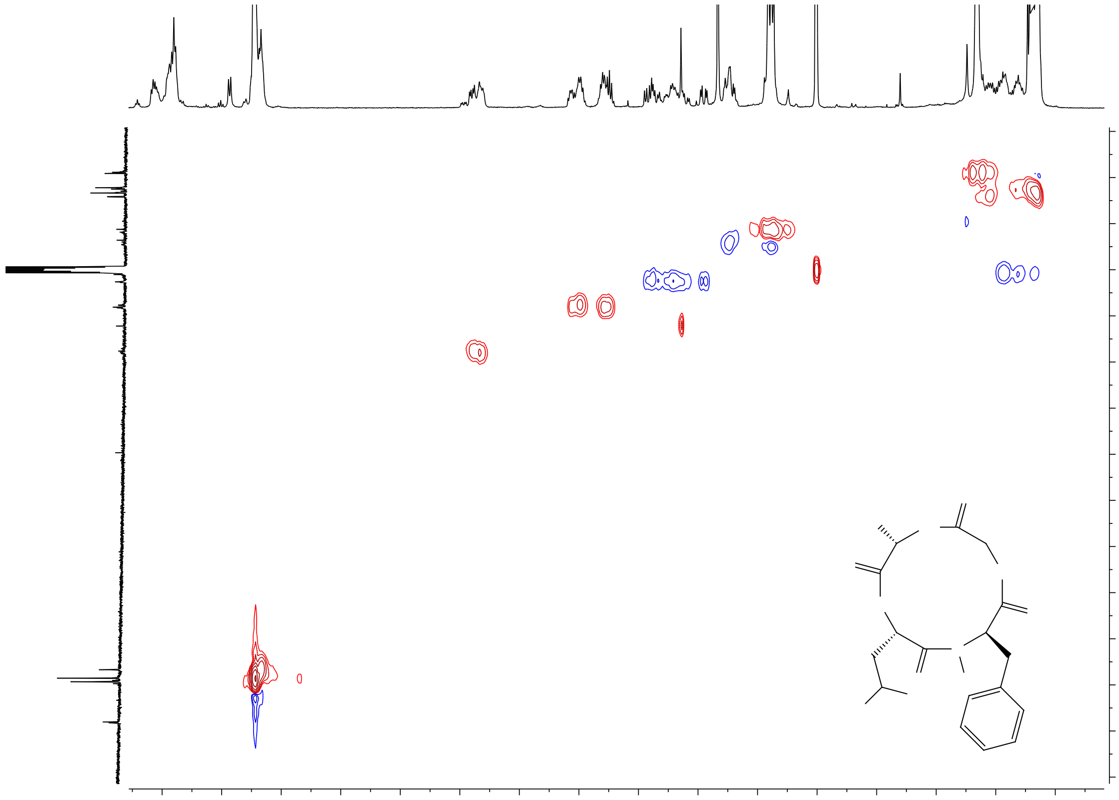

*HSQC (DMSO$_{d-6}$) spectrum of **19**.*

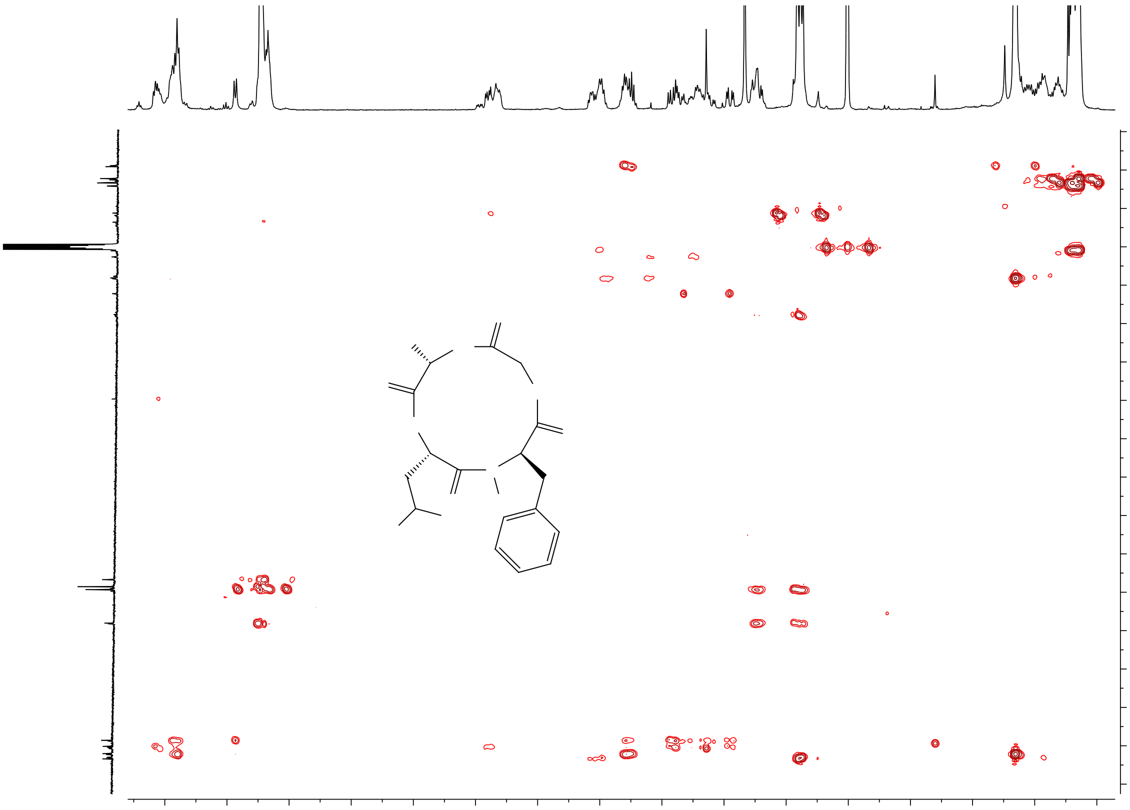

*HMBC (DMSO$_{d-6}$) spectrum of **19**.*

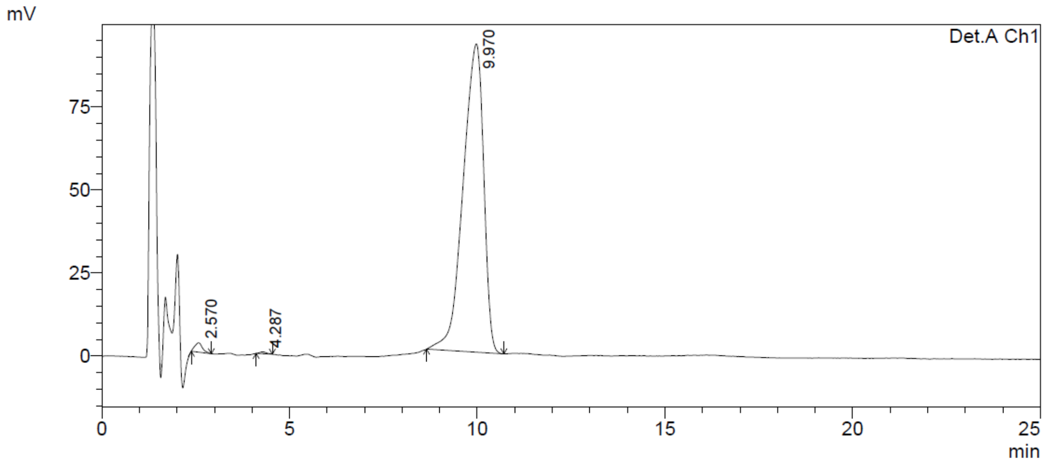

PeakTable

Detector A Ch1 220nm

Peak#	Ret. Time	Area	Height	Area %	Height %
1	2.570	39355	2803	1.101	2.911
2	4.287	6968	566	0.195	0.587
3	9.970	3529713	92920	98.705	96.501
Total		3576036	96289	100.000	100.000

*HPLC Chromatogram of **19**. Isocratic flow, MeOH: H_2O (70:30), 30°C.*

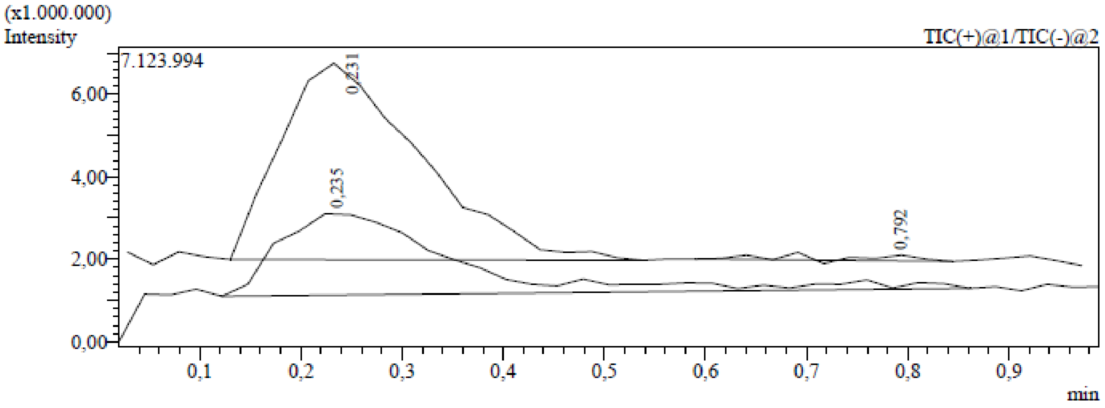

Line#:1 R.Time:----(Scan#:) MassPeaks:2 BasePeak:828(110505)
Spectrum Mode:Averaged 0,173-0,224(13-17)
BG Mode:None Segment 1 - Event 1

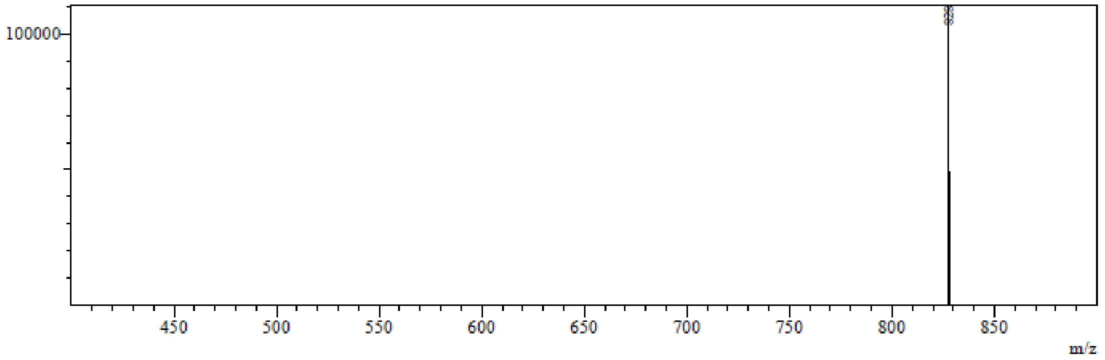

*ESI-MS spectrum of **19**.*

Trinfluoroacetate salt of NH-MeAla-Leu-D-Phe-Gly-OH (8)

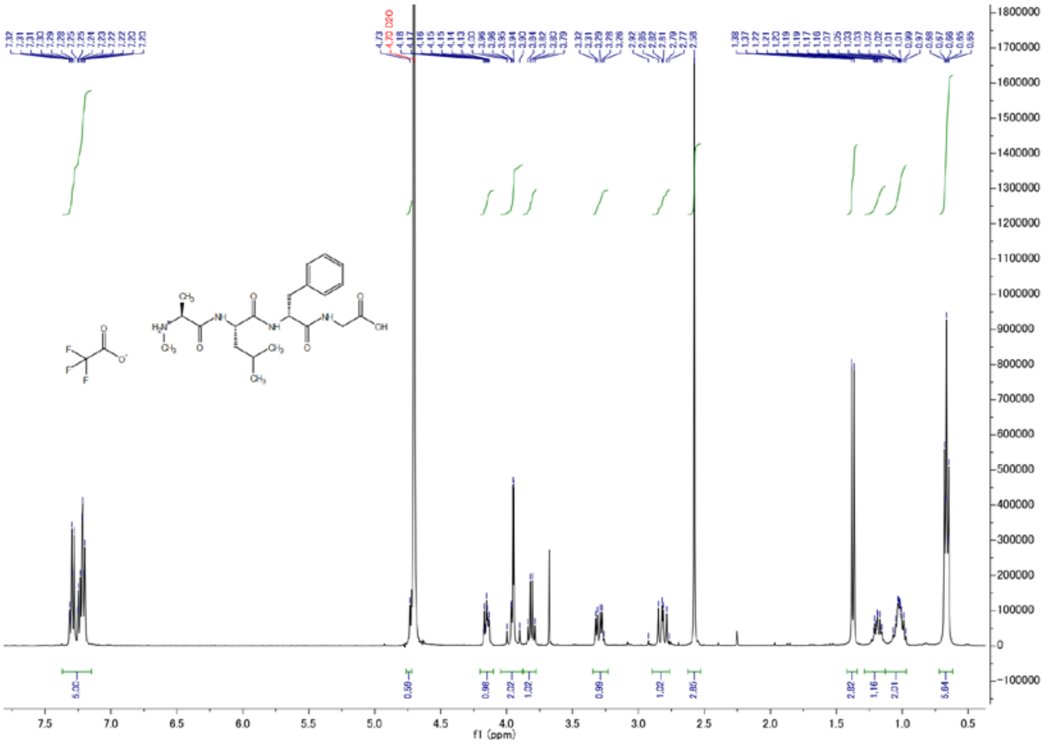

^{1}H NMR (400 MHz, D_2O) spectrum of **8**.

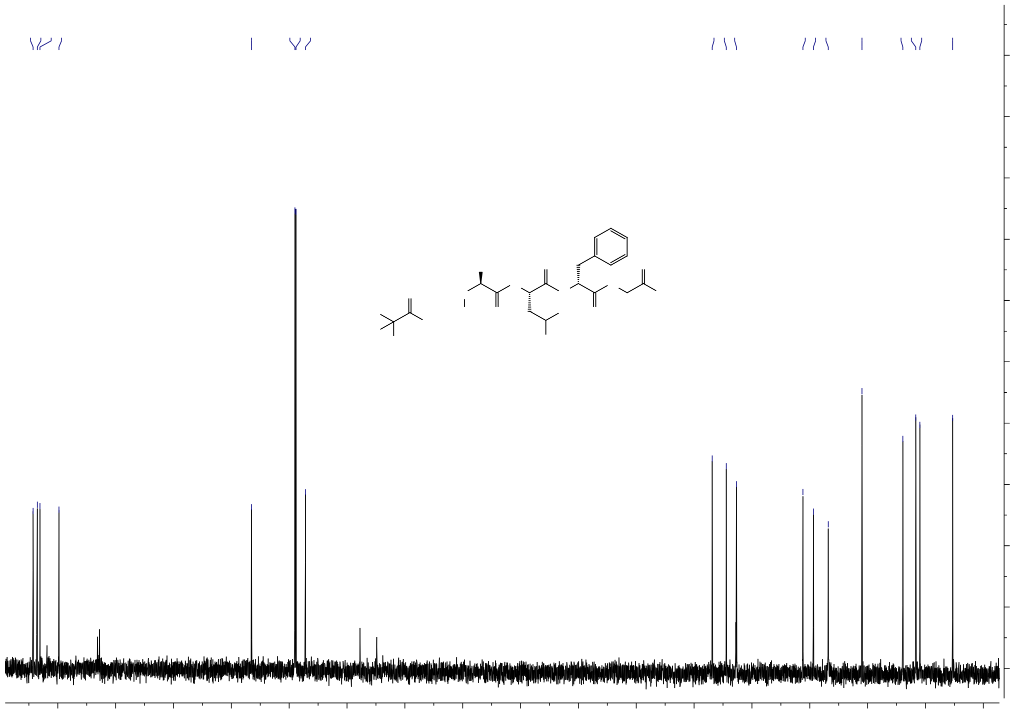

^{13}C NMR (400 MHz, D_2O) spectrum of **8**.

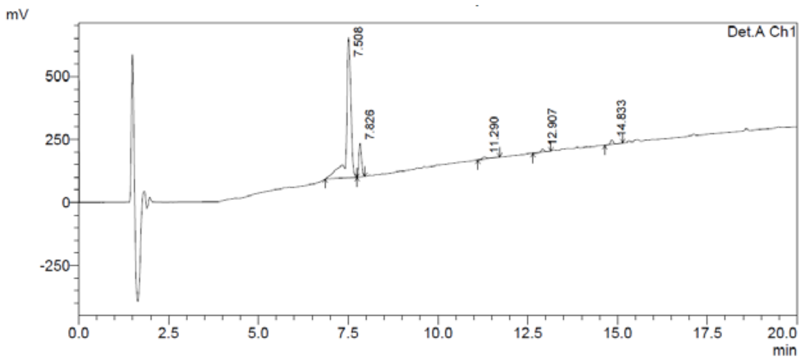

PeakTable

Detector A Ch1 220nm

Peak#	Ret. Time	Area	Height	Area %	Height %
1	7.508	5006165	555125	84.095	76.266
2	7.826	693634	134693	11.652	18.505
3	11.290	81604	8388	1.371	1.152
4	12.907	68740	13351	1.155	1.834
5	14.833	102821	16320	1.727	2.242
Total		5952964	727878	100.000	100.000

HPLC Chromatogram of **8**. *Conditions: Linear gradient*, t0´- 8% B, t18´- 90% B, *25ºC, 220 nm. A: H_2O, 0.1% formic acid, B: MeCN, 0.1% formic acid.*

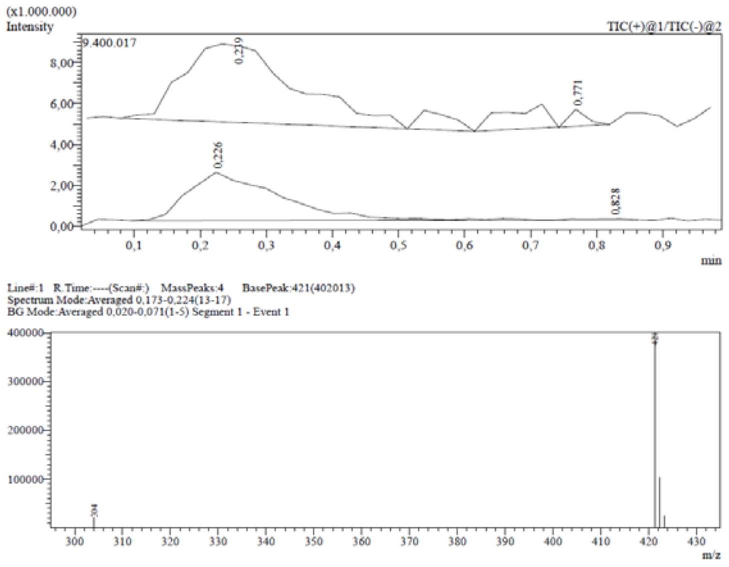

ESI-MS spectrum of **8**.

Cyclo-[_N_-MeAla-Leu-D-Phe-Gly] (20)

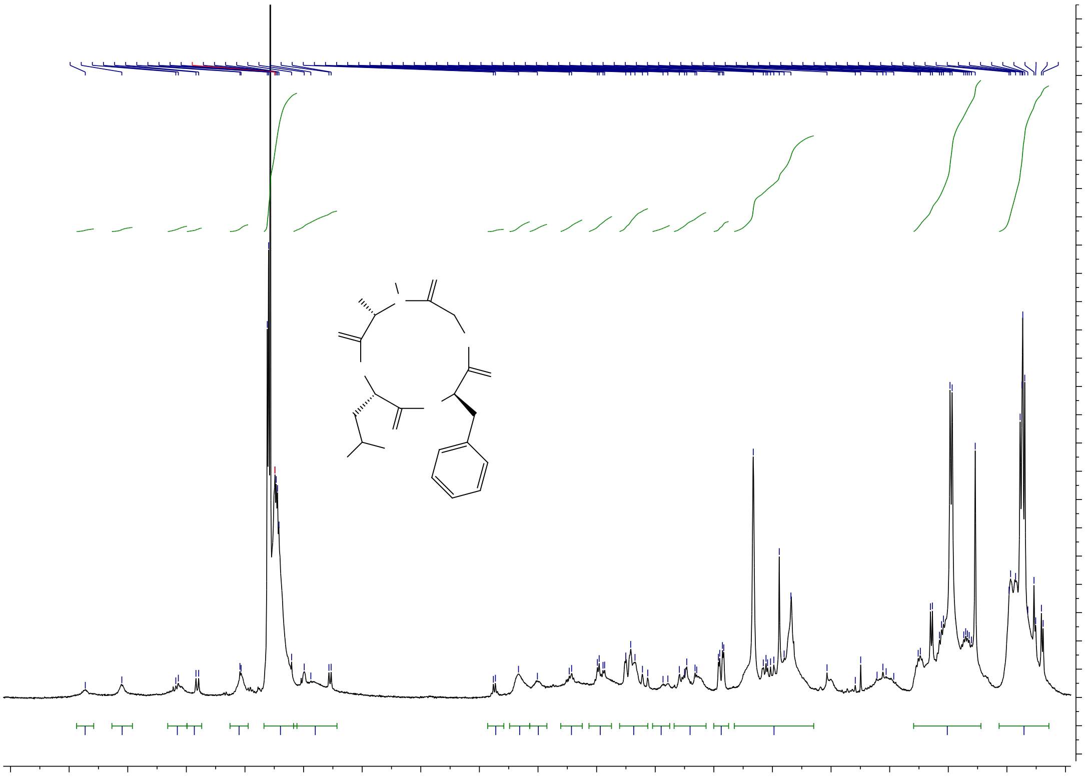

^{1}H NMR (400 MHz, $CDCl_3$) spectrum of ***20****.*

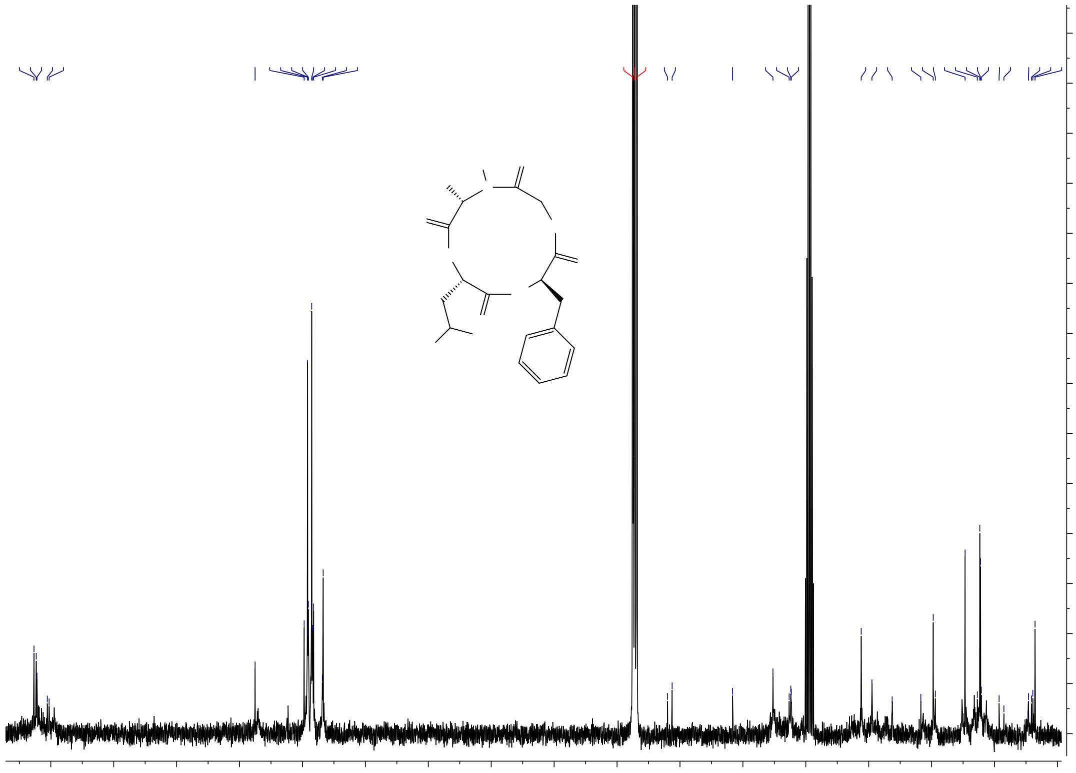

^{13}C NMR (100 MHz, $CDCl_3$, 0.1% $MeOD_{d\text{-}4}$) spectrum of ***20****.*

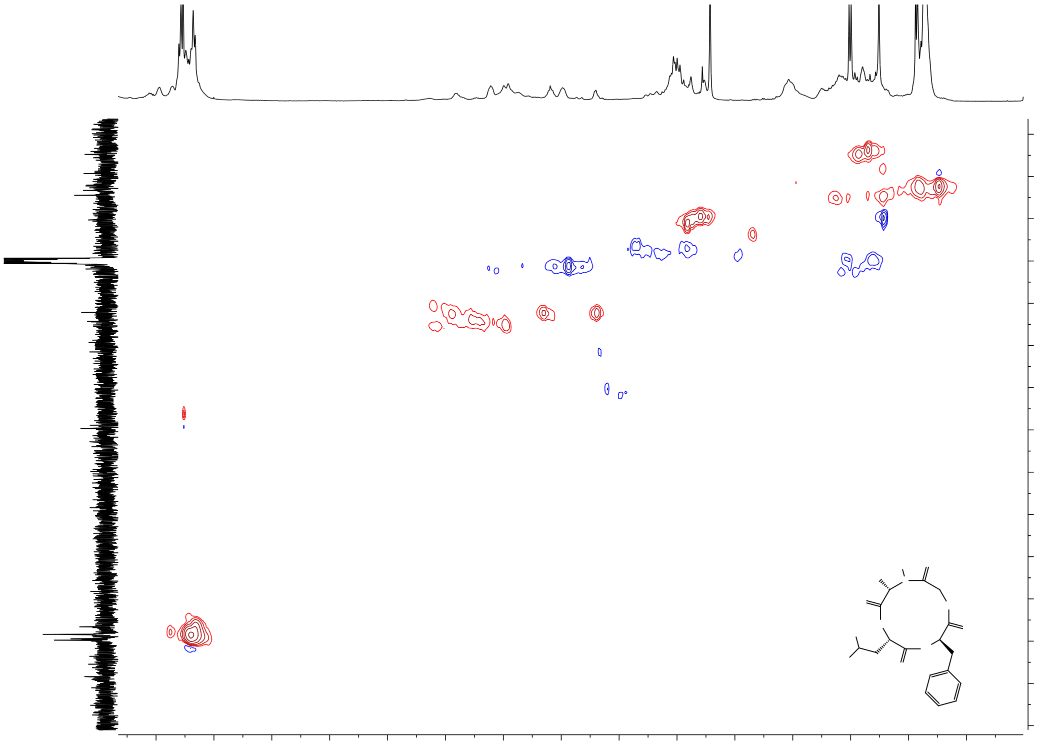

HSQC (CDCl₃, 0.1% MeOD$_{d-4}$) spectrum of ***20***.

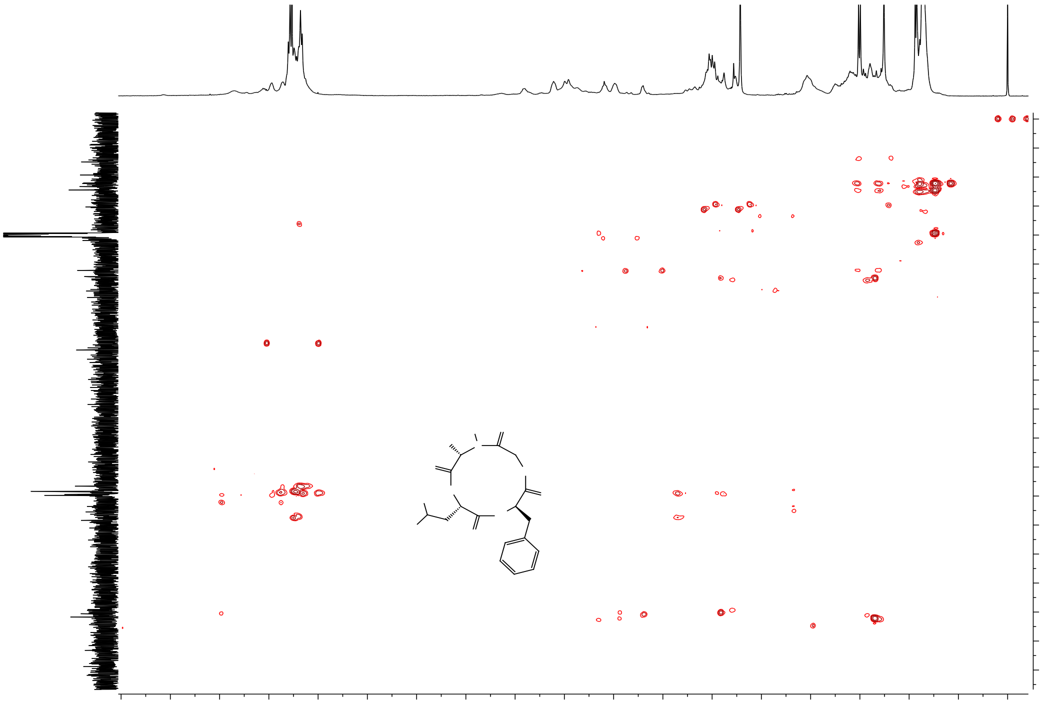

HMBC (CDCl₃, 0.1% MeOD$_{d-4}$) spectrum of ***20***.

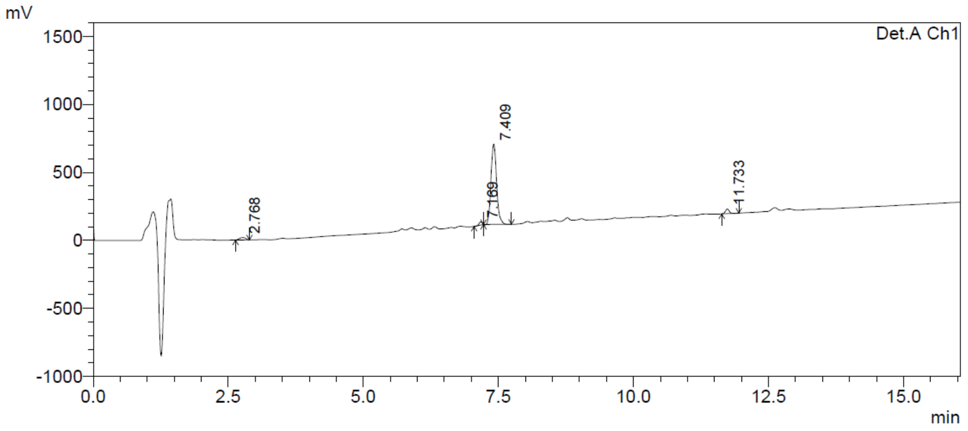

PeakTable

Detector A Ch1 220nm

Peak#	Ret. Time	Area	Height	Area %	Height %
1	2.768	137588	17382	2.962	2.614
2	7.169	88798	23894	1.912	3.594
3	7.409	4257360	589886	91.650	88.726
4	11.733	161479	33675	3.476	5.065
Total		4645226	664836	100.000	100.000

HPLC Chromatogram of ***20****. Conditions: Linear gradient,* t0´- 30% B, t15´- 90% B, *25ºC, 220 nm. A: H_2O, 0.1% formic acid, B: MeCN, 0.1% formic acid.*

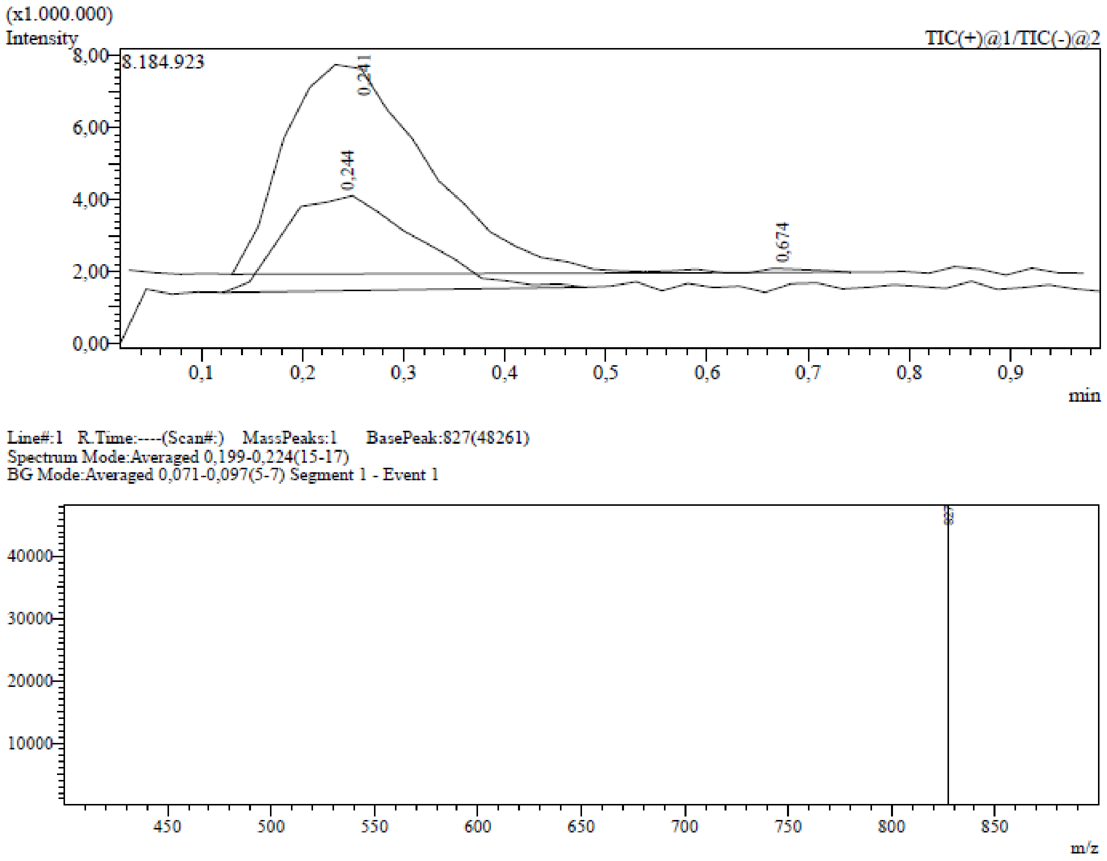

ESI-MS spectrum of ***20****.*

Cyclo-[Ala-Leu-D-Phe-_N_-MeGly] (21)

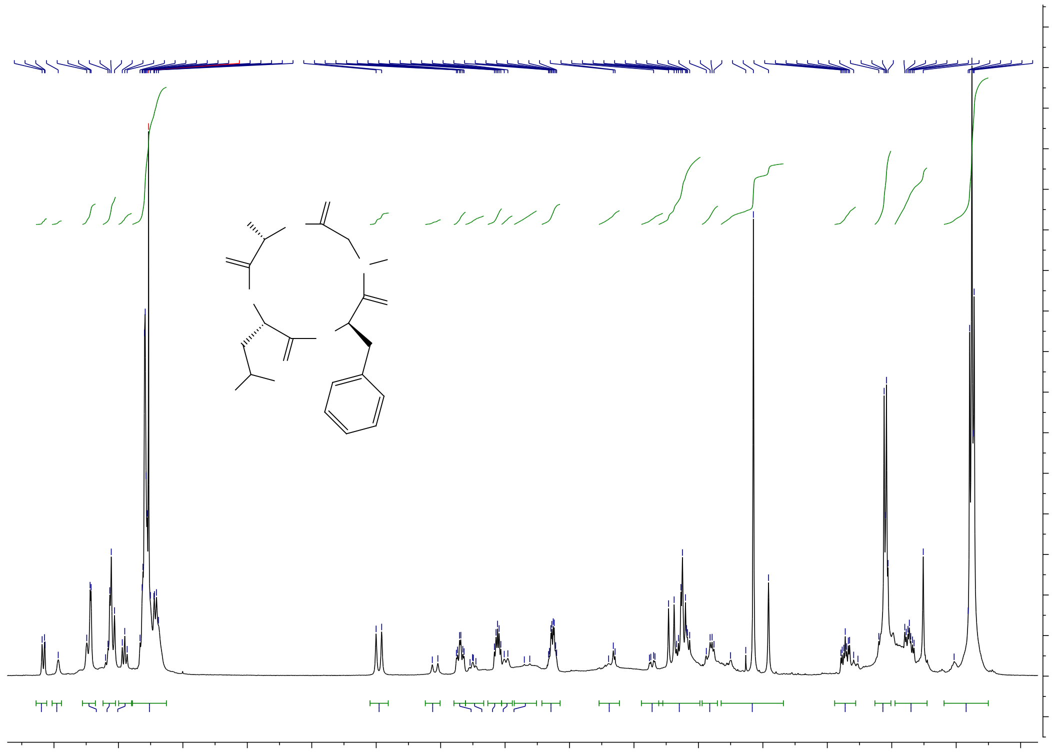

*¹H NMR (400 MHz, CDCl₃) spectrum of **21**.*

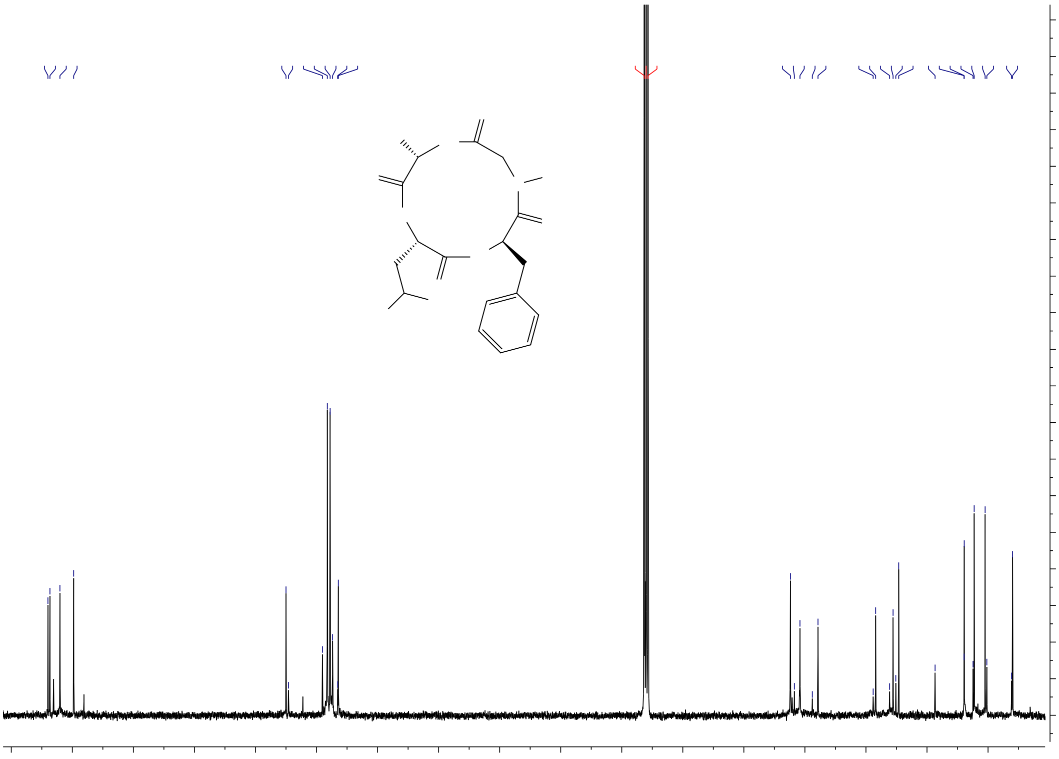

*¹³C NMR (100 MHz, CDCl₃) spectrum of **21**.*

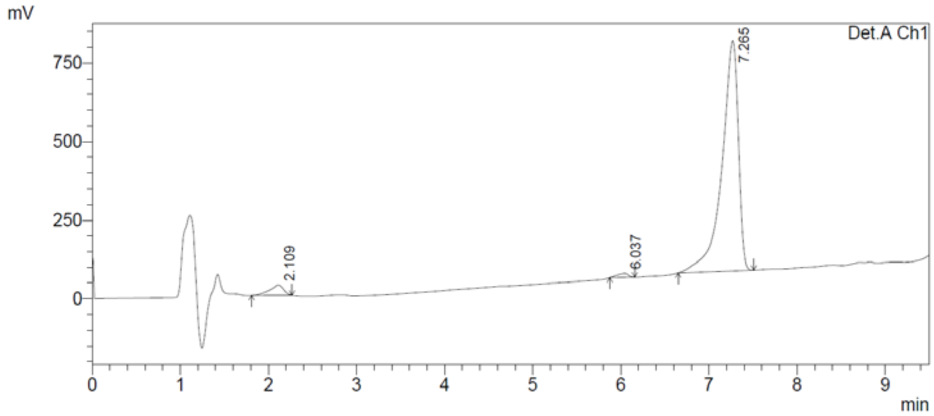

PeakTable

Detector A Ch1 220nm

Peak#	Ret. Time	Area	Height	Area %	Height %
1	2.109	364067	33135	3.647	4.251
2	6.037	110854	13216	1.110	1.695
3	7.265	9507505	733187	95.242	94.054
Total		9982427	779537	100.000	100.000

HPLC Chromatogram of ***21****. Conditions: Linear gradient,* t0´- 30% B, t9´- 90% B, *25ºC, 220 nm. A:* H_2O*, 0.1% formic acid, B: MeCN, 0.1% formic acid.*

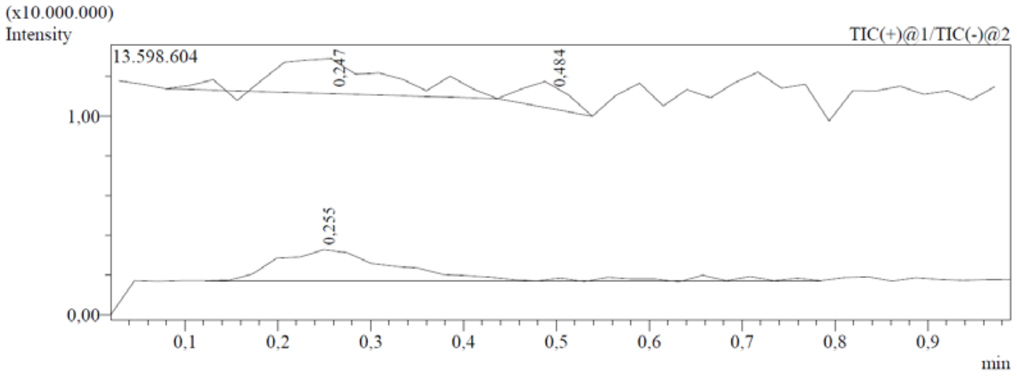

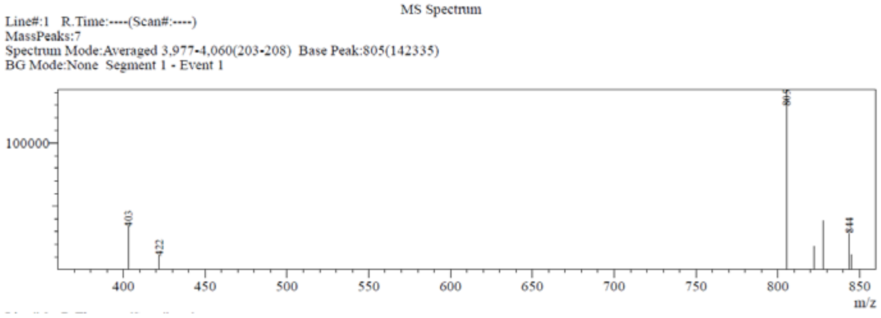

ESI-MS spectrum of ***21****.*

Cyclo-[Ala-Leu-*N*-MePhe-*N*-MeGly] (22)

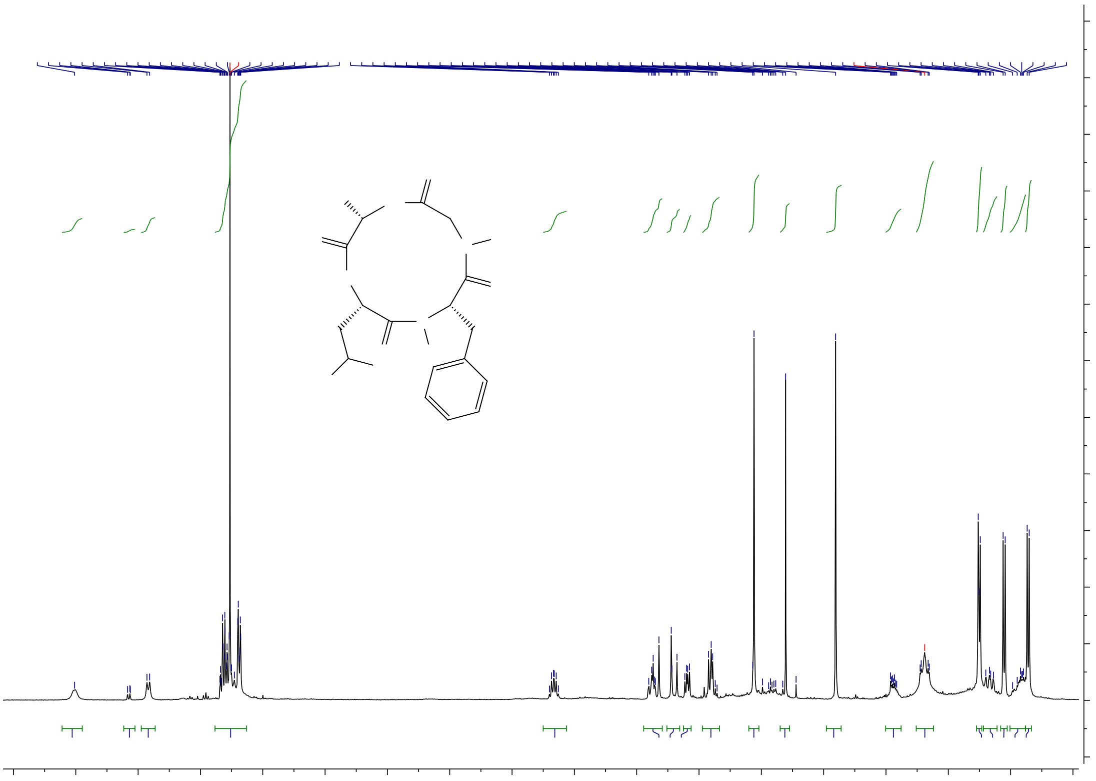

^{1}H NMR (400 MHz, CDCl$_3$) spectrum of **22**.

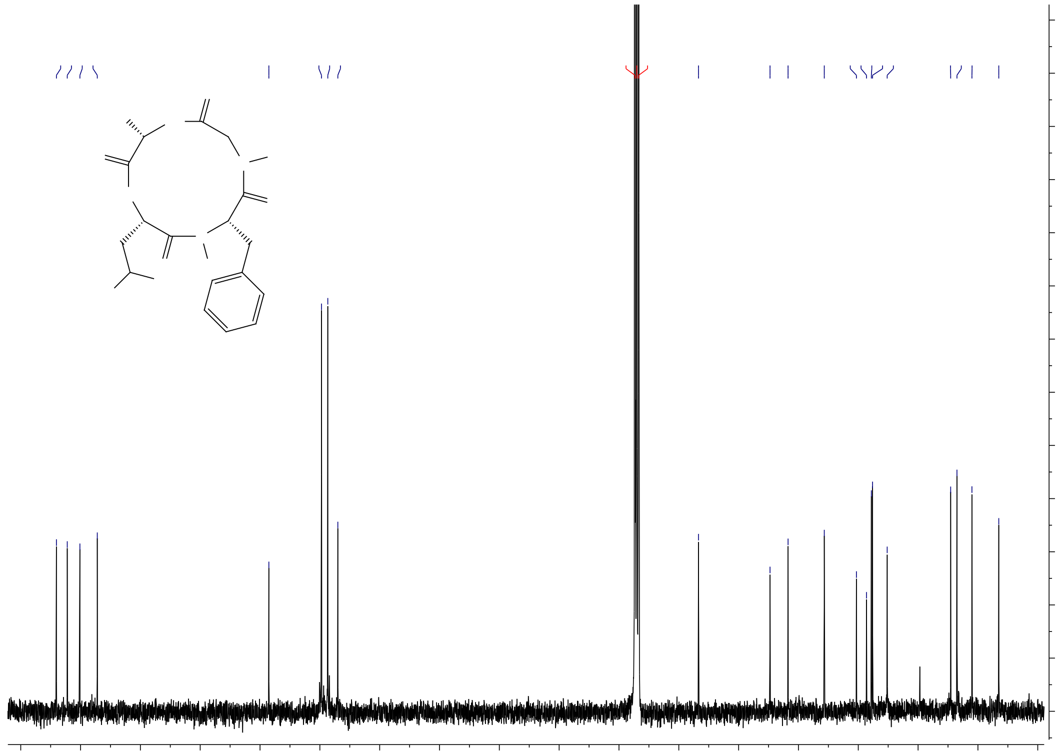

^{13}C NMR (100 MHz, CDCl$_3$) spectrum of **22**.

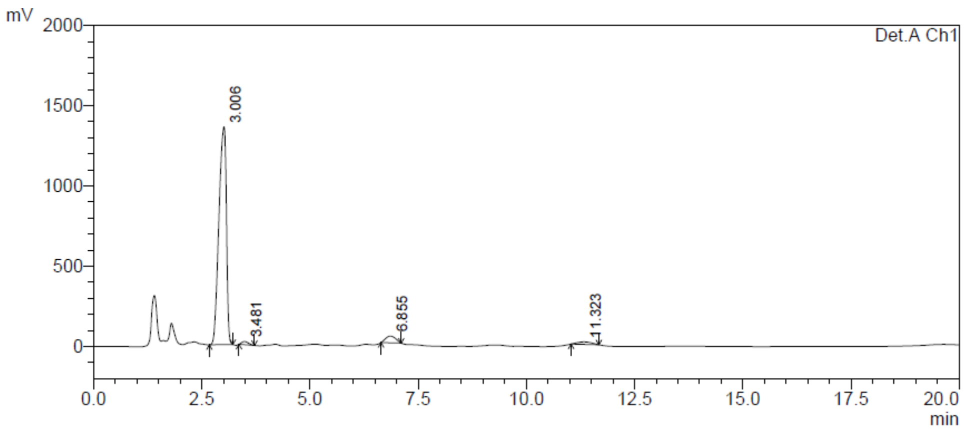

PeakTable

Detector A Ch1 220nm

Peak#	Ret. Time	Area	Height	Area %	Height %
1	3.006	16255009	1356574	92.858	94.635
2	3.481	214134	19758	1.223	1.378
3	6.855	672755	41634	3.843	2.904
4	11.323	363266	15511	2.075	1.082
Total		17505164	1433476	100.000	100.000

HPLC Chromatogram of ***22****. Isocratic flow, MeOH: H_2O (70:30), 30 °C.*

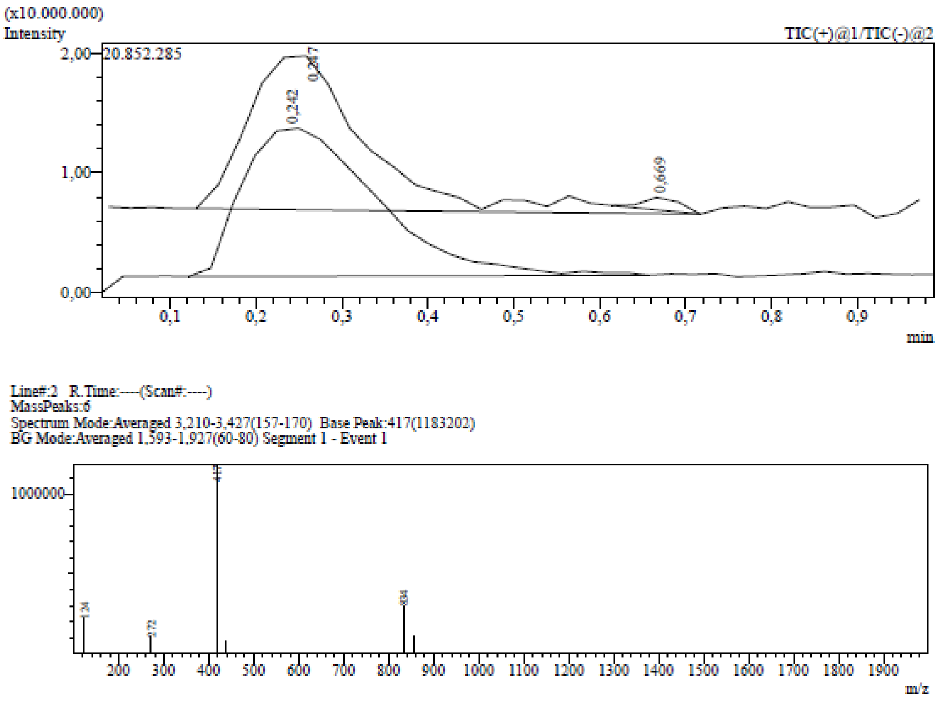

ESI-MS spectrum of ***22****.*

Cyclo-[_N_-MeAla-Leu-Phe-_N_-MeGly] (23)

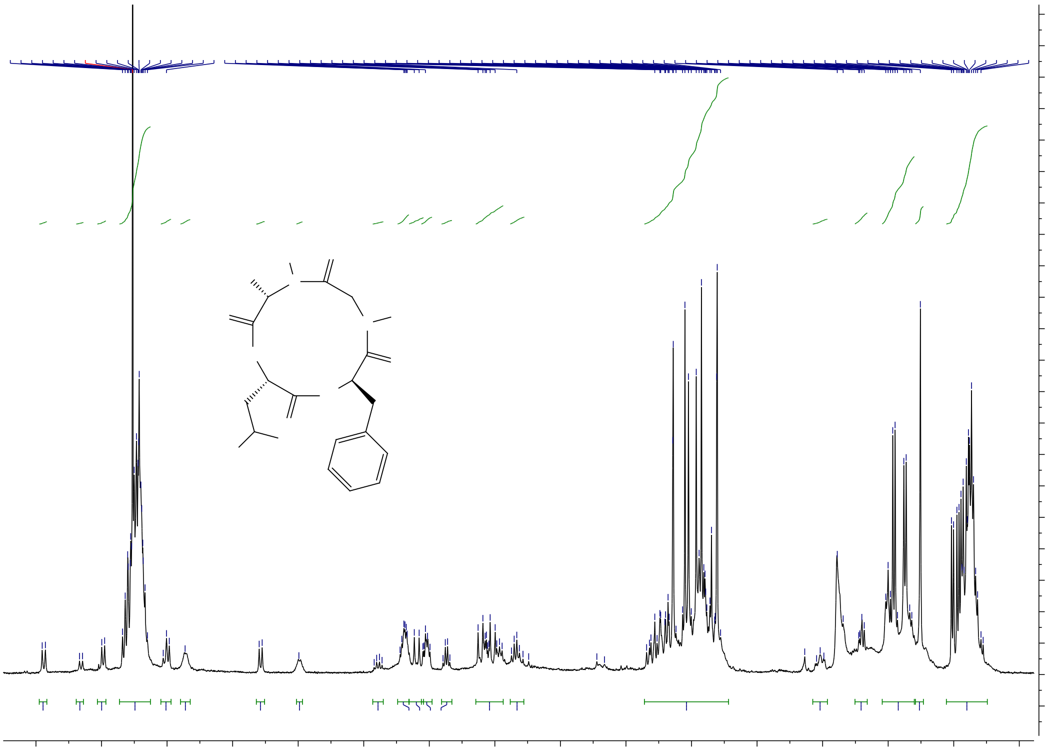

*^{1}H NMR (400 MHz, CDCl3 spectrum of **23.***

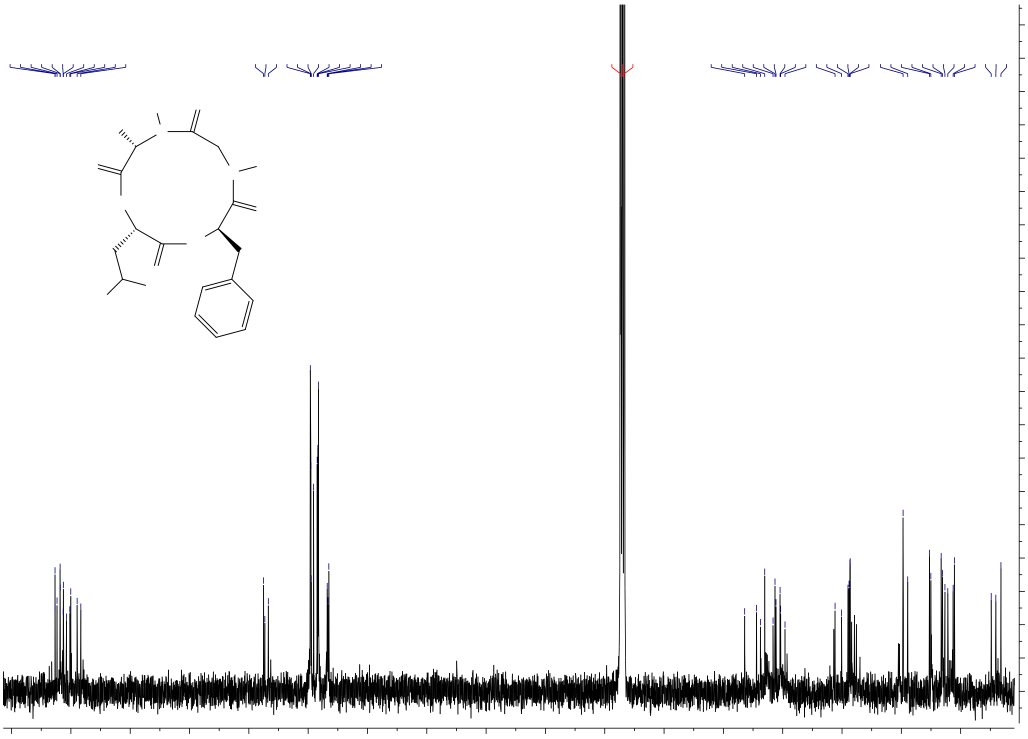

*^{13}C NMR (100 MHz, CDCl3) spectrum of **23***

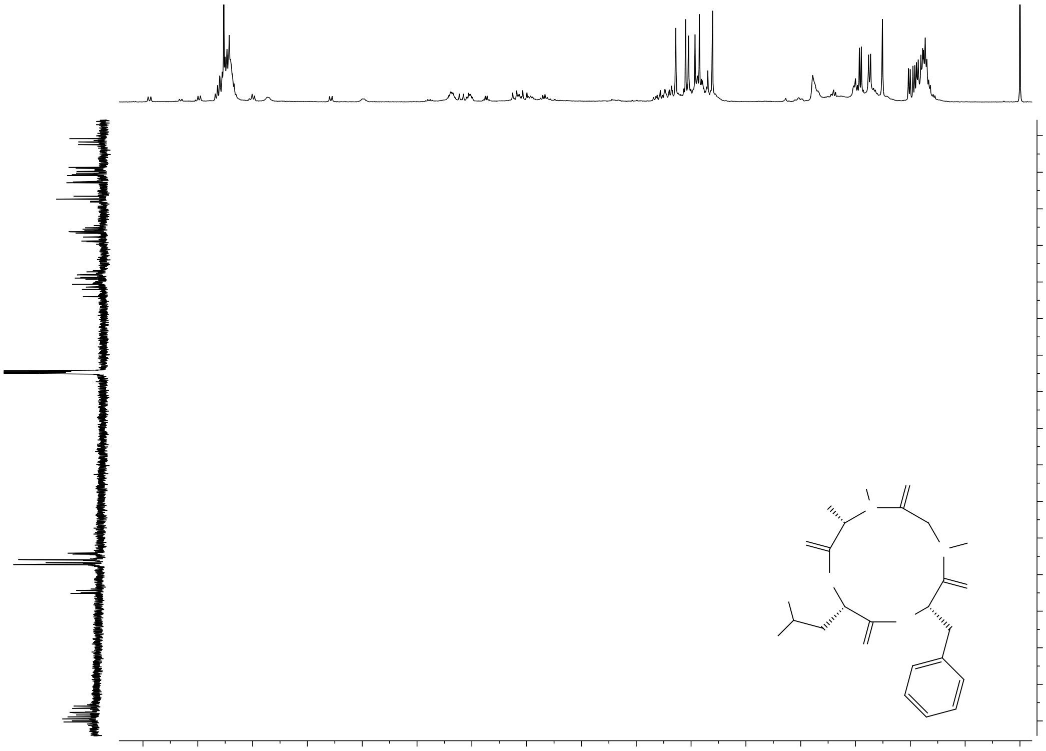

HSQC spectrum of **23**.

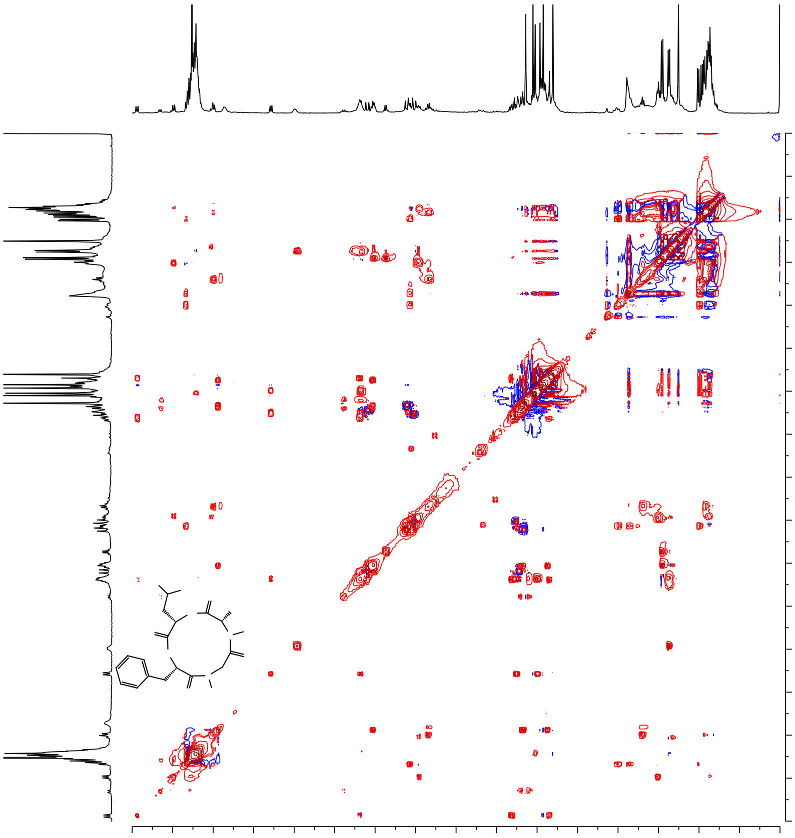

TOCSY ($CDCl_3$) spectrum of **23**.

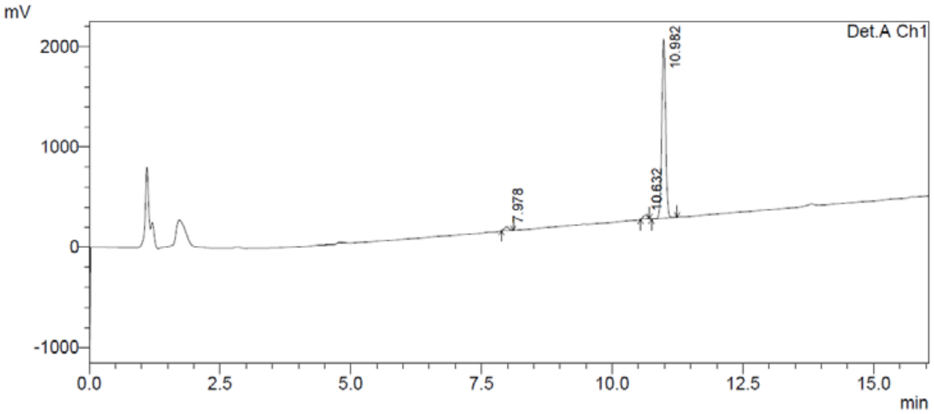

PeakTable

Detector A Ch1 220nm

Peak#	Ret. Time	Area	Height	Area %	Height %
1	7.978	205241	34145	2.162	1.837
2	10.632	218541	37905	2.302	2.039
3	10.982	9067720	1786593	95.535	96.123
Total		9491502	1858644	100.000	100.000

HPLC Chromatogram of ***23****. Conditions: Linear gradient,* t0´- 30% B, t15´- 90% B, *25ºC, 220 nm. A: H_2O, 0.1% formic acid, B: MeCN, 0.1% formic acid.*

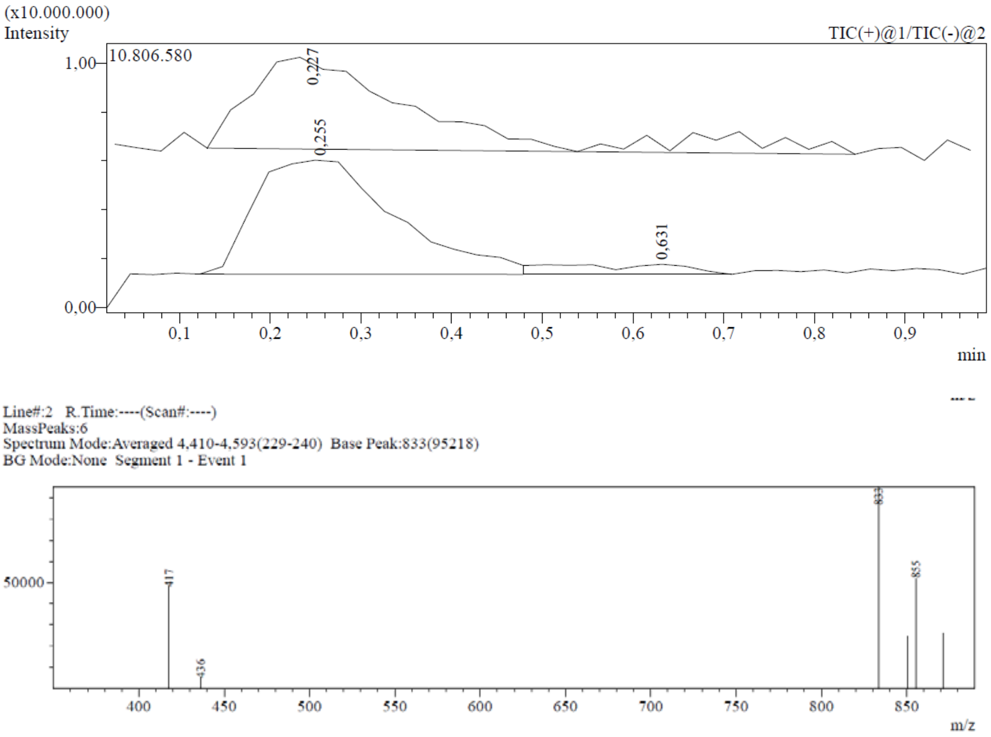

ESI-MS spectrum of ***23****.*

Cyclo-[_N_-MeAla-Phe-_N_-MePhe-Gly] (24)

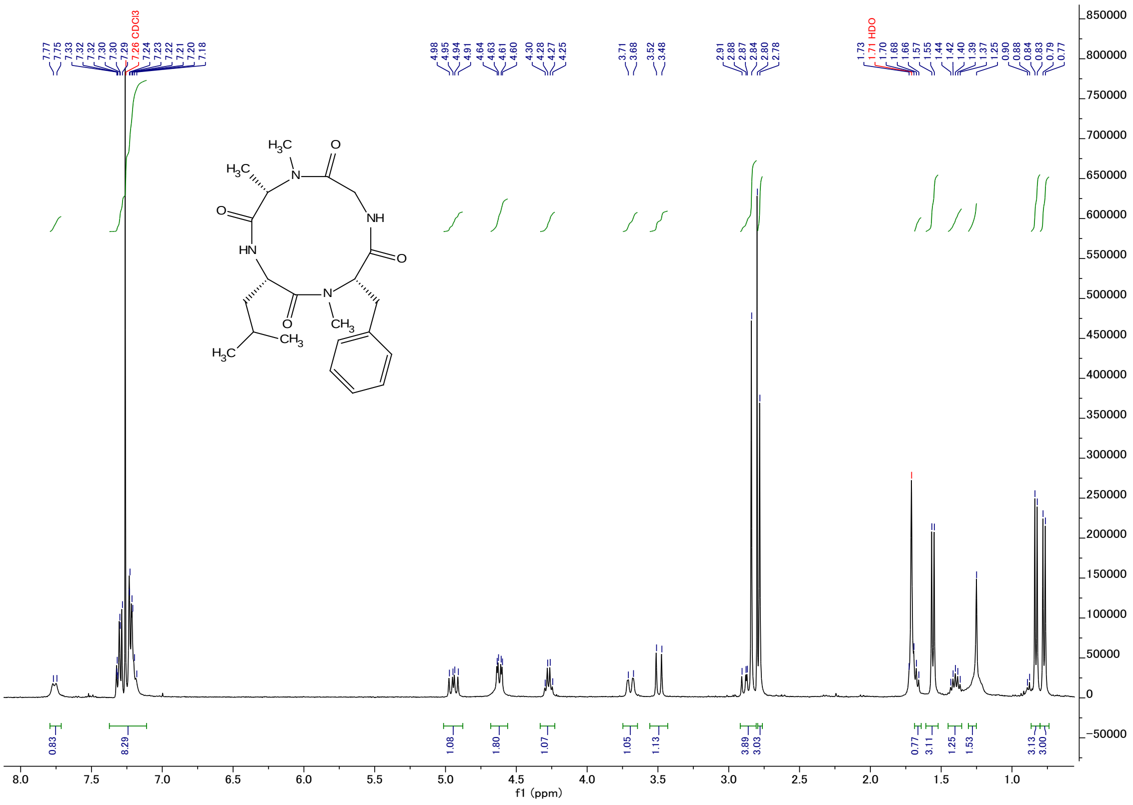

^{1}H NMR (400 MHz, $CDCl_3$) spectrum of ***24.***

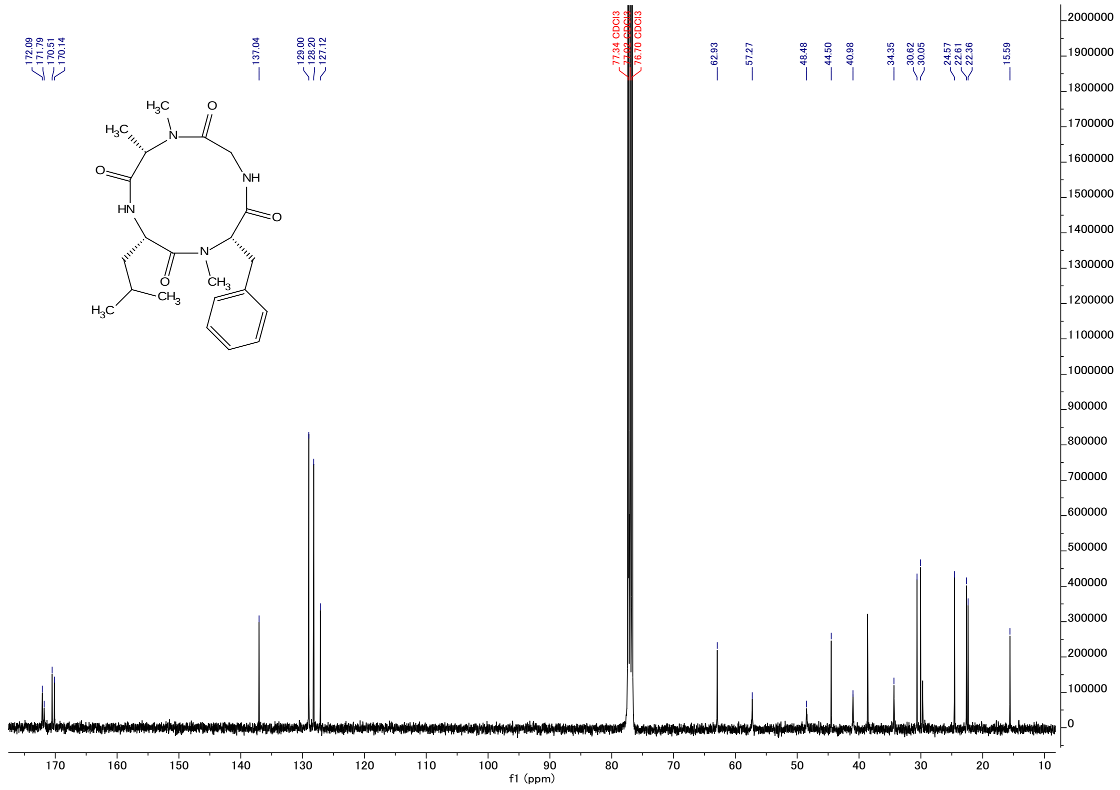

^{13}C NMR (100 MHz, $CDCl_3$) spectrum of ***24.***

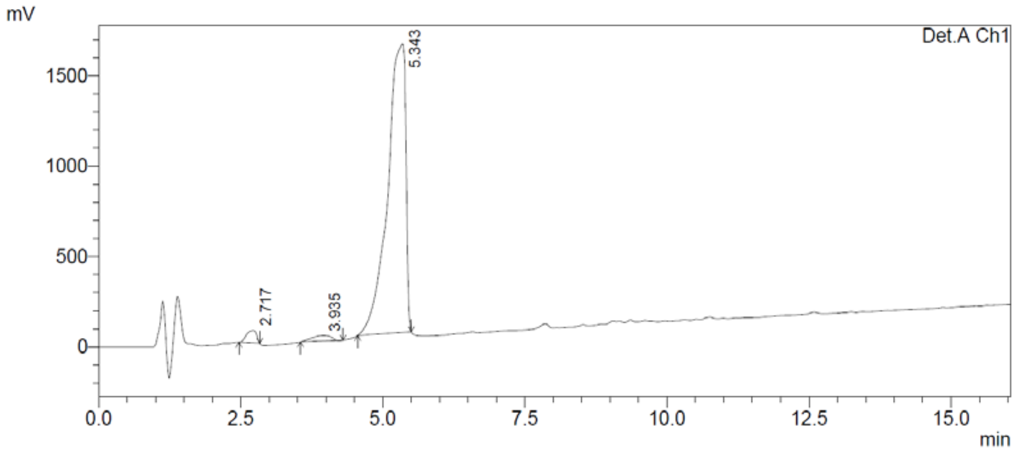

PeakTable

Detector A Ch1 220nm

Peak#	Ret. Time	Area	Height	Area %	Height %
1	2.717	843114	69538	2.280	4.099
2	3.935	693897	30322	1.877	1.788
3	5.343	35435084	1596425	95.843	94.113
Total		36972094	1696284	100.000	100.000

HPLC Chromatogram of ***24****. Conditions: Linear gradient,* t0´- 30% B, t15´- 90% B, *30ºC, 220 nm. A:* H_2O*, 0.1% formic acid, B: MeCN, 0.1% formic acid.*

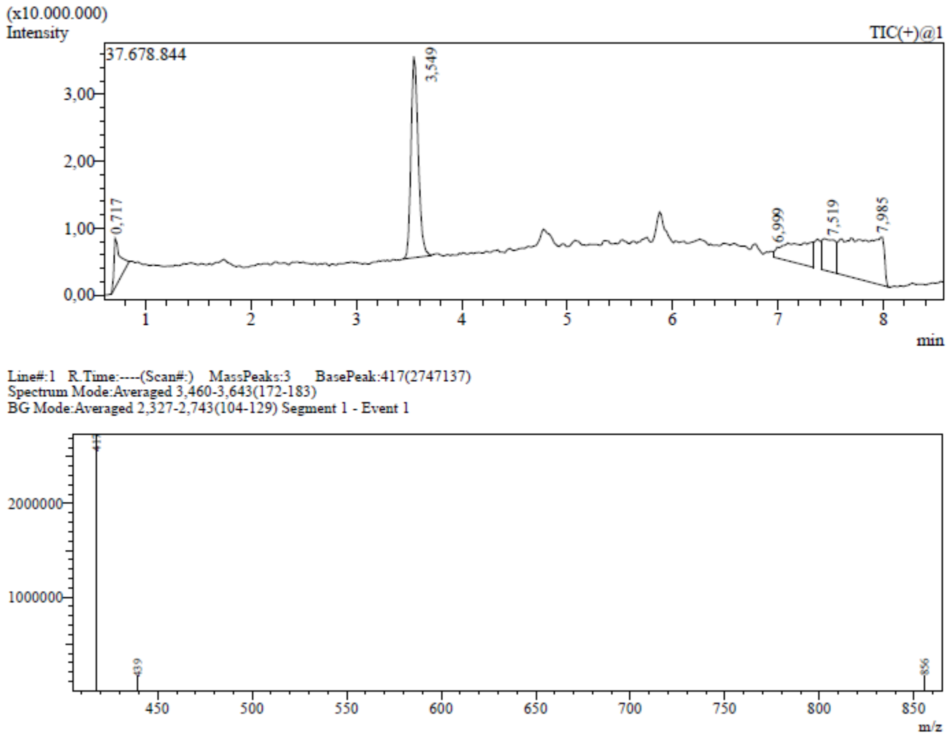

ESI-MS spectrum of ***24****.*

***Cyclo*-[*N*-MeAla-Phe-*N*-MePhe-Gly] (25)**

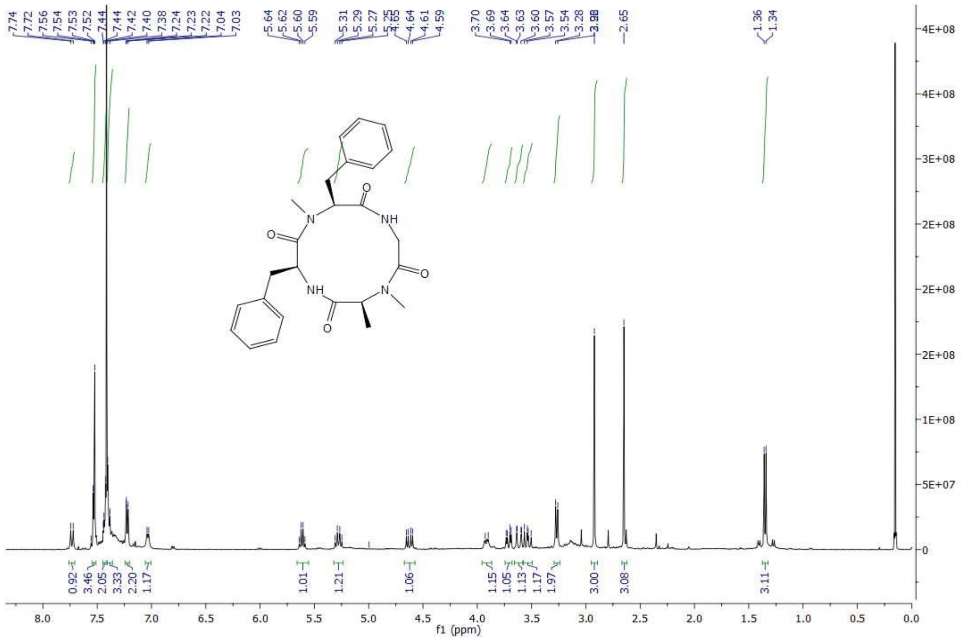

^{1}H NMR (400 MHz, CDCl₃) spectrum of ***25.***

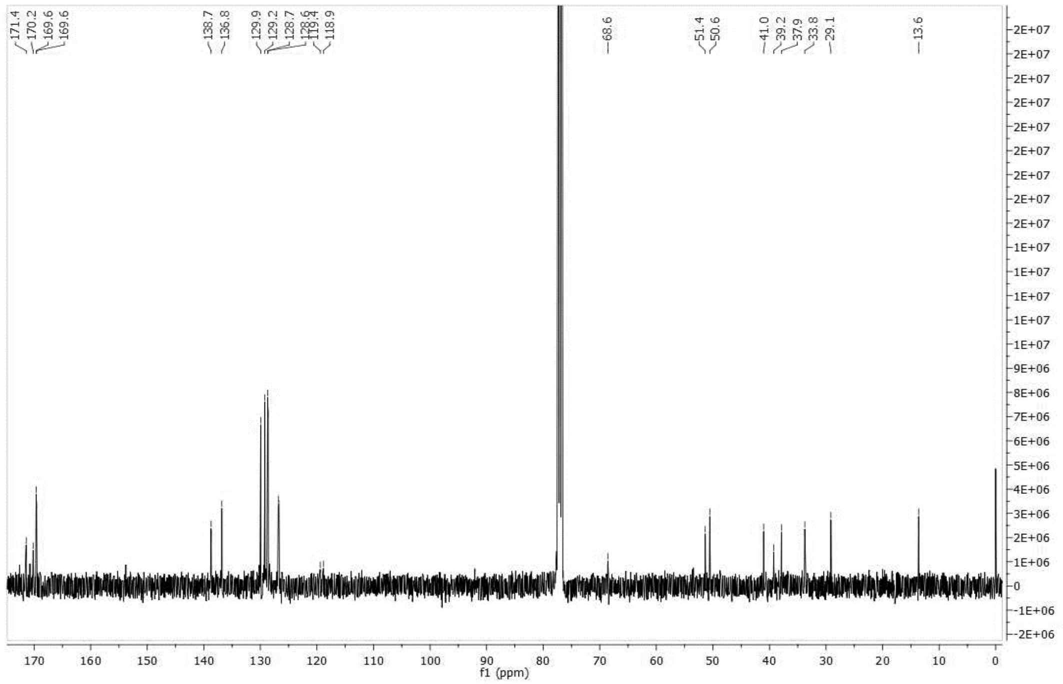

^{13}C NMR (100 MHz, CDCl₃) spectrum of ***25.***

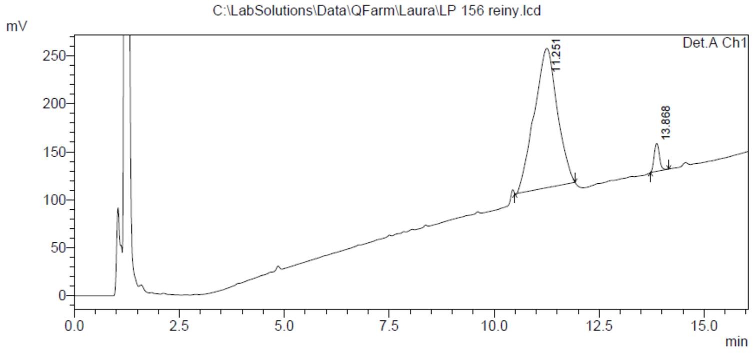

PeakTable

Detector A Ch1 220nm

Peak#	Ret. Time	Area	Height	Area %	Height %
1	11.251	5556157	145358	95.619	83.330
2	13.868	254587	29079	4.381	16.670
Total		5810744	174437	100.000	100.000

HPLC Chromatogram of **25**. *Conditions: Linear gradient,* t0′- 30% B, t15′- 90% B, *30ºC, 220 nm. A: H_2O, 0.1% formic acid, B: MeCN, 0.1% formic acid.*

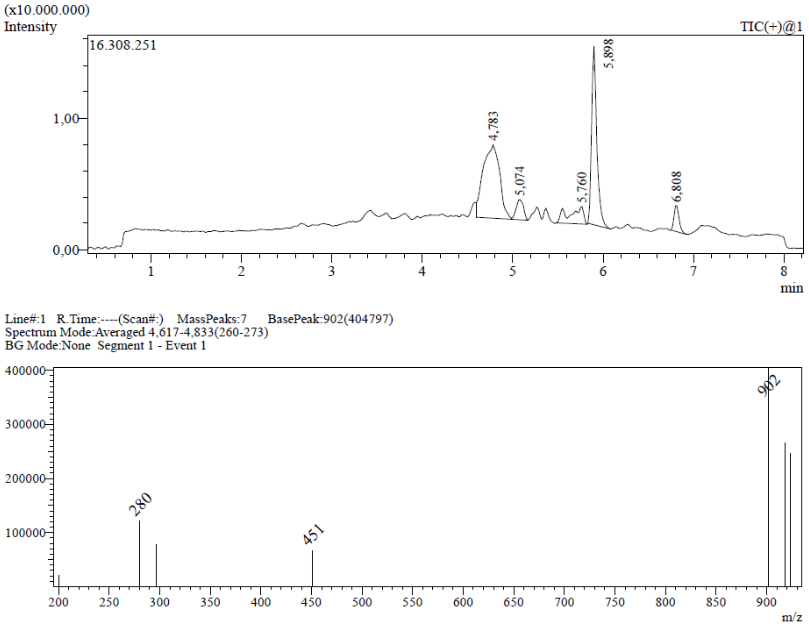

ESI-MS spectrum of **25.**

Cyclo-[N-Me-D-Phe-Ala-Phe-N-Me-Gly] (26)

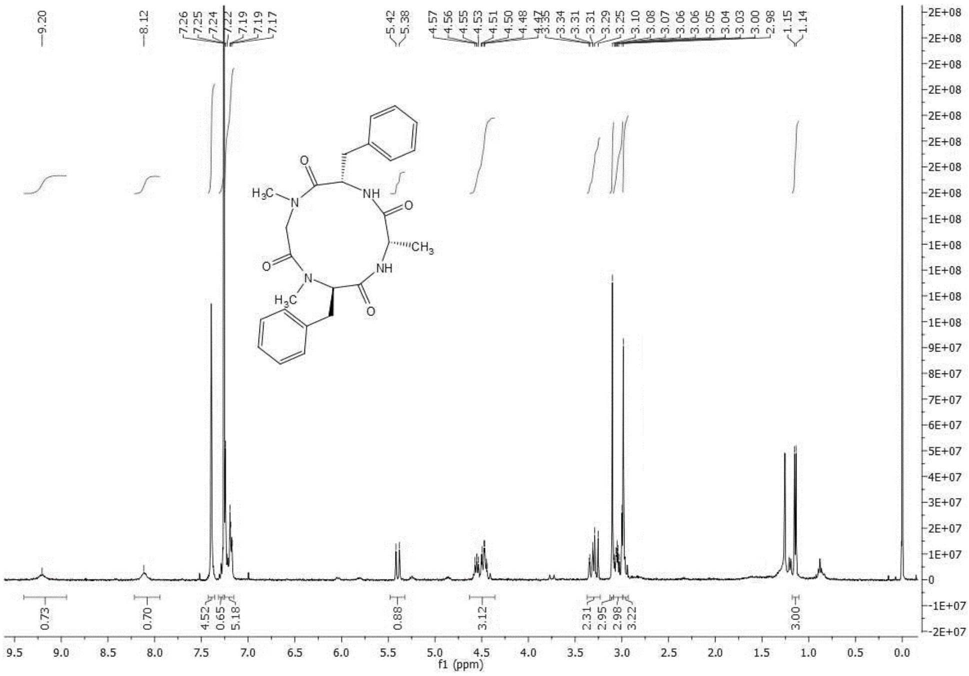

^{1}H NMR (400 MHz, CDCl$_3$) spectrum of ***26****.*

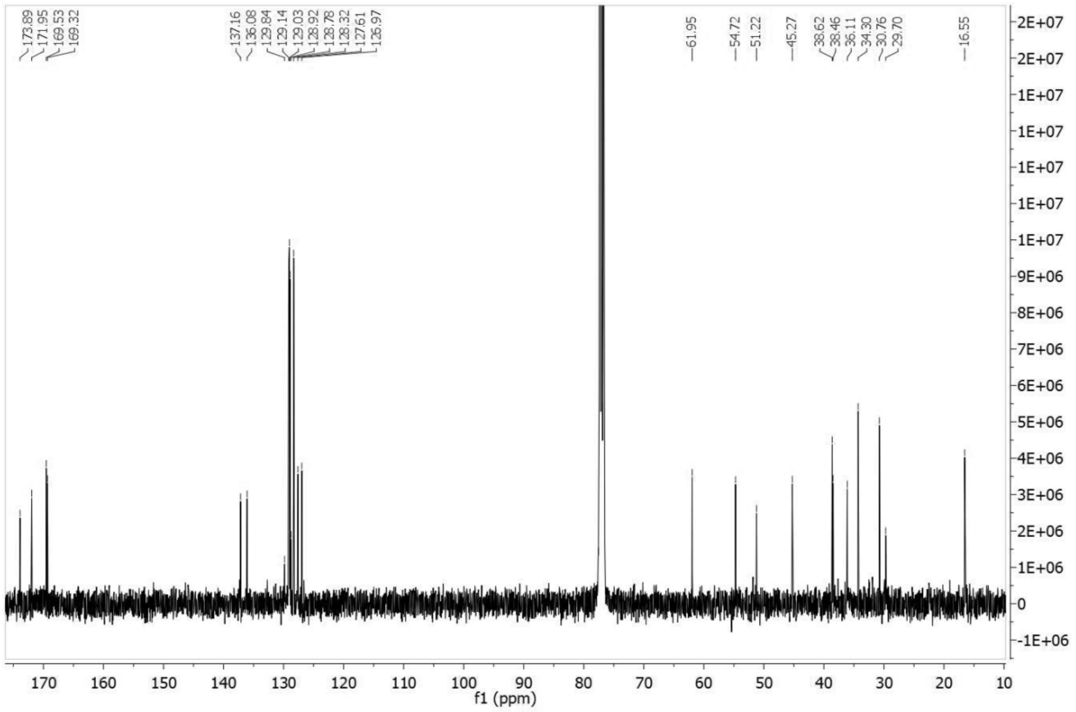

^{13}C NMR (100 MHz, CDCl$_3$) spectrum of ***26****.*

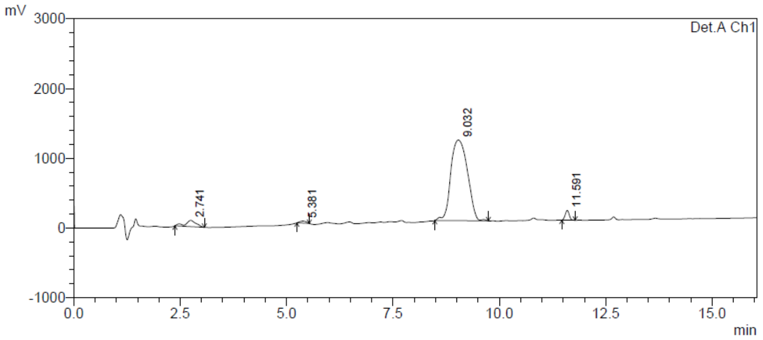

PeakTable

Detector A Ch1 220nm

Peak#	Ret. Time	Area	Height	Area %	Height %
1	2.741	1565594	89413	4.544	6.318
2	5.381	308312	28610	0.895	2.022
3	9.032	31666788	1157186	91.914	81.772
4	11.591	911786	139925	2.647	9.888
Total		34452480	1415134	100.000	100.000

HPLC Chromatogram of ***26****. Conditions: Linear gradient,* t0´- 30% B, t15´- 90% B, *30ºC, 220 nm. A: H_2O, 0.1% formic acid, B: MeCN, 0.1% formic acid.*

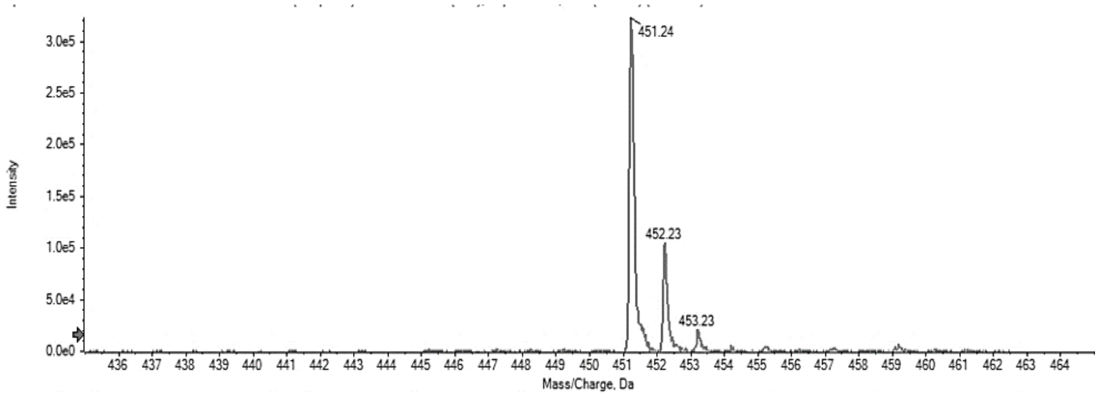

ESI-MS spectrum of ***26****.*

Cyclo-[Phe-N-MeGly-Cys(Bn)-N-MeGly] (27)

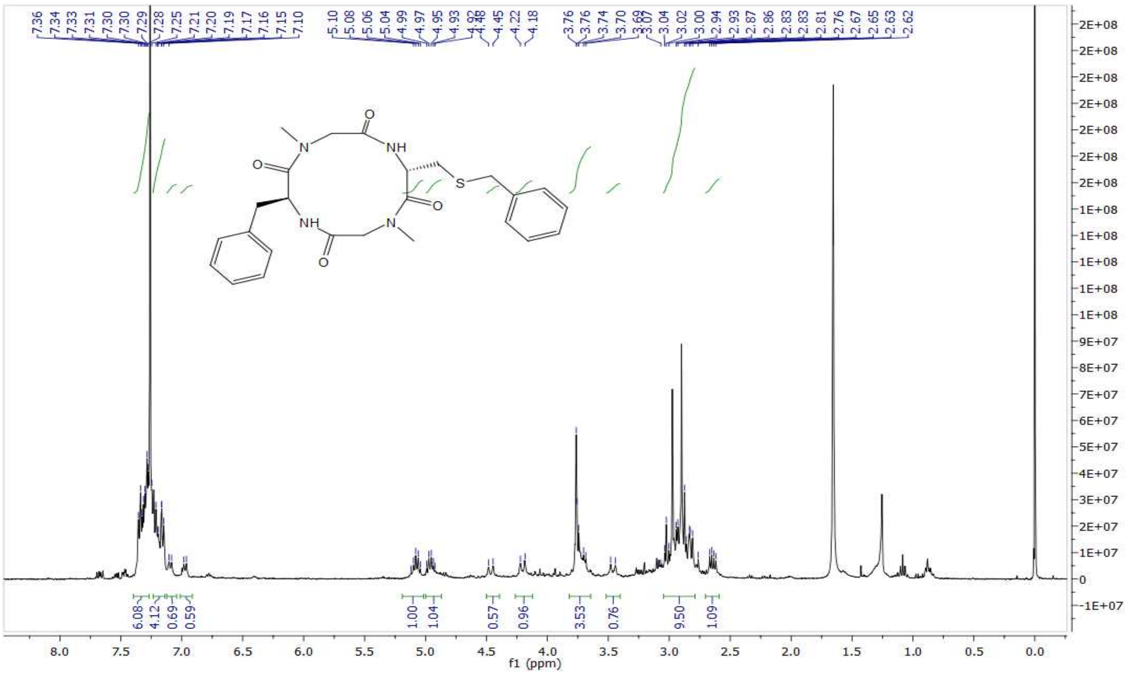

^{1}H NMR (400 MHz, $CDCl_3$) spectrum of ***27.***

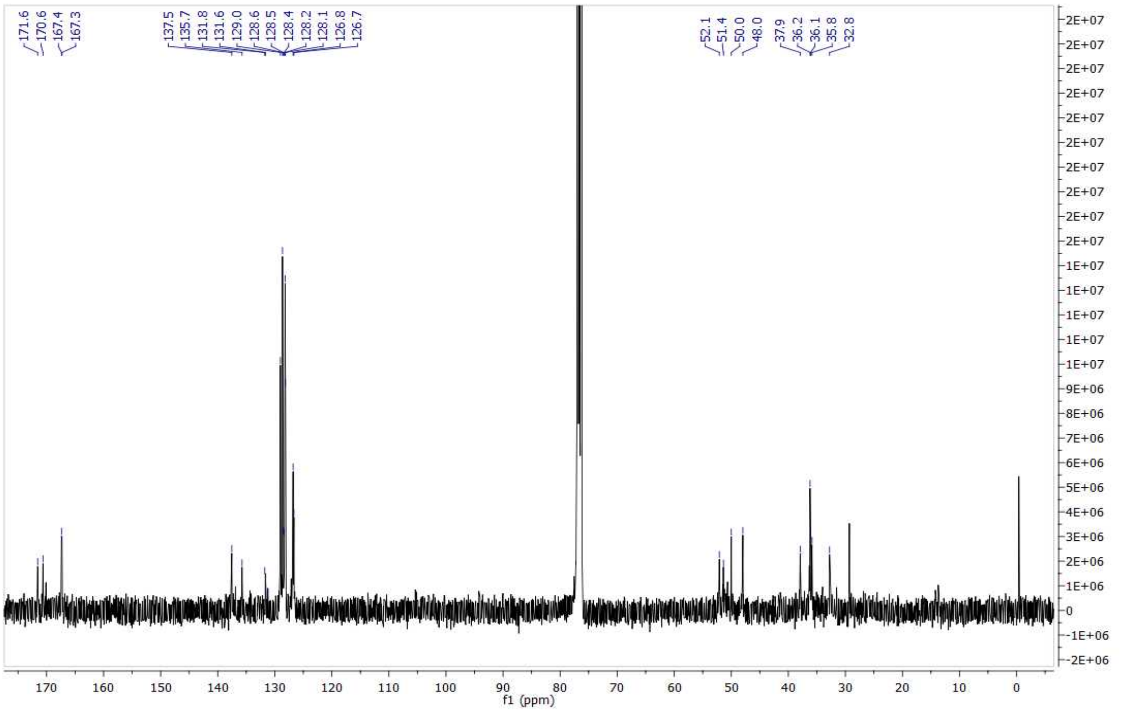

^{13}C NMR (100 MHz, $CDCl_3$) spectrum of ***27.***

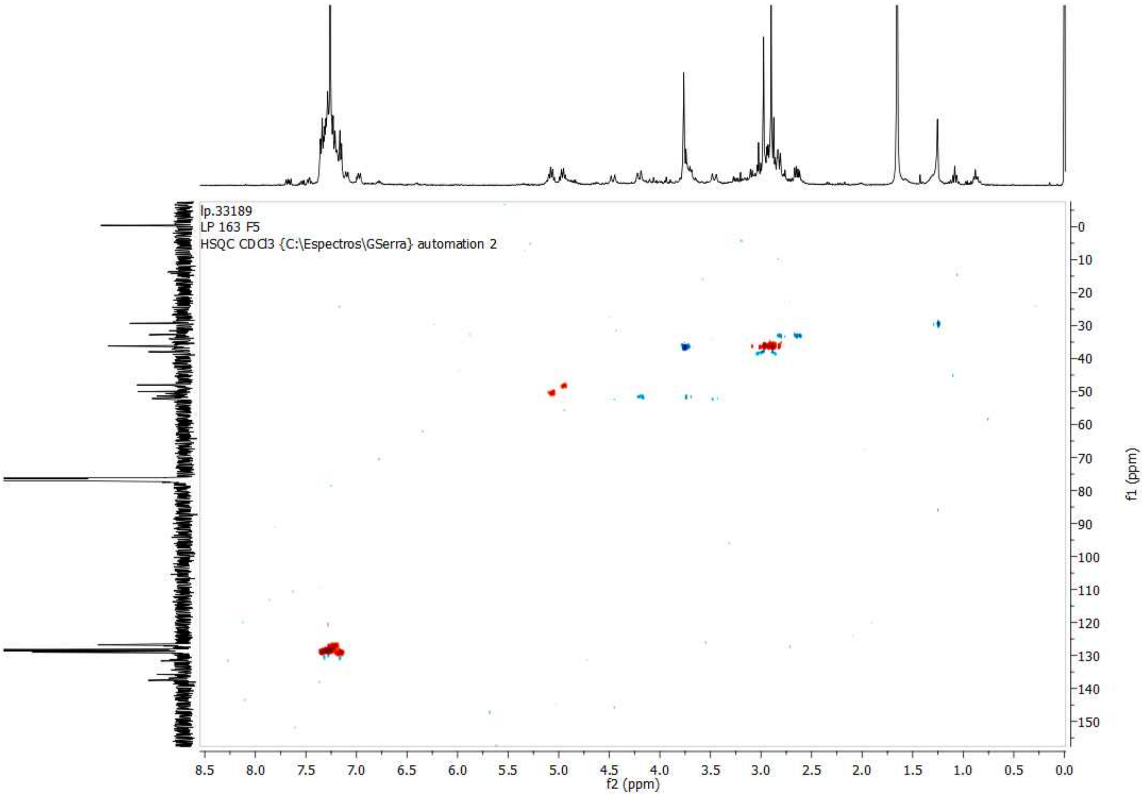

*HSQC (CDCl₃) spectrum of **27.***

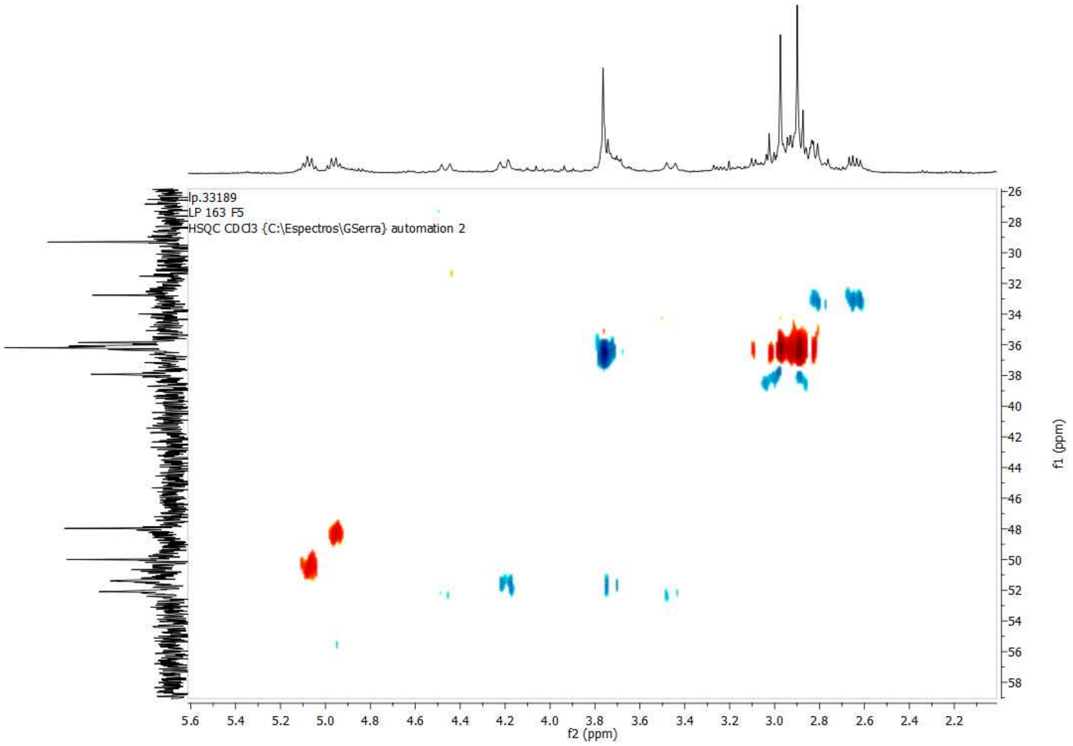

*HSQC (10 to 65 ppm ^{13}C vs 0.4 to 5.6 ppm ^{1}H), spectrum of **27.***

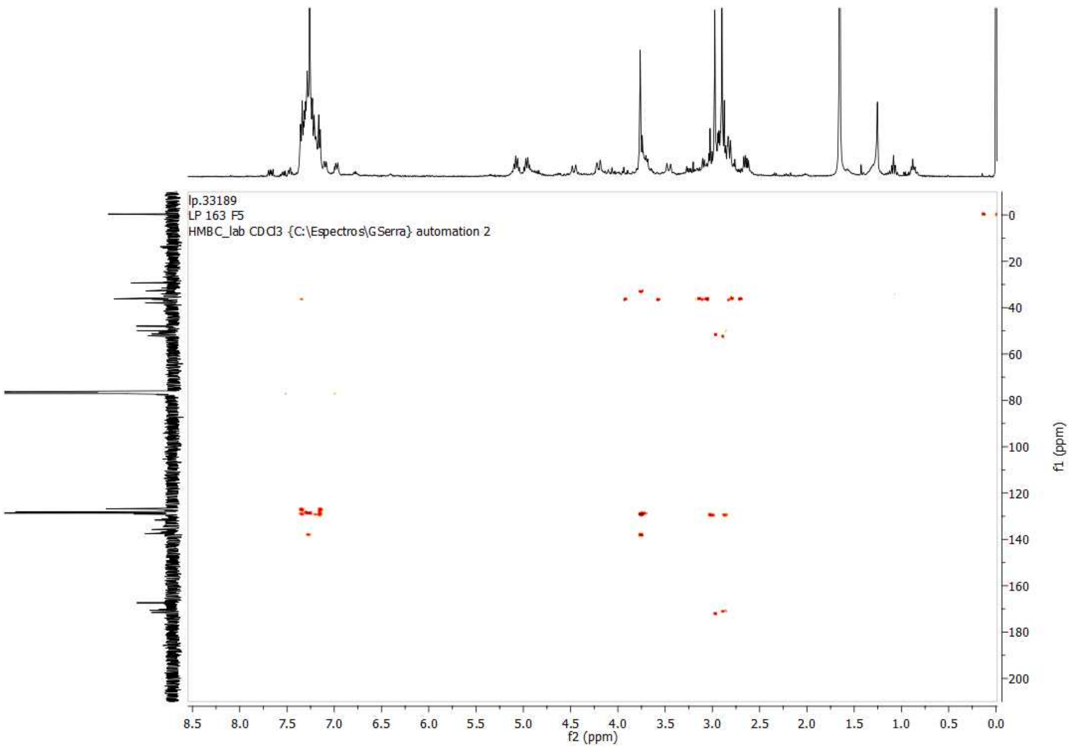

HMBC ($CDCl_3$) spectrum of **27.**

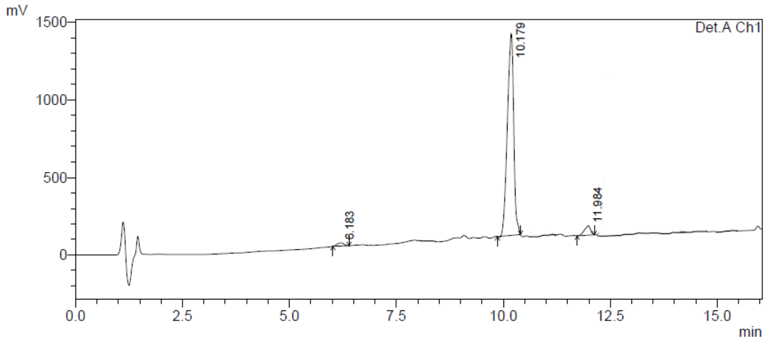

PeakTable

Detector A Ch1 220nm

Peak#	Ret. Time	Area	Height	Area %	Height %
1	6.183	216359	19844	1.515	1.433
2	10.179	13471371	1305110	94.316	94.217
3	11.984	595557	60266	4.170	4.351
Total		14283287	1385220	100.000	100.000

HPLC Chromatogram of **27**. *Conditions: Linear gradient,* t0´- 30% B, t15´- 90% B, *30ºC, 220 nm. A: H_2O, 0.1% formic acid, B: MeCN, 0.1% formic acid.*

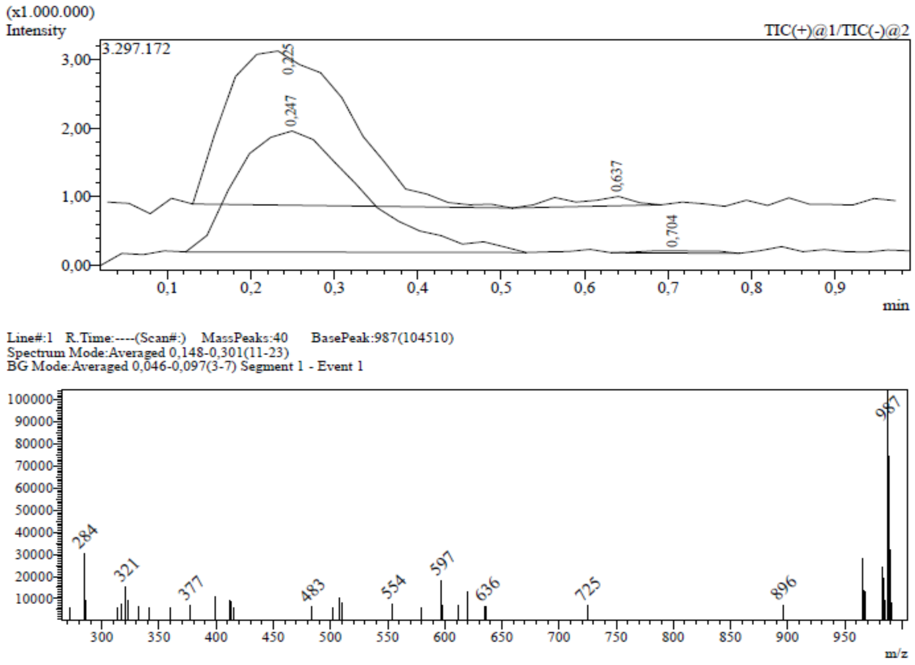

ESI-MS spectrum of ***27.***

5. Procedure for evaluation of phytotoxic activity

Experiments for determining the herbicidal activity of cyclopeptide compounds were carried out on *Lolium multiflorum* (Ray grass) plants. Germination, root length, and leaf development were evaluated compared to a control without herbicide, a negative control (with DMSO, used as solvent), and an herbicide control (1/8 of the commercial dose of the herbicide *S*-metolachlor).

5.1. Methodology

Serial experiments were conducted using the Agar germination methodology, where the tested compounds and the respective controls were placed in glass Petri dishes (6 cm diameter) in three replicates per treatment. Ten Ryegrass seeds were germinated in a growth chamber (20 °C, day/night temperature). The seeds were previously sterilized by immersing them in 70% alcohol for 10 seconds. When distributed in the Petri dish on the agar, the seeds were placed in such a way as to ensure that they remained submerged in the solution.

An agar–water solution was prepared at 0.3%, 3 g of Agar was placed in 1 liter of deionized water, and the solution was autoclaved at 100 °C for 45 min. Once the agar medium had cooled to approximately 60°C, the solutions were prepared.

The negative control—DMSO control—was prepared by adding 100 μL of DMSO per plate in 15 mL of agar, and then the seeds were distributed as mentioned above. A control without DMSO was also carried out to check that the product was not altering the correct development of Rye grass seeds. For this test, 15 mL of agar was placed in each Petri dish and, before it solidified, the seeds of the species evaluated were placed on top. The herbicide treatment for the positive control—Control *S*-Metolachlor (960 g/L)—was carried out for a conversion of 1/8 of the dose of 1 L/ha of commercial product. For this purpose, a stock solution of *S*-Metolachlor was prepared by placing 0.28 mL of the herbicide in a volumetric flask and topping up to 1000 mL. Twenty-five milliliters of this stock solution was taken, placed in a volumetric flask and brought to 200 mL, thus generating the 1/8x solution of *S*-Metolachlor. A volume of 3 mL of 1/8x herbicide solution was mixed with 45 mL of the agar solution to bring 16 mL into each Petri dish. This ensured that there was 1 mL of 1/8x *S*-Metolachlor solution per plate: 0.28 μL of herbicide solution. Seeds were arranged in the same way. Using the same method, the agar media corresponding to each plate (15 mL) were mixed with the cyclopeptides diluted in 100 μL of DMSO.

Germination, root length, and leaf development were evaluated 12 days after preparation. The variables germinated plants and plants with developed leaves of the total number of plants placed to germinate were analyzed by fitting a generalized linear model since they presented a binomial distribution.

Glinmix procedure of the SAS statistical package. Based on the model and for the comparison of treatments with different controls, the contrasts of interest were carried out. The effect of the treatments on the root length variable was studied by comparing means using Tukey's test (p-value < 0.05) in INFOSTAT.
